# Origin and genetic variability of populations of the invasive plant *Rumex alpinus* L. in the Giant (Krkonoše) Mountains

**DOI:** 10.1002/ece3.10145

**Published:** 2023-06-04

**Authors:** Michaela Jungová, Vladimíra Müllerová Jurasová, Petra Hlásná Čepková, Leona Leišová Svobodová, Pavel Svoboda, Michal Hejcman

**Affiliations:** ^1^ Department of Ecology, Faculty of Environmental Sciences Czech University of Life Sciences Prague Prague Czech Republic; ^2^ Crop Research Institute Prague Czech Republic; ^3^ Faculty of Environment Jan Evangelista Purkyně University in Ústí nad Labem Ústí nad Labem Czech Republic

**Keywords:** alpine dock, genetic variability, invasive plant, microsatellite, weed species

## Abstract

Monk's rhubarb, *Rumex alpinus* L. (*R. alpinus*), is a perennial plant native to the mountains of Central and Southern Europe. Currently, the distribution of *R. alpinus* has been partly affected by its utilization as a vegetable and a medicinal herb. In the mountains of the Czech Republic, it is considered an invasive plant, probably introduced into the Krkonoše Mountains by colonists from the Alps. This study's main aim was to verify whether *R. alpinus* was introduced into the Krkonoše Mountains by alpine colonists or whether it was anthropogenically introduced from the Carpathians. Furthermore, the genetic structure of native and introduced populations of *R. alpinus* was determined. For the evaluation of genetic structure, 417 samples of *R. alpinus* were collected from the Alps, Carpathians, Balkan, Pyrenees, and Czech Mountains. In total, 12 simple sequence repeat (SSR) markers were applied. The results of AMOVA showed a high 60% variation within populations, 27% variation among groups, and 13% among the population within groups. The overall unbiased gene diversity was high (^*ĥ* = 0.55). The higher level of genetic differentiation among populations (FST = 0.35; *p* < .01) indicated restricted gene flow between populations. Compared to native populations, limited genetic variability was observed in the nonnative populations. It was concluded that local adaptation, low gene exchange, and genetic drift affected the genetic diversity of nonnative *R. alpinus*. The results support a genetic link between Alpine and Czech genotypes of *R. alpinus*, while the Carpathians genotypes corresponded to the Balkan genotype.

## Graphical abstract

This study's main aim was to verify whether *Rumex alpinus* was introduced into the Krkonoše Mountains by alpine colonists or whether it was anthropogenically introduced from the Carpathians. Furthermore, the genetic structure of native and introduced populations of *Rumex alpinus* was determined.

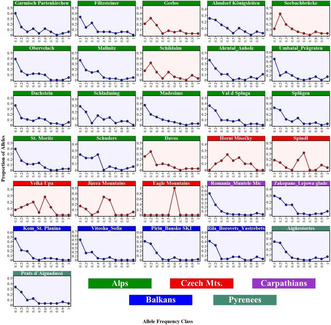

## 1 INTRODUCTION

Monk's rhubarb (*Rumex alpinus L*.) is native to the high mountains of Western, Central, and Eastern Europe, including the Iberian and Balkan Peninsulas and the East and Western Carpathians (Št'astná et al., 2010). In the Krkonoše (Giant) Mountains, *R. alpinus* was probably introduced by German‐speaking colonists from the Alps in the 16th century AD and used for the treatment of different diseases. Boiled plants were used as a fodder crop for pigs and goats (Brockmann‐Jerosch, 1921; Kopecký, 1973; Kubát, 1990; Lokvenc, 1978; Št'astná et al., 2010). The leaves were used for packing butter in Tyrol and in some parts of the Carpathians (Kopecký, 1973). Seeds, roots, and rhizomes of *R. alpinus* have also been used for the treatment of several health disorders, such as diarrhea, dysentery, stomach problems, and kidney disorders. Powder, decoction, infusion, poultice, and ointments prepared from the roots, seeds, leaves, or whole plants have also been used for the treatment of different types of tumors (Bogl et al., 2013; Hartwell, 1970; Jang et al., 2012; Vasas et al., 2015).

Despite its medicinal properties and uses, *R. alpinus* is a troublesome weed and invasive plant in some mountain areas of Europe, growing in permanent monodominant stands characterized by low natural conservation and agricultural value (Delimat & Kiełtyk, 2019). Due to its successful dissemination, other plant species are suppressed by shading and “fast casting” (the ability of these invasive plants to grow and spread quickly) of above ground and underground parts (Bohner, 2005; Raycheva & Dimitrova, 2007). Some invasive plant species have adaptations that allow them to outcompete native plants, such as rapid growth, early leaf‐out, efficient nutrient uptake, or release of chemicals that inhibit the growth of other plants. This can lead to the displacement of native plant species and reduced biodiversity in the affected area (Št'astná et al., 2010). The overshadowing effect of large leaves and horizontal development of *R. alpinus* rhizomes create such difficult conditions that only a few species (*Urtica dioica* L., *Deschampsia caespitosa* L., *Chaerophyllum hirsutum* L., and *Stellaria nemorum* L.) are capable of surviving on these sites (Stachurska‐Swakoń, 2009).

*Rumex alpinus* was found to be an invasive neophyte in the Czech Republic in recent decades (Pyšek et al., 2012), invading many localities in the Krkonoše (Giant) Mountains (Náglová et al., 2014). Its ability to supplant original species leads to ecosystem disbalance (Bohner, 2005; Delimat & Kiełtyk, 2019; Pyšek et al., 2012). Plants of *R. alpinus* occur in nutrient‐rich habitats such as mountain pastures, particularly in overfertilized locations, around mountain huts and roads, and along riverbanks (Bohner, 2005; Delimat & Kiełtyk, 2019; Klimeš, 1992; Št'astná et al., 2010). It is an anemophilous plant (Kubát, 1990); however, the pollen produced by flowering plants attracts numerous pollen‐feeding insects, thereby taking part in the gene flow of *R. alpinus* populations (Klimeš, 1994; Št'astná et al., 2010). The production of seeds is very high (Št'astná et al., 2010); a flowering plant can produce approximately 11,500 seeds m−2 (Klimeš, 1992). The seeds can remain dormant for many years (Bucharová, 2003), and below the stand is a wealthy seed bank (Št'astná et al., 2010). The seeds are spread over long distances (100 m), mainly downstream, allowing the colonization of new habitats (Červenková & Münzbergová, 2009). Moreover, *R. alpinus* is also a clonal plant that reproduces through rhizomes (Klimeš, 1992), and the growth rate of populations is high and fast (Klimeš et al., 1993).

Whether a plant is native or nonnative in a given area is often difficult to determine. The allochthonous origin of many archaeophytes, epoecophytes, and ephemerophytes, e.g., *Ballota nigra* L. Subsp. Nigra and *Verbena officinalis* L., is associated only with anthropogenically influenced communities (Kopecký, 1973; Pyšek et al., 2012). The distribution of some of them, such as *Lamium album* L. and *Chenopodium bonus‐henricus* L., precisely defined the area of the original Czech agricultural settlement (Kopecký, 1973). However, the connection of a species to communities of anthropogenic origin may not provide adequate evidence for its allochthonous origin (Chytrý et al., 2005; Kopecký, 1973; Pyšek et al., 2012). According to Lokvenc (1978), this is the case for the species that have been previously collected as medicinal herbs, and they were also grown in gardens (*Angelica archangelica L*.). Therefore, it is difficult to determine the origin of some species that may have been grown as medicinal or valuable plants in the past.

The literature on the historical colonization of the Úpa and Elbe valleys in the Krkonoše Mountains by settlers from the Alps and their introduction of *R. alpinus* is well‐established (Hendrych, 2001; Kopecký, 1973; Kubát, 1990; Lokvenc, 1978). Moreover, in the past decade, Professor Klimeš has been investigating the genetic origins of the Krkonoše settlers. His research has revealed that these ancestors hailed from sites in Styria's Salzkammergut region and South Tyrol in Austria and Italy (Klimeš, 2011). Whether *R. alpinus* is truly nonnative in the Krkonoše Mountains may not be certain. In Poland, among others, besides the Carpathian Mountains, where *R. alpinus* is a native plant (Klimeš, 1992; Stachurska‐Swakoń, 2009), there is also a part of the Giant Mountains (Karkonosze), and according to Kwiatkowski (2003), *R. alpinus* is a native plant in Poland. Based on this statement and based on the available information, we decided to verify the origin of *R. alpinus* using SSR markers, because the possibility of a different origin of *R. alpinus* populations found in the Giant Mountains is considered. And we pose the following hypotheses:

(i) *Rumex alpinus*, whose European distribution is determined by human activity, was introduced into the Czech part of the Krkonoše Mountains via the Polish part of the Krkonoše Mountains (Karkonosze Mountains) from the Carpathians or from Austrian parts of Alp? (ii) Do differences in genetic diversity exist within native and nonnative habitats? (iii) Does population structure reflect geographic distances?

This is the first study focusing on the genetic variability and population structure of the problematic weedy plant *R. alpinus*, which could provide new assessments of this species under a genetic context and produce valuable data for further control and management of plant invasions.

## 2 MATERIALS AND METHODS

### 2.1 Description of localities

**FIGURE 1:**
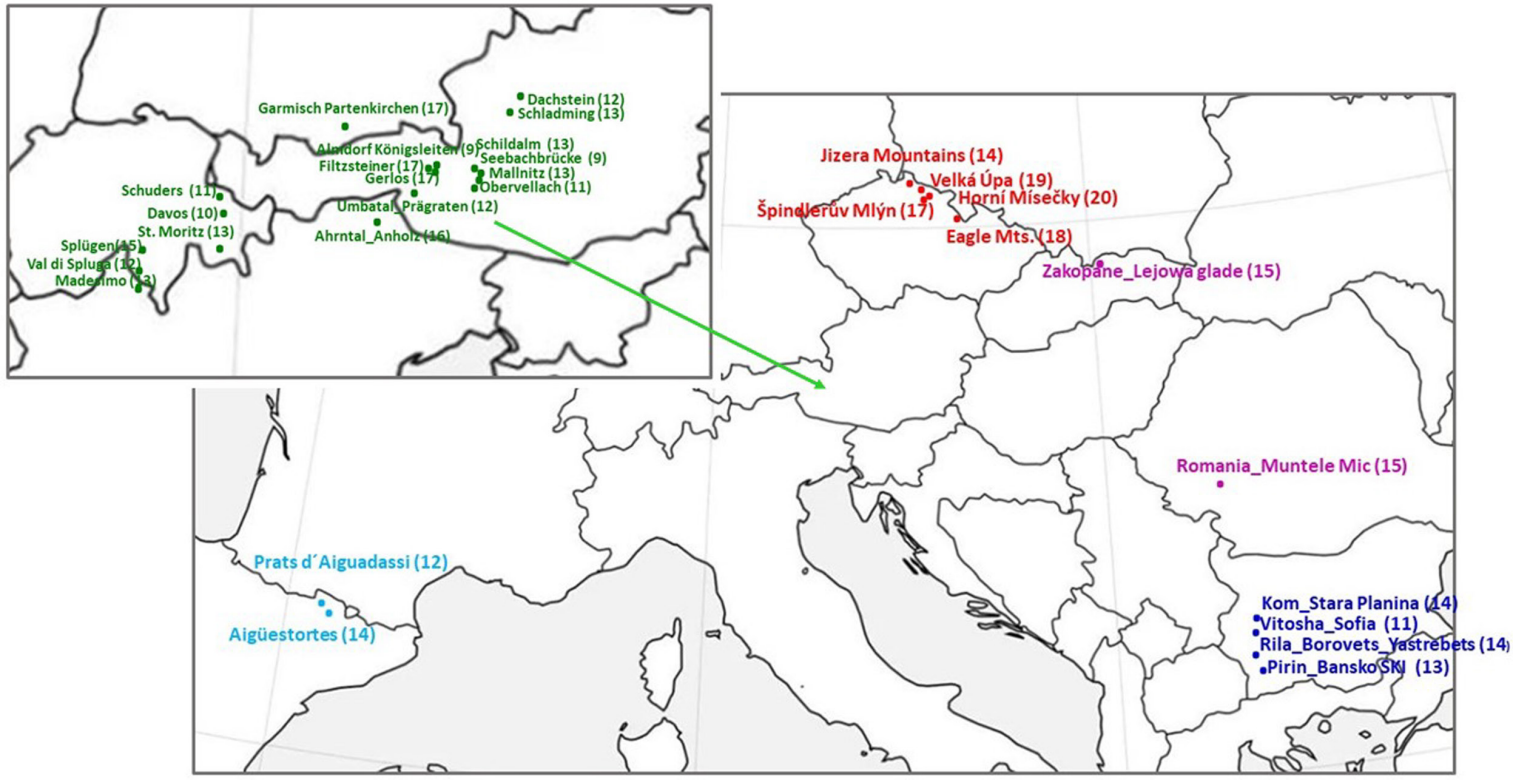
Geographical distribution of studied *Rumex alpinus* populations. The names of the populations correspond to Table 1, the numbers in brackets indicating the number of plants tested.

Between 2017 and 2020, plant samples of *R. alpinus* were collected from different locations in Europe (Table 1, Figure 1). According to Professor Klimeš research's focused on ancestors of the original alpine colonists and individual families, *R. alpinus* was collected in the exact places (Krkonoše) where the colonist's lived and *R. alpinus* probably occurred. Equally, individual plants of *R. alpinus* were collected in Tyrol and Styria in Austria. As control samples *R. alpinus* samples were collected from other mountain localities in the Alps (Lombardy and Graubünden) and the Pyrenees. Finally, to determine the true origin of *R. alpinus* were collected samples representing plant populations in the East and West Carpathians, and then in the mountainous regions of the Balkans. Populations from other mountainous regions of the Czech Republic (Jizera and Eagle Mountains) were collected for comparison and possible exclusion of other origins of *R. alpinus*.

**TABLE 1:** Rumex alpinus populations examined in this study.

No	Name of population	Latitude	Longitude	Mountains_Region_Country	Type of locality	Year
1	Garmisch Partenkirchen	47°28′16″ N	11°7′56″ E	Alps_Bavaria_Germany	Pasture	2019
2	Filtzsteiner	47°14′2″ N	12°7′39″ E	Alps_Tyrol_Austria	Next the road, pasture	2017
3	Gerlos	47°13′39″ N	12°3′24″ E	Alps_Tyrol_Austria	Pasture	2017
4	Almdorf Königsleiten	47°14′59″ N	12°7′8″ E	Alps_Tyrol_Austria	Next the road, pasture	2017
5	Seebachbrücke	47°0′28″ N	13°10′35″ E	Alps_Tyrol_Austria	Next the road, pasture	2017
6	Obervellach	46°56′9″ N	13°12′12″ E	Alps_Tyrol_Austria	Next the road, pasture	2017
7	Mallnitz	47°6′52″ N	12°30′14″ E	Alps_Tyrol_Austria	Next the road, pasture, banks	2017
8	Schildalm	47°6′30″ N	12°30′17″ E	Alps_Tyrol_Austria	Next the road, pasture	2017
9	Ahrntal_Anholz	46°53′1″ N	12°9′42″ E	Alps_Tyrol_Italy	Next the road, pasture, banks	2017
10	Umbaltal_Prägraten	47°0′59″ N	12°19′15″ E	Alps_Tyrol_Austria	Next the road, pasture	2017
11	Dachstein	47°27′1″ N	13°37′1″ E	Alps_Styria_Austria	Next the road, pasture	2017
12	Schladming	47°23′33″ N	13°45′30″ E	Alps_Styria_Austria	Pasture	2020
13	Madesimo	46°26′13″ N	9°21′27″ E	Alps_Lombardy_Italy	Road, chalets, pasture, ski slope	2017
14	Valle Spluga	46°28′7″ N	9°20′55″ E	Alps_Lombardy_Italy	Next the road, pasture	2017
15	Splügen	46°31′13″ N	9°19′50″ E	Alps_Lombardy_Switzerland	Next the road, pasture	2017
16	St. Moritz	46°28′47″ N	9°50′45″ E	Alps_Graubünden_Switzerland	Next the road	2017
17	Schuders	46°59′48″ N	9°43′31″ E	Alps_Graubünden_Switzerland	Around the village	2017
18	Davos	46°48′18″ N	9°51′53″ E	Alps_Graubünden_Switzerland	Pasture	2017
19	Horní Mísečky ●	50°43′4″ N	15°32′49″ E	Krkonoše Czech Republic	Road, chalets, ski slope	2017
20	Špindlerův Mlýn ●	50°44′39″ N	15°36′46″ E	Krkonoše Czech Republic	Grassland	2017
21	Velká Úpa ●	50°41′7″ N	15°46′49″ E	Krkonoše Czech Republic	Next the road, chalets, banks	2017
22	Jizera Mountains ●	50°48′42″ N	15°21′8″ E	Jizera Mts. Czech Republic	Next the road, chalets	2018
23	Eagle Mountains ●	50°19′34″ N	16°23′10″ E	Eagle Mts. Czech Republic	Next the road, chalets, ski slope	2018
24	Romania_Muntele Mic	45°22′25″ N	22°28′26″ E	Carpathians_Bihor Mountains_Romania	Next the road, chalets, ski slope	2018
25	Zakopane_Lejowa glade	49°15'47″ N	19°50′44″ E	Carpathians_Western Tatras_Poland	Pasture	2018
26	Kom_Stara Planina	43°11′15″ N	23°44′1″ E	Balkan Mts._Stara Planina_Bulgaria	Chalets, pasture	2018
27	Vitosha_Sofia	42°34′55″ N	23°14′31″ E	Balkan Mts._Vitosha_Bulgaria	Next the road, chalets, ski slope	2018
28	Rila_Borovets_Yastrebets	42°13′14″ N	23°34′33″ E	Balkan Mts._Rila_Bulgaria	Next the road, chalets, ski slope	2018
29	Pirin_Bansko SKI	41°46′54″ N	23°26′26″ E	Balkan Mts._Pirin_Bulgaria	Next the road, chalets, ski slope	2018
30	Prats d'Aiguadassi	42°34′6.8″ N	0°55′57″ E	Pyrennes_Catalan_Spain	Pasture	2017
31	Aigüestortes	42°34′8.4″ N	0°56′17″ E	Pyrennes_Catalan_Spain	Pasture	2017

*Note*: Populations of *Rumex alpinus* that are marked with a ● are nonnative, while the remaining populations are native plants.

Abbreviation: Mts, Stands for “Mountains”.

In total, 417 individual leaf samples were collected, representing 31 populations with 9–20 individual plants per population. The 329 plant samples were collected in the European Mountains, Alps, West and East Carpathians, Pyrenees, and Balkan Peninsula, which represent areas where *R. alpinus* is a native species.

In the Czech Mountains, represented by Eagle Mountains, Jizera Mountains, and Krkonoše (Giant) Mountains where *R. alpinus* is considered a nonnative/invasive plant (Kopecký, 1973; Kubát, 1990; Lokvenc, 1978; Pyšek et al., 2012; Št'astná et al., 2010), overall 88 plant samples were collected (Table 1 and Figure 1).

### 2.2 Population sampling and DNA extraction

At each locality, leave samples were collected in plants separated approximately 200 m apart to avoid collecting the same plant because *R. alpinus* also reproduces vegetatively and to ensure a representative sample of the population. Samples were dried on silica gel and stored in a collection at the Faculty of Environmental Sciences Czech University of Life Sciences Prague.

Total genomic DNA was extracted from silica gel‐dried leaves of *R. alpinus* in two repetitions using the protocol of Doyle and Doyle (1987) with a minor modification that involved the addition of 10 mg of polyvinylpyrrolidone (PVPP) (Carl Roth) and 5 μL of 10 mg/μL RNase A (Thermo Scientific) during the initial phase prior to incubation. The quality and yield of isolated DNA were assessed on a 0.8 % agarose gel in 1× TAE buffer. The concentration and quality of DNA were measured using a spectrophotometer UVS‐99/UVIS Drop (Avans Biotech). The extracted DNA samples diluted to the concentration of 20 ng/μL for subsequent analysis and stored at –20°C.

### 2.3 Microsatellite (SSR) analysis

Based on the test, 12 polymorphic primer pairs were selected from the 15 primer pairs according to Šurinová et al. (2018) and used (Table S1). DNA amplification was performed in 5 μL reactions consisting of 2.5 μL QIAGEN Multiplex PCR Master Mix; 0.125 μL of each M13‐labeled forward, reverse, and fluorolabelled (NED™, PET®, 6‐FAM™, VIC® – Table S1) M13 primers (10 μM each in initial volume); 20 ng of DNA dissolved in 0.5 μL TE buffer; and 1.625 μL dH_2_O. The PCR protocol was performed according to Schuelke (2000). PCR was performed using an Applied Biosystem Thermal Cycler (Applied Biosystems) as follows: an initial denaturation step at 95°C for 15 min followed by 25 cycles of denaturation (95°C for 20 s), annealing (59°C for 30 s), and extension (72°C for 20 s), followed by 10 cycles of denaturation (95°C for 30 s), annealing (53°C for 45 s), and extension (72°C for 45 s) and a final extension at 72°C for 15 min. During the first 25 cycles, specific PCR products are produced, and in the following 10 cycles, the fluorescent M13 tag is ligated to the M13 forward primer. The quality of PCR products has been verified on 2% agarose gels. Three multiplexes were built (Table S1).

Fragment analyses were performed using capillary electrophoresis in an ABI PRISM 3500 Genetic Analyzer automated sequencer (Applied Biosystems). Electropherograms were analyzed and scored using GeneMarker ver. 1.8 (SoftGenetics).

### 2.4 Statistical analysis

Analysis of the molecular variance test (AMOVA) with 1000 permutations was calculated in ARLEQUIN software ver. 3.5.2 (Excoffier & Lischer, 2010). The degree of genetic differentiation among populations was also evaluated using ARLEQUIN software using the distance matrix based on the fixation index (FST) generated by the program. Further, distance matrix based on geographical distances was calculated for *R. alpinus* populations within R program (R Core Team, 2020) version 4.0.3 using the routines in geosphere (Hijmans, 2021) library. These were subsequently logarithmically transformed and correlated with FST distance matrix using the Mantel test and 9999 permutations.

Nei's genetic distance was employed to obtain a UPGMA dendrogram after 1000 bootstrap samplings in TFPGA software (Miller, 1997).

The diversity indices for each population included the percentage of polymorphic loci, the average diversity of the loci using Nei's unbiased gene diversity *ĥ* (Nei, 1973), and the Shannon information index (Lewontin, 1972; Shannon & Weaver, 1949) were calculated using the POPGENE, version 1.32 (Yeh et al., 1999).

To assess the Hardy–Weinberg equilibrium, we used the ARLEQUIN software ver. 3.5.2 (Excoffier & Lischer, 2010). Was conducted an exact test using a Markov chain with a forecasted chain length of 1,000,000 and 100,000 dememorization steps (Guo & Thompson, 1992). Deviation from HWE was assessed at a significance level of *p* < .05. The results were interpreted according to established guidelines (Levene, 1949).

Another approach to studying population structure analysis is based on Bayesian statistics STRUCTURE, version 2.3.4 (Pritchard et al., 2000) was used to determine the genetic architecture of the *R. alpinus* populations. Ten independent runs of 1–20 groups (*K* = 1–20) were performed using the locprior model with admixture and correlated allele frequency (Falush et al., 2003; Hubisz et al., 2009) with the recommended 2,00,000 Markov chain iterations after a burn‐in period of 1,00,000 iterations. The optimal value of K was estimated based on ln (K) and on the *ΔK* calculation, which considers the rate of change in the ln P (D) values among successive K runs to account for patterns of dispersal that are not homogeneous among populations (Evanno et al., 2005). The number (K) of clusters into which the sample data (X) were fitted with posterior probability Pr (X|K) was estimated using the same model with 1,000,000 Markov chain iterations after a burn‐in period of 1,00,000 iterations (Evanno et al., 2005).

An exact test for population differentiation was calculated using the Tools for Population Genetic Analyses (TFPGA; version 1.3; Miller, 1997) with 1,00,000 recommended permutation steps.

To identify potential bottleneck events in the populations under investigation, we employed BOTTLENECK 1.2.02 software (Cornuet & Luikart, 1996; Piry et al., 1999) and heterozygosity excess resulting from population reduction was examined. We utilized three models of mutational equilibrium: the infinite allele model (IAM), the stepwise mutation model (SMM), and the two‐phase mutation model (TPM), with the latter being the most appropriate for microsatellites. For the TPM, we employed the default settings, which assumed that 70% of mutations occur in a single step, with a variance of 30 among multiple steps. The significance of these models was assessed using a one‐tailed Wilcoxon rank test, which is suitable for datasets analysis with less than 20 microsatellite loci (Piry et al., 1999). A population was deemed to have experienced a bottleneck event only if all three models produced significant results (*p*‐value ≤.05).

## 3 RESULTS

The number of alleles in different loci is presented in Figure S1, and the sum of all alleles for each population is in Table 2. In the 417 analyzed individuals, 146 alleles were identified for the 12 microsatellite loci (Table S3) with an average of 9.93 polymorphic loci (Table 2). The mean of alleles per locus for all populations was 3 and ranged from the population of Eagle Mountains (1.5) to Carpathian Bihor Mountains population (5.2), where the highest number of nine alleles per locus was identified (Figure S1). While the percentage of polymorphic loci was the highest in the Zakopane Lejowa glade population (100%) the lowest number was found in the Eagle Mountains (42%) (Table 2). A total of 340 multilocus genotypes from 417 individuals of *R. alpinus* were identified. Some populations consisted partly or totally from identical clones, especially the Eagle Mountains population (Table S3).

**TABLE 2:** Characteristics of 31 local populations of *Rumex alpinus* and their diversity evaluation based on 12 SSR loci analyses.

No	Name of population	*n*	Mountains	Region_Country	I	St. dev.	*ĥ*	St. dev.	*P* (%)	Σ_poly‐morphic loci	Σ‐alleles	FST /Mts.	Nm*/Mts.
1	Garmisch Partenkirchen	17	Alps	Bavaria_Germany	0.69	0.48	0.38	0.26	83	10	37	0.2	1.03
2	Filtzsteiner	12	Alps	Tyrol_Austria	0.79	0.54	0.43	0.27	83	10	37		
3	Gerlos	9	Alps	Tyrol_Austria	0.79	0.49	0.45	0.24	92	11	37		
4	Almdorf Königsleiten	9	Alps	Tyrol_Austria	0.79	0.49	0.44	0.24	92	11	38		
5	Seebachbrücke	9	Alps	Tyrol_Austria	0.77	0.52	0.43	0.27	83	10	35		
6	Obervellach	11	Alps	Tyrol_Austria	0.84	0.49	0.47	0.26	92	11	40		
7	Mallnitz	13	Alps	Tyrol_Austria	0.84	0.56	0.46	0.29	92	11	40		
8	Schildalm	13	Alps	Tyrol_Austria	0.76	0.43	0.44	0.24	92	11	34		
9	Ahrntal_Anholz	16	Alps	Tyrol_Italy	0.68	0.63	0.35	0.30	92	11	38		
10	Umbaltal_Prägraten	12	Alps	Tyrol_Austria	0.81	0.61	0.42	0.28	92	11	43		
11	Dachstein	12	Alps	Styria_Austria	0.74	0.44	0.43	0.25	92	11	36		
12	Schladming	13	Alps	Styria_Austria	0.75	0.36	0.44	0.20	92	11	35		
13	Madesimo	13	Alps	Lombardy_Italy	0.83	0.53	0.46	0.28	83	10	39		
14	Valle Spluga	12	Alps	Lombardy_Italy	0.92	0.61	0.49	0.28	83	10	43		
15	Splügen	15	Alps	Lombardy_Switzerland	0.97	0.57	0.52	0.25	92	11	47		
16	Schuders	11	Alps	Graubünden_Switzerland	0.97	0.52	0.52	0.25	92	11	45		
17	St. Moritz	13	Alps	Graubünden_Switzerland	0.80	0.52	0.44	0.28	92	11	37		
18	Davos	10	Alps	Graubünden_Switzerland	0.87	0.58	0.46	0.30	83	10	43		
19	Horní Mísečky ●	20	Krkonoše	Krkonoše Czech Republic	0.58	0.38	0.38	0.24	75	9	24	0.22	0.88
20	Špindlerův Mýn ●	17	Krkonoše	Krkonoše Czech Republic	0.47	0.37	0.30	0.25	75	9	24		
21	Velká Úpa ●	19	Krkonoše	Krkonoše Czech Republic	0.59	0.40	0.39	0.25	75	9	24		
22	Jizera Mountains ●	14	Jizera Mts.	Jizera Mts. Czech Republic	0.44	0.44	0.28	0.28	58	7	22		
23	Eagle Mountains ●	18	Eagle Mts.	Eagle Mts. Czech Republic	0.32	0.36	0.23	0.26	42	5	16		
24	Romania_Muntele Mic	16	Carpathians	Bihor Mountains_Romania	1.11	0.73	0.52	0.32	92	11	62	0.05	5.05
25	Zakopane_Lejowa glade	15	Carpathians	Western Tatras_Poland	0.99	0.58	0.50	0.30	100	12	50		
26	Kom_Stara Planina	14	Balkan	Stara Planina_Bulgaria	0.99	0.70	0.49	0.32	75	9	51	0.12	1.75
27	Vitosha_Sofia	11	Balkan	Vitosha_Bulgaria	0.92	0.76	0.46	0.35	67	8	45		
28	Rila_BorovetsYastrebets	14	Balkan	Pirin_Bulgaria	1.05	0.81	0.50	0.36	75	9	55		
29	Pirin_Bansko SKI	13	Balkan	Rila_Bulgaria	0.73	0.71	0.37	0.32	75	9	42		
31	Prats d'Aiguadassi	14	Pyrennes	Catalan_Spain	0.65	0.59	0.35	0.30	75	9	35	0.05	5.29
30	Aigüestortes	12	Pyrennes	Catalan_Spain	0.82	0.63	0.42	0.31	92	11	42		
Mean					0.78	0.54	0.43	0.28	82.8	9.93	38.5	0.13	2.80

*Note*: Nm* = Gene flow estimated from FST = 0.25 (1 – FST)/FST. Populations of *Rumex alpinus* that are marked with a ● are nonnative, while the remaining populations are native plants.

Abbreviations: FST/Mts. the fixation index per the Mountains; *ĥ*, observed heterozygosity; I, Shannon‐Wiener Diversity Index; Nm*/Mts, the effective number of migrants per the Mountains (A higher Nm* indicates higher gene flow between populations); St. Dev. P (%), the percentage of polymorphic loci; St. Dev, standard deviation; Σ_polymorphic loci, the total number of loci in a population that has more than one allele; Σ‐alleles, the total number of different alleles observed across all loci in a population.

Nei's average gene diversity values ranged from 0.23 in the population of the Eagle Mountains to 0.52 in the population of Splügen and Schuders (Table 2). The overall gene diversity for all populations was 0.43. The Shannon diversity index (I) was the lowest in the population from the Eagle Mountains (*I* = .32), and the highest (*I* = 1.11) was in the population from Romania Muntele Mic (Table 2). The overall mean value of I was 0.78 when all populations were included (Table 2). The level of population genetic differentiation (FST) ranged from 0.05 to 0.22, with an average of 0.12. While, the gene flow (Nm) level was found in the Czech Mountains (0.88), populations from Pyrenees demonstrated the highest level (5.29), with an average of 2.80 (Table 2).

The level of genic diversity of 5 mountains was lowest in *R. alpinus* populations from Czech Mountains (^*ĥ* = 0.39; *I* = .65), followed Pyrenees Mountains (^*ĥ* = 0.39; *I* = .79); Balkan Mountains (^*ĥ* = 0.52; *I* = 1.17), Carpathian Mountains (^*ĥ* = 0.54; *I* = 1.17), and the highest level of genic diversity was found in populations from the Alps (^*ĥ* = 0.56; *I* = 1.16) (Table 3).

**TABLE 3:** Analysis of genic variation statistics for all loci according to Nei (1987) developed for all five mountain populations.

Name of mountains	I	St. dev.	*ĥ*	St. dev.	P (%)	Σ_polym.Loci	Σ‐alleles
Alps	1.16	0.70	0.55	0.29	100	12	6
Czech Mountains	0.65	0.47	0.39	0.26	75	9	3
Carpathians	1.17	0.69	0.54	0.30	100	12	6
Balkans	1.17	0.88	0.52	0.36	75	9	6
Pyrenees	0.79	0.62	0.39	0.31	100	12	4

Abbreviations: *ĥ*, observed heterozygosity; I, Shannon‐Wiener Diversity Index; P (%), the percentage of polymorphic loci; St. Dev, standard deviation; Σ_polymorphic loci, the total number of loci in a population that has more than one allele; Σ‐alleles, the total number of different alleles observed across all loci in a population.

Genetic variability was measured as the amount of observed or expected heterozygosity, presented in Table S2. The mean level of Hardy–Weinberg equilibrium for Alpine, Carpathians, and Balkan populations showed that the observed heterozygosity values were not significant, indicating that the populations were in Hardy–Weinberg equilibrium. However, the level of Hardy–Weinberg equilibrium for Czech Mountain populations showed that the observed heterozygosity values were significantly lower than the expected ones for more than seven loci (*p*‐value <.01), indicating that the population was not in Hardy–Weinberg equilibrium.

Based on the results, all Alpine, Balkan, and Czech Mountain populations had at least one monomorphic locus, meaning that every individual in the population had the same homozygous genotype at that locus. Specifically, in the Eagle Mountains population, loci 1, 2, 3, 4, and 10 were monomorphic. In contrast, loci 5, 7, 8, 9, and 12 exhibited an observed heterozygosity (HO) of 1000 and an expected heterozygosity (HE) of 0.514, suggesting that the population is highly inbred or clonal, as all individuals have the same genotype at the locus. On the other hand, the results for the Carpathian population Zakopane_Lejowa glade did not record any monomorphic locus. However, significant results on some loci suggest that the population might be experiencing some form of selection (Table S2).

The results of the AMOVA for all native and non‐native populations (Table 4) indicated that a significant proportion of the genetic variation (26.8%) occurs both among groups (mountains) of populations and mainly within populations (60.2%). A smaller proportion (13.0%) was found among populations within groups. Like to the previous results, AMOVA results for the native populations (Table 5) suggested that a significant proportion of the genetic variation is found among groups (mountains) of populations (33.6%) and primarily within populations (54.2%). In contrast, a smaller proportion (12.2%) was found among populations within groups. Similar to the previous two AMOVA results for nonnative populations (Table 6) suggested that a significant proportion (71.7%) of the genetic variation can be explained by differences within populations, while a smaller 28.1% of differences can be explained by the variability among groups (mountains). As only 0.2% of the genetic variation was found among populations within groups, indicating only minor genetic differences among the populations within each group (Table 6).

**TABLE 4:** Analysis of molecular variance (AMOVA) results with the 31 native and nonnative populations of *Rumex alpinus*.

Source of variation	d.f.	Sum of squares	Variance of components	Percentage of variation	Fixation indices	*p*‐Value
Among groups	4	553	0.907	26.8	0.398	.001
Among populations within groups	26	355	0.439	13.0	0.177	.001
Within populations	803	1635	2.036	60.2	0.268	.001
Total	833	2543	3.382			

*Note*: The populations were divided into groups according to mountain communities (the Alps, Czech Mountains, Carpathians, Balkans, and Pyrenees).

**TABLE 5:** Analysis of molecular variance (AMOVA) results with the 26 native populations of *Rumex alpinus*.

Source of variation	d.f.	Sum of squares	Variance of components	Percentage of variation	Fixation indices	*p*‐Value
Among groups	3	389	1.045	33.6	0.458	.001
Among populations within groups	22	246	0.381	12.2	0.184	.001
Within populations	632	1067	1.689	54.2	0.336	.001
Total	657	1703	3.115			

*Note*: The populations were divided into groups according to mountain communities (the Alps, Carpathians, Balkans, and Pyrenees).

**TABLE 6:** Analysis of molecular variance (AMOVA) results with the 5 nonnative populations of *Rumex alpinus*.

Source of variation	d.f.	Sum of squares	Variance of components	Percentage of variation	Fixation indices	*p*‐Value
Among groups	2	71	0.717	28.1	0.283	.001
Among populations within groups	2	4	0.005	0.2	0.003	.148 ± .010
Within populations	171	313	1.831	71.7	0.281	.106 ± .008
Total	175	388	2.553			

*Note*: The populations were divided into three groups according to mountain communities (the Krkonoše Mountains, Jizera Mountains; and Eagle Mountains).

**FIGURE 2:**
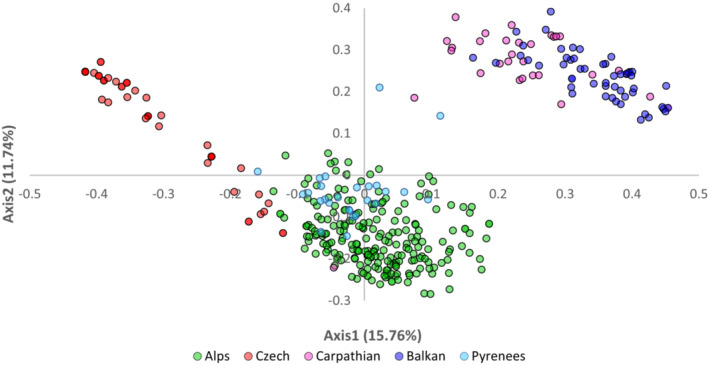
The plot of PCoA analyzed populations of *Rumex alpinus* from the Alps, Czech, Carpathians, Balkans, and Pyrenees Mountains.

The two first axes of the PCoA analysis (Figure 2) of SSR data explained 15.76% and 11.74% of the total variance, respectively, and separated individuals into three main groups (Figure 2). The largest group in the lower quadrant of the plot comprised the majority of all sampled individuals from the Alps (Bavaria, Tyrol, Styria, Lombardy) and Pyrenees. All individuals (except the one) of Carpathians and Balkans populations formed the second group, separated from the main group along the first axis, whereas the third group containing all the populations of the Czech Mountains was separated along the first and second axes.

**FIGURE 3:**
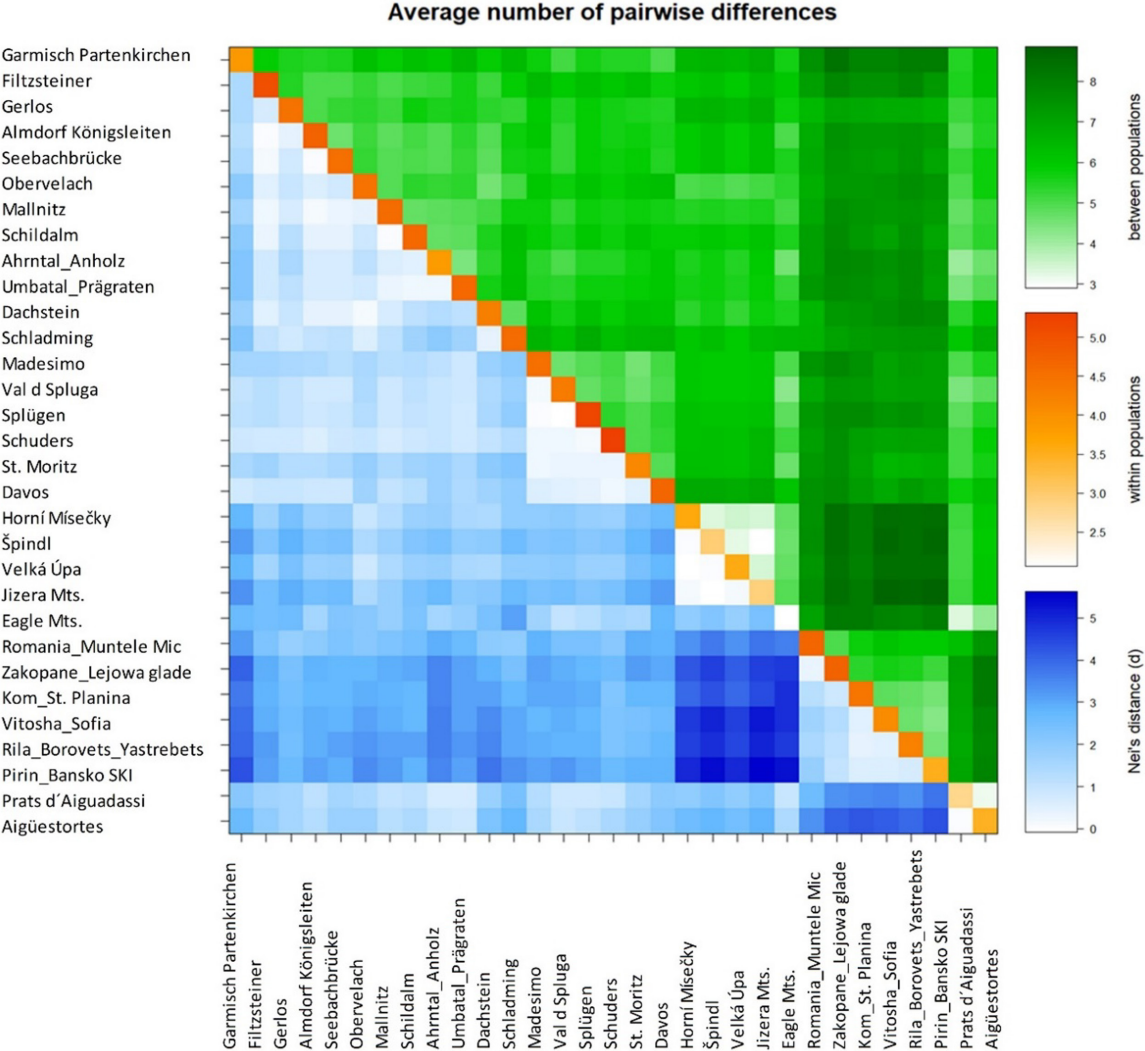
The correlation between pairwise FST values and logarithm of pairwise geographical distance between populations of *Rumex alpinus*.

Pair differences and the degree of variability between *R. alpinus* populations were quite high. The total value for the (FST) between populations was 0.40. The differences and degree of variability between the populations were clear, as shown in Figure 3.

**FIGURE 4:**
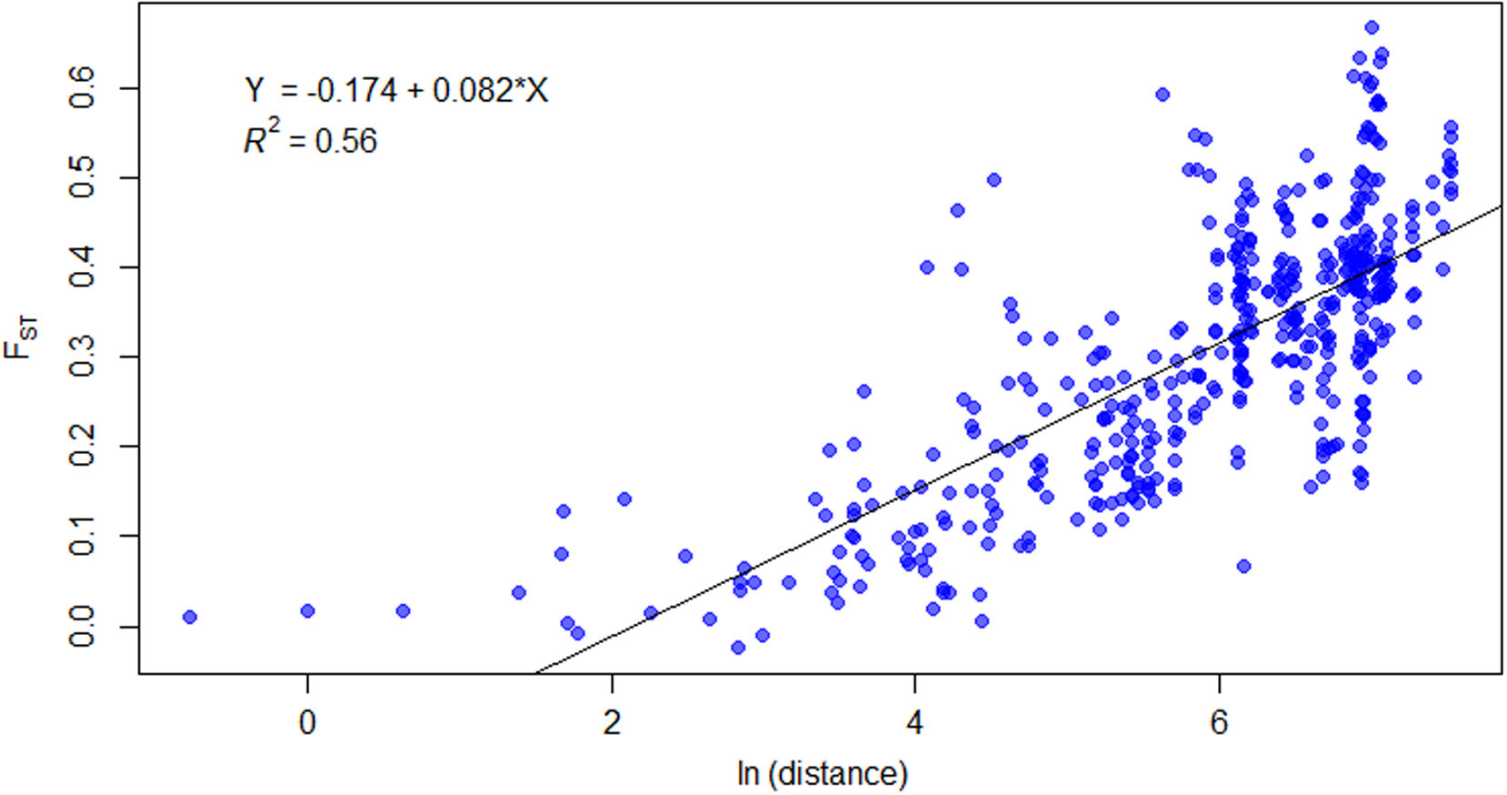
Correlation between paired FST values and logos by the rhythm of paired geographical distances between *Rumex alpinus* populations (*r* = .65 *p* ˂ .001) calculated by Mantel test and expressed by the linear regression model, where Y is the fixation index FST and X is the logarithm of distances in expressed in km.

The Mantel test revealed a significant positive correlation between geographical and genetic distances (*r* = .65; *p* < .01) across all the localities. The linear regression model was identified as a representation of the relationship between geographical and genetic distances (Figure 4).

**FIGURE 5:**
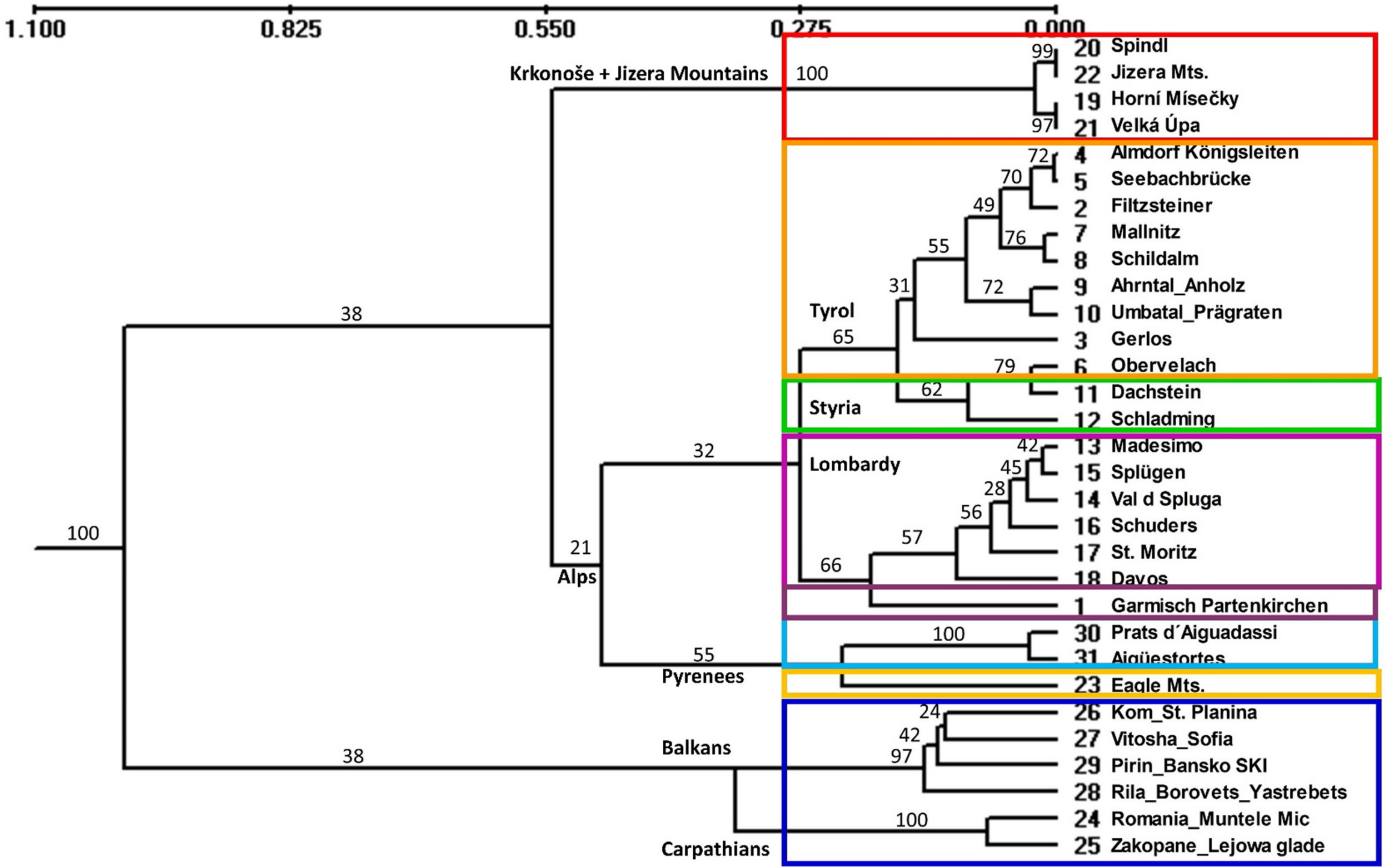
UPGMA dendrogram was constructed based on 12 molecular microsatellite markers using Nei's genetic distances (Nei, 1978) for 31 populations of *Rumex alpinus* in the European Mountains. Bootstrap values of the consensus tree are given in branches. The length of the branches is proportional to the genetic distance. Numbers and the name of populations see in Tables 1, 2. The colors correspond to the colors of the mountains in the Figure 6a.

The UPGMA dendrogram based on Nei's genetic distance matrix (Figure 5) represented two main groups. The first group was composed of two clusters containing populations from the Balkan and Carpathian Mountains. The second group was further divided into three clusters, one of which contained populations from the Czech Mountains (Krkonoše and Jizera Mountains), the second was formed by populations from the Pyrenees and a population from the Eagle Mountains. The third most comprehensive alpine cluster was divided into two subgroups, one contained populations from Lombardy and Garmisch Partenkirchen, while the populations from Styria, and the Tyrolean Alps formed the second subgroup Tables 6 and 7.

**TABLE 7:** Bottleneck testing using one tailed Wilcoxon rank test for IAM, TPM, and SMM model. Populations where the probability level for all three models where under significance threshold (*p* < .05) are highlighted by yellow text.

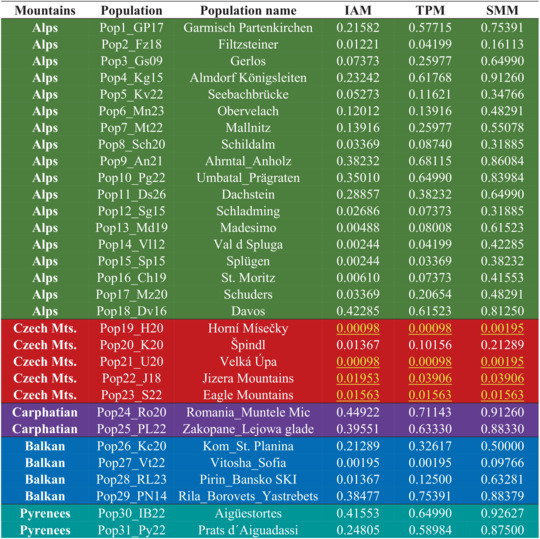

**FIGURE 6:**
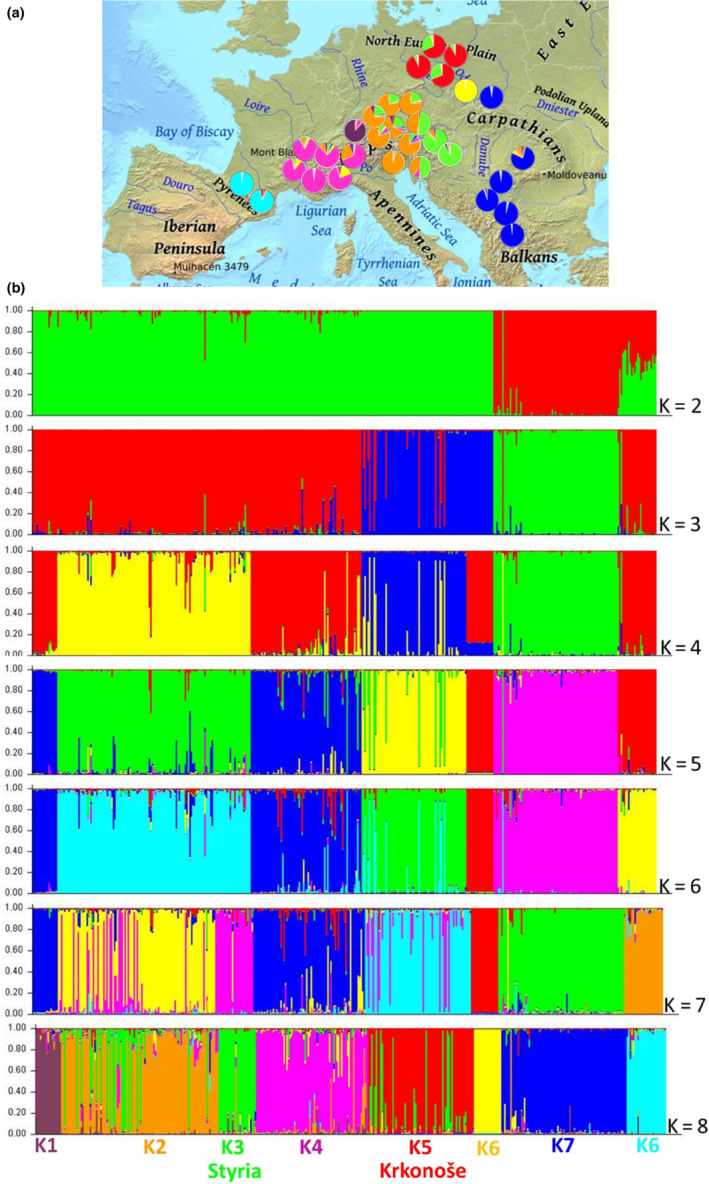
(a) Sample localities of *Rumex alpinus* populations with pie charts describing the proportions of individuals classified into one of the eight clusters defined using the Bayesian approach (Pritchard et al., 2000). Color coded pie charts indicate the proportion of individuals within each population that corresponds to a particular STRUCTURE identified genetic deme (coresponding with Figure 6b K = 8 for details). (b) Bayesian model‐based clustering of analyzed accessions. Bar plots show the membership coefficient estimate (Q) for each accession for the inferred clusters with maximum log‐likelihood probability. Bar colors and lengths represent inferred clusters and Q, respectively, identified by STRUCTURE for K = 2, 3, 4, 5, 6, 7, and 8. As members from PCoA clusters o were rather heterogeneous reasonable division was found by STRUCTURE for K = 8 (detail: K1 Alps Bavaria, K2 Alps Tyrol, K3 Alps Styria, K4 Alps Lombardy, K5 Czech Mountains, K6 Eagle Mountains, K7 Carpathians and Balkans, K8 Pyrenees). Individual colors in do not represent the same clusters for technical reasons given by the software.

A noticeable genetic structure of *R. alpinus* populations was found. Although the optimal value of K = 8 was identified, the subsequent analysis results with K from two to eight were displayed (Figure 6b). When K = 2, two genetic populations representing Alpine and Balkans groups of local populations were identified. Whereas, the results for K = 3, the Czech Mountains' local populations (dark blue) were distinguished from the Alpine ones. When K = 4, Tyrolean local population (yellow) was separated from Alpine Mountains local populations, and Eagle Mountains local populations were colored identically to the Alps populations (Figure 6b). While the result for K = 5, are additionally identically colored, the Lombardy populations with Bavaria local populations (dark blue) and the Pyrenees with the Eagle Mountains local populations (red). When K = 6, the Pyrenees were distinguished as a separate genetic population In the case of K = 7, the alpine regions were divided into more distinguished groups of local populations, where Styria is seen as colored pink (Figure 6b). As in the previous results (K = 4, 5, 6), the local Bavarian populations formed genetically the same population as the Lombard ones (Figure 6b).

**FIGURE 7:**
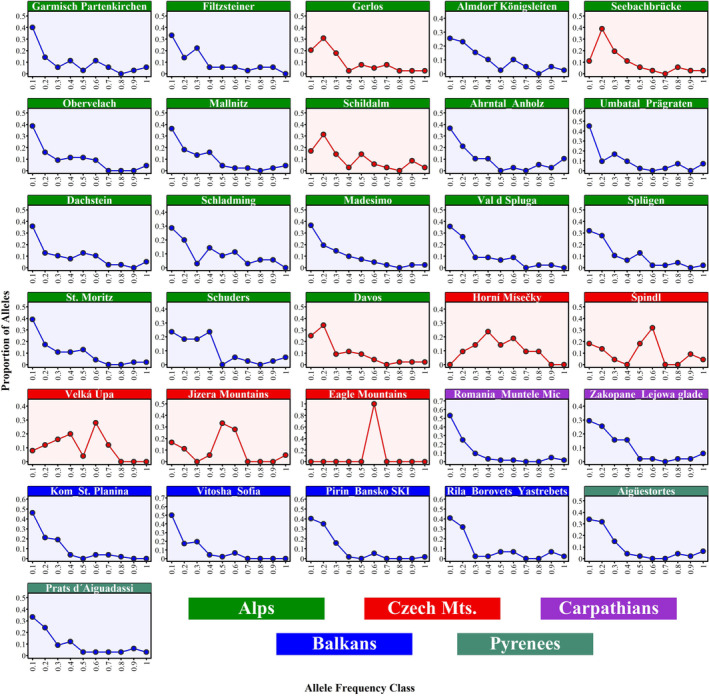
Mode‐shift plot indicating the occurrence of a recent bottleneck event. The figure displays the distribution of allele frequencies for 12 microsatellite loci in 31 populations of *Rumex alpinus* collected from the Alps (green), Czech Republic (red), Carpathians (purple), Balkans (blue), and Pyrenees (aqua). The x‐axis shows the allele frequencies grouped into categories of 0.1, while the y‐axis indicates the percentage of alleles in each frequency category. The red lines on the plot denote populations that exhibit a mode shift in the frequency distribution, which is a hallmark of the bottleneck effect.

Moreover, the Evanno method showed that the best number of populations is K = 8 and is most similar to the result of UPGMA analysis. The *ΔK* value identified eight clusters K1–K8 among *R*. alpinus populations (see Figure 6b—k = 8). Expected heterozygosity between individuals within the same cluster ranged from 0.252 (K4), which consisted of populations Alps_Lombardy, to 0.571 (K3), which presented populations from Alps_Styria, with an average of 0.406. FST values ranged from 0.234 (K3) to 0.792 (K4), with an average of 0.479. The FST value of cluster K5, which consisted of Krkonoše and Jizera Mountains, was 0.303. The mean value of *α* was 0.03, indicating that most *R*. alpinus genotypes were not genetically admixed (Falush et al., 2003). The representation of individual samples of *R. alpinus* in eight genetic populations for each local population can be seen in the map (Figure 6a) corresponding with K = 8 (Figure 6b). While genetic population K1 is typical for Alps_Bavaria *R. alpinus* local populations (88%), K2 for Alps_Tyrol and Alps_Styria local populations (52%, 31%), K3 for Styria local populations (89%), and K4 consists for Alps_Lombardy local populations (89%), genetic populations K5and K6 are predominant for Czech Mountain local populations (81%, 99%, resp.). Cluster K8 consisted exclusively of Pyrenees populations (93%). The distribution of allele frequency (Figure 7) suggested the occurrence of a bottleneck effect in four Alpine populations (Gerlos, Seebachbürcke, Schildalm, and Davos) and all five populations from the Czech Republic. However, upon further analysis using the one‐tailed Wilcoxon rank test, only four populations of Czech origin produced significant results for all three mutation models (Table 7). This confirmed that populations from Horní Mísečky, Velká Úpa, Jizera Mountains, and Eagle Mountains experienced bottleneck events.

## 4 DISCUSSION

The possibility of comparing the genetic variation of *R. alpinus* with other Rumex species is limited due to the low number of species examined for SSR marker diversity. Indeed, the physiology and ecology of *R. alpinus* have been studied (Hujerová et al., 2013; Jungová et al., 2022; Řičařová, 2011) much more than genetic variability, with the only published genetic work being on *Rumex bucephalophorus* subsp. canariensis (Viruel et al., 2015). Unfortunately, *Rumex bucephalophorus* is an annual Mediterranean plant.

This is the first study on genetic variability and population structure of the weedy plant *R. alpinus*, which could provide new assessments of this species in a genetic context and produce valuable data for further control management of plant invasions (Le Roux & Wieczorek, 2008). Molecular markers helped elucidate the introduction history and contributed to the explanation of genetic variation in invaders, as in other studies (Bímová et al., 2003; DeWalt et al., 2011; Matesanz et al., 2014; Šurinová et al., 2018). The 12 SSR loci used in this study were significantly polymorphic and useful for differentiation among the *R. alpinus* populations studied (Šurinová et al., 2018).

The population structure of *R. alpinus* in the introduced range is consistent with the random establishment of genotypes in different localities developed by human dispersal. Probably for various socio‐economic reasons, *R. alpinus* extended in many Central European mountains (Stachurska‐Swakoń, 2008; Delimat & Kiełtyk, 2019) in abandoned or inappropriately managed mountain pastures (Bohner, 2005; Rehder, 1982), including the Krkonoše Mountains (Červenková & Münzbergová, 2009; Pyšek et al., 2012; Št'astná et al., 2010). This is evident from the pairwise differences, which showed that population structure reflected a pattern of isolation by distance associated with human dispersal in the past (Kopecký, 1973; Kubát, 1990; Lokvenc, 1978; Maude & Moe, 2005; Št'astná et al., 2010; Vasas et al., 2015). The correlation analysis relevated a moderate to a strong positive correlation between paired FST values and the differences in geographical distances between *R. alpinus* populations. This suggests no gene flow between populations. Some degrees of gene flow were caused most likely by repeated human impact (Matesanz et al., 2014), which was in accordance with genetic admixture detected in Structure analysis. The anthropogenic situation has probably promoted the movement of propagules across the introduced range (Matesanz et al., 2014), and individual populations have been founded by relatively few individuals (Dlugosch & Parker, 2008; Genton et al., 2005). The results of the Hardy–Weinberg equilibrium analysis showed that most Alpine and Balkan populations are in equilibrium and gene flow is occurring here. However, the situation is different in the Czech populations, especially in the Eagle Mountains population, where only one clone was identified. Nevertheless, the source of those individuals bears a relationship to the geographical or ecological distance from the site where they were established, which is seen from other analyses. The principal coordinate analysis PCoA showed that two groups, one of the Czech Mountains populations and the Carpathians and the Balkans Mountain populations, separated from the parental Alpine population over the centuries. Based on these results, it is unlikely that *R. alpinus* was introduced from the Carpathians to the Krkonoše Mountains. Individuals from the Carpathian Mountains populations were associated with the Balkan populations in one cluster, as the Structure results indicated gene flow between them. On the other hand, from the PCoA and in more detail Structure analyses, it was also evident that the Czech Mountains cluster was more clearly separated from the alpine cluster, suggesting that *R. alpinus* could be a native plant of the Krkonoše Mountains.

However, based on historical sources (Hendrych, 2001; Lokvenc, 2007), it is known that botanist Caspar Schwenckfelt (1607) in his botanic book Scite aus dem botanischen Teil des Buches did not mention the very conspicuous herb *R. alpinus*, and such a large plant cannot be overlooked (Hendrych, 2001). The first historical record that supported the allochthonous origin of *R. alpinus*, but with a different idea of its introduction, is mentioned only by Wimmer in 1844, who described the findings of these plants in mountain huts (Hendrych, 2001). However, the results of Structure analyses showed that the Krkonoše populations corresponded to the average posterior likelihood that the individual was assigned to the cluster with populations from Styria. Based on the results of these analyses, supported by the historical research of Professor Klimeš, it is evident that *R. alpinus* was introduced to the Krkonoše Mountains mainly by colonists from Styria, whether they were lumberjacks (Klimeš, 2011) or raftsmen (Smrčka, 2016).

Nevertheless, the less distinct molecular difference was found between *R. alpinus* from the Czech Mountains and *R. alpinus* in the Alps, which was probably caused due to the bottleneck effect in all of Krkonoše populations except Špindl (DeWalt et al., 2011; Durka et al., 2005; Genton et al., 2005; Hardesty et al., 2012). In the localities, there may have been a drastic reduction in the number of individuals, inbreeding, and thus allele loss as the population settled into the new territory (Wright, 1931; Maron et al., 2004; Keshavarzi & Mosaferi, 2019).

On the other hand, a founder effect could have occurred there when a new territory was settled, and an individual with a unique allele was introduced (Bossdorf et al., 2005; Matesanz et al., 2014; Oduor et al., 2016). There could have also been an accumulation of mutations or hybridization with the related species Rumex species (Kubát, 1990; Rechinger, 1957; Št'astná et al., 2010; Stehlik, 2002).

Amova results of *R. alpinus* revealed high variability within populations rather than between populations themselves (Mosaferi et al., 2015; Sheidai et al., 2016). This fact was subsequently confirmed by the results of the Hardy–Weinberg equilibrium, especially in Alpine populations. Similarly, higher genetic polymorphism in native localities was confirmed in other studies (Chen et al., 2009; Keshavarzi & Mosaferi, 2019; Leišová‐Svobodová et al., 2018; Rollins et al., 2013). It is consistent with the statement that in natural habitats, plants such as *R. alpinus* reproduced by generative proliferation (Červenková & Münzbergová, 2009; Klimeš et al., 1993, 1997), leading to subsequent higher genetic variability (Briggs & Walters, 2016). This is mainly due to their natural enemies, such as insect pests or fungal diseases, and competition (Bímová et al., 2003; Barrett, 2015; Kaljund et al., 2013; Mandák et al., 2005). While in nonnative sites without native enemies, plants prefer clonal reproduction (Bímová et al., 2003; Klimeš et al., 1997), even at the cost of lower genetic variability (Briggs & Walters, 2016; Dlugosch & Parker, 2008).

In addition, the Hardy–Weinberg equilibrium results showed the Czech Mountains, especially in the Eagle Mountains, no genetic variation at several loci. It is assumed that the population in Eagle Mountains is highly inbred (Šurinová et al., 2018), and the population consisted only of clones (reproduced via rhizomes in falanga habit Klimeš et al., 1993), contributing to the opinion that this species is nonoriginal and is, therefore, a secondary occurrence (Hollingsworth & Bailey, 2000). Obtained results confirmed the previously published hypothesis that *R. alpinus* was spread mainly by human distribution from small sources of populations in the Middle Ages (Kopecký, 1973; Lokvenc, 2007).

Additionally, the study found that the genetic variability of *R. alpinus* populations in the Alps, Carpathians, and Pyrenees—where it is considered to be native (Bohner, 2005; Delimat & Kiełtyk, 2019; Raycheva & Dimitrova, 2007; Stachurska‐Swakoń, 2008)—was higher compared to the nonnative populations in the Czech Mountains. These findings, as reported by Amsellem et al. (2000), support the conclusion that *R. alpinus* is indeed a nonnative species in the Czech Mountains.

The variation in the Balkan and Carpathian populations suggests that a single introduction from one native‐range population is unlikely. In contrast, the great diversity and the high interpopulation differentiation found in Carpathian populations indicated more native sources.

## 5 CONCLUSION

The genetic variability and population structure of the weedy plant *R. alpinus* were studied to provide new insights into this species under a genetic context and to generate valuable data to control plant invasions. The study employed 12 SSR loci that were significantly polymorphic and helpful for distinguishing among the populations of *R. alpinus* studied. *R. alpinus* population structure in the introduced range suggests that the genotypes were randomly established in different localities through human dispersal, with no gene flow between populations. Hardy–Weinberg equilibrium results indicated that most native populations of *R. alpinus* are in equilibrium, and gene flow is occurring. The source of the individuals is related to the geographical or ecological distance from the site where they were settled. It seems that *R. alpinus* was introduced to the Krkonoše Mountains mainly by colonists from Styria. Molecular differences between *R. alpinus* in the Czech Republic and *R. alpinus* in the Alps are probably caused by a founder effect and bottleneck effect. Native *R. alpinus* populations showed higher genetic polymorphism, mainly due to their natural enemies and competition. In nonnative sites without native enemies, plants prefer clonal reproduction, especially the Eagle Mountains, which showed no genetic variation at several loci. Overall, the low genetic variability in the Czech populations indicated that major expansion of this invasive plant species in nonnative habitats is unlikely, and appropriate management can help maintain it in the future.

## AUTHOR CONTRIBUTIONS

**Michaela Jungová:** Conceptualization (equal); data curation (equal); formal analysis (equal); methodology (equal); project administration (equal); resources (equal); writing – original draft (equal). **Vladimíra Müllerová Jurasová:** Conceptualization (equal); formal analysis (equal); methodology (equal); resources (equal); validation (equal); writing – review and editing (equal). **Petra Hlasna Čepková:** Data curation (equal); investigation (equal); methodology (equal); resources (equal); supervision (equal); writing – review and editing (equal). **Leona Leisova Svobodová:** Data curation (equal); formal analysis (equal); software (equal); writing – review and editing (equal). **Pavel Svoboda:** Data curation (equal); software (equal); writing – original draft (equal). **Michal Hejcman:** Conceptualization (equal); investigation (equal); project administration (equal); writing – review and editing (equal).

## CONFLICT OF INTEREST STATEMENT

None declared.

## FUNDING INFORMATION

This work received funding from the Internal Grant Agency (IGA) of the Czech University of Life Sciences Prague under grant agreement No 20184218 (M. Jungová). The authors are grateful for the financial support provided by the Czech Ministry of Agriculture RO0423.

## Supporting information


Appendix S1:

## Data Availability

All data are in supporting information, and they will be available at time of publication.
