# Supplementary material for: Origin and genetic variability of populations of the invasive plant Rumex alpinus L. in the Giant (Krkonoše) Mountains
Source: Ecol Evol. 2023 Jun 4;13(6):e10145. doi: 10.1002/ece3.10145 (PMC10239895; doi:10.1002/ece3.10145)
Supplement: Supplementary file 1 — Appendix S1: [file ECE3-13-e10145-s001.docx]

**Origin and genetic variability of populations of the invasive plant *Rumex alpinus* L. in the Giant (Krkonoše) Mountains**

^1,2^*Michaela Jungová,^1^Vladimíra Müllerová Jurasová, ^2^Petra Hlásná Čepková, ^2^Leona Leišová Svobodová, ^2^Pavel Svoboda, ^2^Michal Hejcman

^1^*Department of Ecology, Faculty of Environmental Sciences, Czech University of Life Sciences Prague, Kamýcká 129, CZ 165 21 Prague 6 – Suchdol, Czech Republic*

*^2^Crop Research Institute, Drnovská 507/73, 161 06 Prague 6 – Ruzyně, Czech Republic*

*^3^Faculty of Environment, Jan Evangelista Purkyně University in Ústí nad Labem, Pasteurova 3544/1, 400 96 Ústí nad Labem, Czech Republic*

*Corresponding author: michaela.jungova @vurv.cz

**
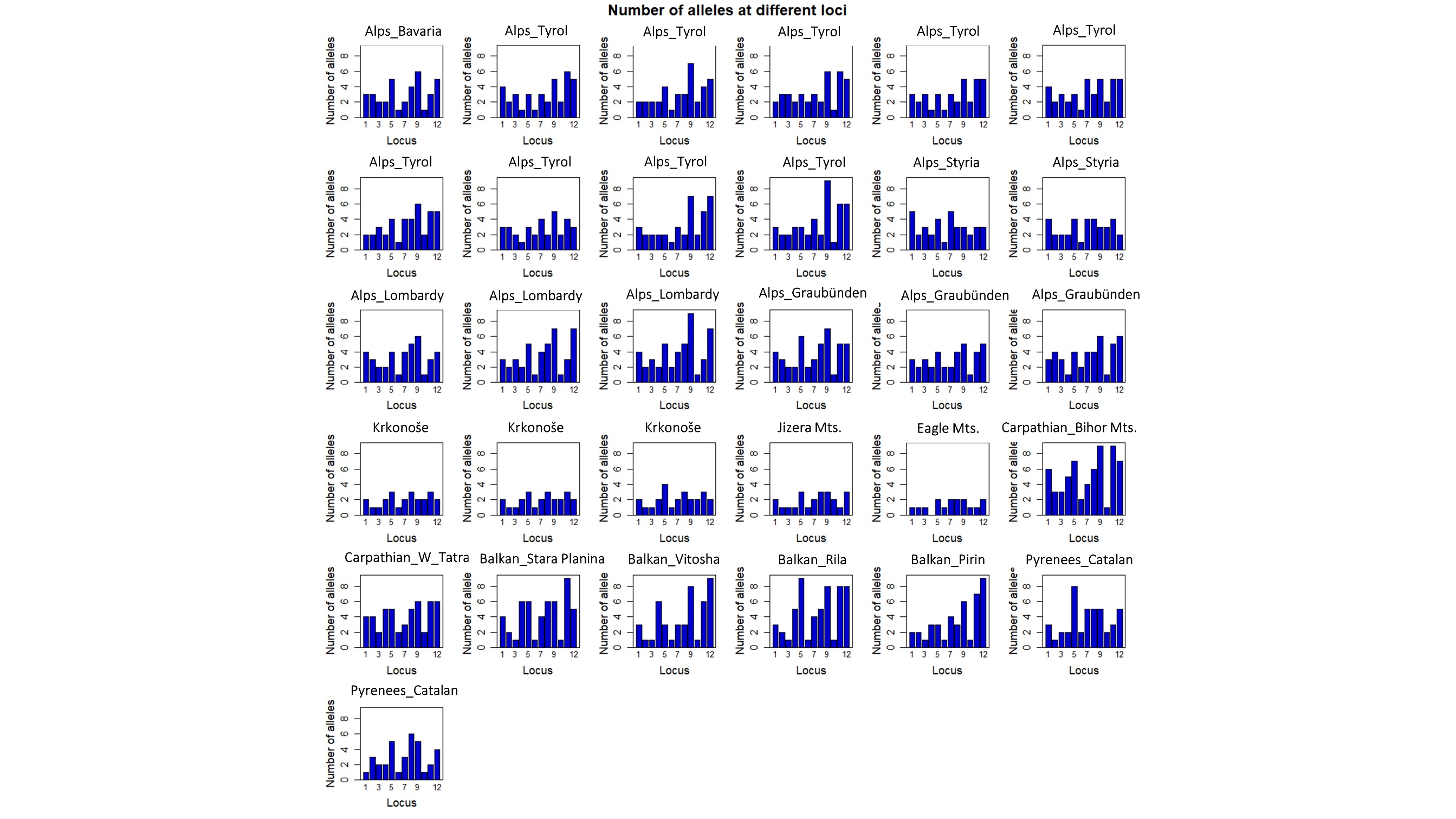
**

**Figure S1** Number of alleles at different loci for all 31 populations of *Rumex alpinus*. Individual populations are named according to mountain regions and correspond with the order listed in Table 1.

**Table S1** Characteristics of 12 microsatelite markers designed for *Rumex alpinus* according to Šurinová et al. (2018). In each of the three multiplexes, the markers were always labeled in the following color order: 1) PET 2) NED 3) FAM 4) VIC.

| **No.** | **Oligo name** | **Sequence (5´-3´)** | **Repeat motif** | **PCR product size range (bp)** |
| --- | --- | --- | --- | --- |
| 1 | Ram 32_M13F | AGTAATATTCTTAGACCATCACCACA | (ACCAC)6 | 111-156 |
|  | Ram 32_R | TGTCGACATGAAGCTGTGAA |  |  |
| 2 | Ram 49_M13F | GCAGAAGCGGAGGAAGATAC | (GAG)7 | 106-112 |
|  | Ram 49_R | CCGCATCGCTACGAGACT |  |  |
| 3 | Ram116_M13F | CAAACAAAAGCCAAACCCAT | (GA)7 | 198-202 |
|  | Ram116_R | TCTTCATTTCTCCCTCGATCTC |  |  |
| 4 | Ram146_M13F | ACCTTAAACCCTGCCCAAAT | (CAA)5 | 302-317 |
|  | Ram146_R | TCACAACGGTCCAAGTCAAA |  |  |
| 5 | Ram 92_M13F | TGTGGAGTAAATTGGGGAATG | (AG)14 | 117-127 |
|  | Ram 92_R | GCCCAGCAGTAGCAGTGTC |  |  |
| 6 | Ram165_M13F | TGCTACACGAGCTGATGCTT | (TTC)6 | 149-152 |
|  | Ram165_R | TTTCTCTGCTCGGATTGGAT |  |  |
| 7 | Ram199_M13F | CCCACGAGTTACCCAATCAAC | (CA)17 | 165-177 |
|  | Ram199_R | TTCTTGACCCTGAATTGTTCG |  |  |
| 8 | Ram200_M13F | AAACTGGCCACATTGAGGT | (AG)14 | 238-248 |
|  | Ram200_R | GTGTCACACTGAAGAACCTG |  |  |
| 9 | Ram 94_M13F | GGGAGAGGGTTTGGAGATTC | (GA)17 | 117-137 |
|  | Ram 94_R | CACAGGCTCACACGTTGTCT |  |  |
| 10 | Ram168_M13F | TCGGATTCTTCGAGAGGAGA | (AG)6 | 107-109 |
|  | Ram168_R | ATTTCCGATTCCCAATTTCC |  |  |
| 11 | Ram193_M13F | GAGTTCCCACCACAAGAAGC | (TC)10 | 173-189 |
|  | Ram193_R | CTAGTCCTGGCCTCTACTGC |  |  |
| 12 | Ram198_M13F | AATGGGATGGATTGGAAGGC | (GA)17 | 147-169 |
|  | Ram198_R | ACTCTCACAGTCACAGGCTC |  |  |
|  |  |  |  |  |

**Table S2**  The table of results of Hardy-Weinberg equilibrium shows the observed (*H_O_*) and expected (*H_E_*) heterozygosity values for 12 different loci separately for the 31 populations. The expected heterozygosity values were calculated using the Hardy-Weinberg equilibrium equation, which predicts the frequency of genotypes based on the frequency of alleles in the population.

| **Populations of *Rumex alpinus*** | | | | | |
| --- | --- | --- | --- | --- | --- |
| **Garmisch Partenkirchen** | **Locus** | **n** | **Ho** | **He** | ***P*-value** |
|  | 1 | 17 | 0.647 | 0.494 | 0.471 |
|  | 2 | 17 | 0.824 | 0.572 | 0.070 |
|  | 3 | 17 | 0.353 | 0.299 | 1.000 |
|  | 4 | 17 | 0.000 | 0.114 | 0.030 |
|  | 5 | 17 | 1.000 | 0.765 | 0.001 |
|  | 6 | This locus is monomorphic | | | |
|  | 7 | 17 | 1.000 | 0.515 | 0.000 |
|  | 8 | 17 | 1.000 | 0.668 | 0.001 |
|  | 9 | 17 | 0.235 | 0.642 | 0.000 |
|  | 10 | This locus is monomorphic | | | |
|  | 11 | 17 | 0.118 | 0.169 | 0.091 |
|  | 12 | 17 | 0.471 | 0.517 | 0.109 |
| **Mean** |  |  | **0.565** | **0.476** |  |
| **Filtzsteiner** | **Locus** | **n** | **Ho** | **He** | ***P*-value** |
|  | 1 | 12 | 0.333 | 0.627 | 0.015 |
|  | 2 | 12 | 0.333 | 0.290 | 1.000 |
|  | 3 | 12 | 0.333 | 0.562 | 0.138 |
|  | 4 | This locus is monomorphic | | | |
|  | 5 | 12 | 0.333 | 0.304 | 1.000 |
|  | 6 | This locus is monomorphic | | | |
|  | 7 | 11 | 0.727 | 0.589 | 0.182 |
|  | 8 | 12 | 0.417 | 0.344 | 1.000 |
|  | 9 | 12 | 0.833 | 0.786 | 0.051 |
|  | 10 | 12 | 0.500 | 0.391 | 0.529 |
|  | 11 | 12 | 0.833 | 0.786 | 0.030 |
|  | 12 | 12 | 0.833 | 0.757 | 0.118 |
| **Mean** |  |  | **0.548** | **0.544** |  |
| **Gerlos** | **Locus** | **n** | **Ho** | **He** | ***P*-value** |
|  | 1 | 9 | 0.222 | 0.523 | 0.171 |
|  | 2 | 9 | 0.222 | 0.471 | 0.167 |
|  | 3 | 9 | 0.556 | 0.425 | 1.000 |
|  | 4 | 9 | 0.222 | 0.209 | 1.000 |
|  | 5 | 9 | 0.556 | 0.542 | 0.497 |
|  | 6 | This locus is monomorphic | | | |
|  | 7 | 8 | 1.000 | 0.658 | 0.063 |
|  | 8 | 9 | 0.778 | 0.569 | 0.337 |
|  | 9 | 8 | 0.750 | 0.850 | 0.104 |
|  | 10 | 9 | 0.111 | 0.111 | 1.000 |
|  | 11 | 9 | 0.556 | 0.634 | 0.335 |
|  | 12 | 8 | 0.500 | 0.708 | 0.057 |
| **Mean** |  |  | **0.497** | **0.518** |  |
| **Almdorf Königsleiten** | **Locus** | **n** | **Ho** | **He** | ***P*-value** |
|  | 1 | 9 | 0.333 | 0.503 | 0.493 |
|  | 2 | 9 | 0.333 | 0.307 | 1.000 |
|  | 3 | 9 | 0.778 | 0.627 | 1.000 |
|  | 4 | 9 | 0.000 | 0.209 | 0.059 |
|  | 5 | 8 | 0.750 | 0.542 | 0.628 |
|  | 6 | 9 | 0.111 | 0.111 | 1.000 |
|  | 7 | 9 | 0.889 | 0.569 | 0.084 |
|  | 8 | 8 | 0.625 | 0.458 | 0.487 |
|  | 9 | 9 | 1.000 | 0.765 | 0.484 |
|  | 10 | This locus is monomorphic | | | |
|  | 11 | 9 | 0.667 | 0.784 | 0.347 |
|  | 12 | 9 | 0.889 | 0.712 | 0.665 |
| **Mean** |  |  | **0.580** | **0.508** |  |
| **Seebachbrücke** | **Locus** | **n** | **Ho** | **He** | ***P*-value** |
|  | 1 | 9 | 0.556 | 0.686 | 1.000 |
|  | 2 | 9 | 0.556 | 0.425 | 1.000 |
|  | 3 | 9 | 0.556 | 0.464 | 1.000 |
|  | 4 | This locus is monomorphic | | | |
|  | 5 | 6 | 0.833 | 0.621 | 0.636 |
|  | 6 | This locus is monomorphic | | | |
|  | 7 | 9 | 0.889 | 0.627 | 0.182 |
|  | 8 | 9 | 0.333 | 0.294 | 1.000 |
|  | 9 | 8 | 1.000 | 0.758 | 0.158 |
|  | 10 | 9 | 0.111 | 0.111 | 1.000 |
|  | 11 | 9 | 0.667 | 0.771 | 0.001 |
|  | 12 | 8 | 0.875 | 0.725 | 0.479 |
| **Mean** |  |  | **0.638** | **0.548** |  |
| **Obervelach** | **Locus** | **n** | **Ho** | **He** | ***P*-value** |
|  | 1 | 11 | 0.636 | 0.671 | 0.268 |
|  | 2 | 11 | 0.091 | 0.091 | 1.000 |
|  | 3 | 11 | 0.636 | 0.589 | 1.000 |
|  | 4 | 11 | 0.091 | 0.091 | 1.000 |
|  | 5 | 8 | 0.750 | 0.542 | 0.628 |
|  | 6 | This locus is monomorphic | | | |
|  | 7 | 8 | 0.875 | 0.758 | 0.075 |
|  | 8 | 11 | 0.636 | 0.558 | 0.726 |
|  | 9 | 10 | 1.000 | 0.695 | 0.132 |
|  | 10 | 11 | 0.818 | 0.524 | 0.091 |
|  | 11 | 10 | 0.700 | 0.747 | 0.018 |
|  | 12 | 9 | 1.000 | 0.706 | 0.234 |
| **Mean** |  |  | **0.658** | **0.543** |  |
| **Mallnitz** | **Locus** | **n** | **Ho** | **He** | ***P*-value** |
|  | 1 | 12 | 0.333 | 0.464 | 0.518 |
|  | 2 | 13 | 0.231 | 0.212 | 1.000 |
|  | 3 | 12 | 0.333 | 0.627 | 0.075 |
|  | 4 | 13 | 0.077 | 0.077 | 1.000 |
|  | 5 | 10 | 0.700 | 0.700 | 0.058 |
|  | 6 | This locus is monomorphic | | | |
|  | 7 | 12 | 0.917 | 0.721 | 0.540 |
|  | 8 | 13 | 0.462 | 0.606 | 0.098 |
|  | 9 | 12 | 0.917 | 0.801 | 0.054 |
|  | 10 | 13 | 0.077 | 0.077 | 1.000 |
|  | 11 | 13 | 0.462 | 0.717 | 0.011 |
|  | 12 | 13 | 1.000 | 0.766 | 0.016 |
| **Mean** |  |  | **0.501** | **0.524** |  |
| **Schildalm** | **Locus** | **n** | **Ho** | **He** | ***P*-value** |
|  | 1 | 13 | 0.462 | 0.566 | 0.004 |
|  | 2 | 13 | 0.231 | 0.335 | 0.375 |
|  | 3 | 13 | 0.385 | 0.508 | 0.574 |
|  | 4 | This locus is monomorphic | | | |
|  | 5 | 12 | 0.500 | 0.649 | 0.021 |
|  | 6 | 13 | 0.231 | 0.323 | 0.374 |
|  | 7 | 11 | 1.000 | 0.688 | 0.058 |
|  | 8 | 13 | 0.308 | 0.271 | 1.000 |
|  | 9 | 13 | 0.846 | 0.683 | 0.011 |
|  | 10 | 13 | 0.077 | 0.077 | 1.000 |
|  | 11 | 13 | 0.846 | 0.729 | 0.534 |
|  | 12 | 13 | 0.692 | 0.649 | 0.329 |
| Mean |  |  | **0.507** | **0.498** |  |
| **Ahrntal_Anholz** | **Locus** | **n** | **Ho** | **He** | ***P*-value** |
|  | 1 | 16 | 0.250 | 0.373 | 0.129 |
|  | 2 | 16 | 0.125 | 0.121 | 1.000 |
|  | 3 | 16 | 0.250 | 0.226 | 1.000 |
|  | 4 | 16 | 0.063 | 0.063 | 1.000 |
|  | 5 | 16 | 0.063 | 0.353 | 0.004 |
|  | 6 | This locus is monomorphic | | | |
|  | 7 | 16 | 1.000 | 0.643 | 0.000 |
|  | 8 | 16 | 0.063 | 0.063 | 1.000 |
|  | 9 | 15 | 1.000 | 0.823 | 0.005 |
|  | 10 | 16 | 0.125 | 0.121 | 1.000 |
|  | 11 | 16 | 0.625 | 0.756 | 0.000 |
|  | 12 | 16 | 1.000 | 0.808 | 0.017 |
| **Mean** |  |  | **0.415** | **0.395** |  |
| **Umbaltal_Prägraten** | **Locus** | **n** | **Ho** | **He** | ***P*-value** |
|  | 1 | 12 | 0.250 | 0.540 | 0.025 |
|  | 2 | 12 | 0.250 | 0.344 | 0.403 |
|  | 3 | 12 | 0.167 | 0.159 | 1.000 |
|  | 4 | 12 | 0.083 | 0.163 | 0.043 |
|  | 5 | 12 | 0.583 | 0.453 | 0.631 |
|  | 6 | 12 | 0.083 | 0.083 | 1.000 |
|  | 7 | 12 | 0.833 | 0.678 | 0.008 |
|  | 8 | 12 | 0.417 | 0.431 | 1.000 |
|  | 9 | 12 | 1.000 | 0.830 | 0.242 |
|  | 10 | This locus is monomorphic | | | |
|  | 11 | 12 | 0.500 | 0.793 | 0.003 |
|  | 12 | 12 | 0.917 | 0.790 | 0.082 |
| **Mean** |  |  | **0.462** | **0.479** |  |
| **Dachstein** | **Locus** | **n** | **Ho** | **He** | ***P*-value** |
|  | 1 | 11 | 0.364 | 0.528 | 0.059 |
|  | 2 | 12 | 0.083 | 0.083 | 1.000 |
|  | 3 | 12 | 0.333 | 0.681 | 0.022 |
|  | 4 | 12 | 0.083 | 0.083 | 1.000 |
|  | 5 | 8 | 1.000 | 0.650 | 0.056 |
|  | 6 | This locus is monomorphic | | | |
|  | 7 | 10 | 1.000 | 0.768 | 0.336 |
|  | 8 | 12 | 1.000 | 0.562 | 0.002 |
|  | 9 | 12 | 1.000 | 0.562 | 0.002 |
|  | 10 | 11 | 0.364 | 0.312 | 1.000 |
|  | 11 | 12 | 1.000 | 0.594 | 0.003 |
|  | 12 | 12 | 1.000 | 0.562 | 0.002 |
| **Mean** |  |  | **0.657** | **0.490** |  |
| **Schladming** | **Locus** | **n** | **Ho** | **He** | ***P*-value** |
|  | 1 | 13 | 0.462 | 0.397 | 1.000 |
|  | 2 | 13 | 0.385 | 0.323 | 1.000 |
|  | 3 | 13 | 0.308 | 0.369 | 0.524 |
|  | 4 | 13 | 0.231 | 0.212 | 1.000 |
|  | 5 | 13 | 0.923 | 0.643 | 0.129 |
|  | 6 | This locus is monomorphic | | | |
|  | 7 | 13 | 0.923 | 0.698 | 0.002 |
|  | 8 | 13 | 1.000 | 0.652 | 0.016 |
|  | 9 | 13 | 1.000 | 0.557 | 0.001 |
|  | 10 | 13 | 0.308 | 0.492 | 0.011 |
|  | 11 | 12 | 0.917 | 0.663 | 0.001 |
|  | 12 | 13 | 1.000 | 0.520 | 0.001 |
| **Mean** |  |  | **0.678** | **0.503** |  |
| **Madesimo** | **Locus** | **n** | **Ho** | **He** | ***P*-value** |
|  | 1 | 12 | 0.833 | 0.667 | 0.006 |
|  | 2 | 13 | 0.308 | 0.465 | 0.417 |
|  | 3 | 13 | 0.308 | 0.271 | 1.000 |
|  | 4 | 13 | 0.154 | 0.148 | 1.000 |
|  | 5 | 13 | 1.000 | 0.665 | 0.007 |
|  | 6 | This locus is monomorphic | | | |
|  | 7 | 13 | 1.000 | 0.662 | 0.013 |
|  | 8 | 12 | 1.000 | 0.710 | 0.018 |
|  | 9 | 13 | 1.000 | 0.785 | 0.288 |
|  | 10 | This locus is monomorphic | | | |
|  | 11 | 12 | 0.833 | 0.649 | 0.668 |
|  | 12 | 12 | 0.583 | 0.707 | 0.000 |
| **Mean** |  |  | **0.702** | **0.573** |  |
| **Valle Spluga** | **Locus** | **n** | **Ho** | **He** | ***P*-value** |
|  | 1 | 12 | 0.500 | 0.605 | 0.793 |
|  | 2 | 12 | 0.417 | 0.518 | 0.594 |
|  | 3 | 12 | 0.417 | 0.554 | 0.548 |
|  | 4 | 12 | 0.083 | 0.228 | 0.131 |
|  | 5 | 11 | 1.000 | 0.736 | 0.198 |
|  | 6 | This locus is monomorphic | | | |
|  | 7 | 12 | 0.917 | 0.630 | 0.137 |
|  | 8 | 10 | 1.000 | 0.768 | 0.843 |
|  | 9 | 11 | 1.000 | 0.827 | 0.406 |
|  | 10 | This locus is monomorphic | | | |
|  | 11 | 12 | 0.333 | 0.420 | 0.311 |
|  | 12 | 8 | 1.000 | 0.867 | 0.240 |
| **Mean** |  |  | **0.667** | **0.615** |  |
| **Splügen** | **Locus** | **n** | **Ho** | **He** | ***P*-value** |
|  | 1 | 15 | 0.400 | 0.609 | 0.041 |
|  | 2 | 15 | 0.267 | 0.515 | 0.114 |
|  | 3 | 15 | 0.333 | 0.522 | 0.141 |
|  | 4 | 15 | 0.267 | 0.405 | 0.226 |
|  | 5 | 15 | 0.933 | 0.740 | 0.118 |
|  | 6 | 15 | 0.133 | 0.129 | 1.000 |
|  | 7 | 15 | 0.800 | 0.697 | 0.152 |
|  | 8 | 15 | 0.867 | 0.722 | 0.277 |
|  | 9 | 15 | 1.000 | 0.839 | 0.768 |
|  | 10 | This locus is monomorphic | | | |
|  | 11 | 15 | 0.267 | 0.439 | 0.079 |
|  | 12 | 14 | 0.857 | 0.825 | 0.101 |
| **Mean** |  |  | **0.557** | **0.586** |  |
| **St. Moritz** | **Locus** | **n** | **Ho** | **He** | ***P*-value** |
|  | 1 | 11 | 0.818 | 0.680 | 0.012 |
|  | 2 | 11 | 0.545 | 0.606 | 0.091 |
|  | 3 | 8 | 0.625 | 0.458 | 0.487 |
|  | 4 | 11 | 0.000 | 0.173 | 0.047 |
|  | 5 | 11 | 0.818 | 0.745 | 0.210 |
|  | 6 | 11 | 0.182 | 0.312 | 0.280 |
|  | 7 | 11 | 1.000 | 0.658 | 0.044 |
|  | 8 | 11 | 0.909 | 0.714 | 0.004 |
|  | 9 | 10 | 1.000 | 0.842 | 0.472 |
|  | 10 | This locus is monomorphic | | | |
|  | 11 | 11 | 0.727 | 0.628 | 0.465 |
|  | 12 | 11 | 0.909 | 0.788 | 0.262 |
| **Mean** |  |  | **0.685** | **0.600** |  |
| **Schuders** | **Locus** | **n** | **Ho** | **He** | ***P*-value** |
|  | 1 | 13 | 0.615 | 0.594 | 0.222 |
|  | 2 | 13 | 0.077 | 0.077 | 1.000 |
|  | 3 | 10 | 0.300 | 0.426 | 0.090 |
|  | 4 | 13 | 0.000 | 0.271 | 0.005 |
|  | 5 | 13 | 0.846 | 0.717 | 0.117 |
|  | 6 | 13 | 0.077 | 0.077 | 1.000 |
|  | 7 | 10 | 0.800 | 0.526 | 0.199 |
|  | 8 | 13 | 0.769 | 0.726 | 0.502 |
|  | 9 | 13 | 1.000 | 0.785 | 0.012 |
|  | 10 | This locus is monomorphic | | | |
|  | 11 | 13 | 0.231 | 0.532 | 0.004 |
|  | 12 | 13 | 1.000 | 0.785 | 0.011 |
| **Mean** |  |  | **0.520** | **0.501** |  |
| **Davos** | **Locus** | **n** | **Ho** | **He** | ***P*-value** |
|  | 1 | 10 | 0.600 | 0.695 | 1.000 |
|  | 2 | 9 | 0.444 | 0.399 | 1.000 |
|  | 3 | 10 | 0.300 | 0.279 | 1.000 |
|  | 4 | This locus is monomorphic | | | |
|  | 5 | 9 | 1.000 | 0.725 | 0.274 |
|  | 6 | 10 | 0.100 | 0.100 | 1.000 |
|  | 7 | 10 | 1.000 | 0.621 | 0.014 |
|  | 8 | 10 | 1.000 | 0.647 | 0.032 |
|  | 9 | 8 | 0.875 | 0.825 | 0.484 |
|  | 10 | This locus is monomorphic | | | |
|  | 11 | 10 | 0.400 | 0.653 | 0.029 |
|  | 12 | 10 | 0.900 | 0.821 | 0.364 |
| **Mean** |  |  | **0.662** | **0.576** |  |
| **Horní Mísečky ●** | **Locus** | **n** | **Ho** | **He** | ***P*-value** |
|  | 1 | 20 | 0.050 | 0.450 | 0.000 |
|  | 2 | This locus is monomorphic | | | |
|  | 3 | This locus is monomorphic | | | |
|  | 4 | 20 | 0.150 | 0.409 | 0.010 |
|  | 5 | 20 | 0.650 | 0.673 | 0.000 |
|  | 6 | This locus is monomorphic | | | |
|  | 7 | 20 | 0.800 | 0.492 | 0.006 |
|  | 8 | 20 | 0.700 | 0.678 | 0.000 |
|  | 9 | 20 | 1.000 | 0.513 | 0.000 |
|  | 10 | 20 | 0.750 | 0.501 | 0.060 |
|  | 11 | 20 | 0.350 | 0.476 | 0.000 |
|  | 12 | 20 | 0.950 | 0.512 | 0.000 |
| **Mean** |  |  | **0.600** | **0.523** |  |
| **Špindl ●** | **Locus** | **n** | **Ho** | **He** | ***P*-value** |
|  | 1 | 17 | 0.118 | 0.214 | 0.178 |
|  | 2 | This locus is monomorphic | | | |
|  | 3 | This locus is monomorphic | | | |
|  | 4 | 17 | 0.059 | 0.059 | 1.000 |
|  | 5 | 17 | 1.000 | 0.590 | 0.000 |
|  | 6 | This locus is monomorphic | | | |
|  | 7 | 17 | 0.941 | 0.513 | 0.000 |
|  | 8 | 15 | 1.000 | 0.632 | 0.001 |
|  | 9 | 17 | 1.000 | 0.515 | 0.000 |
|  | 10 | 17 | 1.000 | 0.515 | 0.000 |
|  | 11 | 17 | 0.176 | 0.219 | 0.180 |
|  | 12 | 17 | 0.824 | 0.499 | 0.010 |
| **Mean** |  |  | **0.680** | **0.417** |  |
| **Velká Úpa ●** | **Locus** | **n** | **Ho** | **He** | ***P*-value** |
|  | 1 | 17 | 0.118 | 0.428 | 0.006 |
|  | 2 | This locus is monomorphic | | | |
|  | 3 | This locus is monomorphic | | | |
|  | 4 | 17 | 0.000 | 0.371 | 0.000 |
|  | 5 | 19 | 0.789 | 0.701 | 0.000 |
|  | 6 | This locus is monomorphic | | | |
|  | 7 | 19 | 0.842 | 0.501 | 0.004 |
|  | 8 | 18 | 0.722 | 0.681 | 0.000 |
|  | 9 | 19 | 1.000 | 0.514 | 0.000 |
|  | 10 | 19 | 1.000 | 0.514 | 0.000 |
|  | 11 | 19 | 0.421 | 0.546 | 0.000 |
|  | 12 | 19 | 1.000 | 0.514 | 0.000 |
| **Mean** |  |  | **0.655** | **0.530** |  |
| **Jizera Mountains ●** | **Locus** | **n** | **Ho** | **He** | ***P*-value** |
|  | 1 | 14 | 0.071 | 0.071 | 1.000 |
|  | 2 | This locus is monomorphic | | | |
|  | 3 | This locus is monomorphic | | | |
|  | 4 | This locus is monomorphic | | | |
|  | 5 | 14 | 0.929 | 0.585 | 0.000 |
|  | 6 | This locus is monomorphic | | | |
|  | 7 | 14 | 1.000 | 0.519 | 0.001 |
|  | 8 | 14 | 0.929 | 0.585 | 0.000 |
|  | 9 | 14 | 1.000 | 0.606 | 0.001 |
|  | 10 | 14 | 1.000 | 0.519 | 0.001 |
|  | 11 | This locus is monomorphic | | | |
|  | 12 | 14 | 0.929 | 0.611 | 0.006 |
| **Mean** |  |  | **0.837** | **0.499** |  |
| **Eagle Mountains ●** | **Locus** | **n** | **Ho** | **He** | ***P*-value** |
|  | 1 | This locus is monomorphic | | | |
|  | 2 | This locus is monomorphic | | | |
|  | 3 | This locus is monomorphic | | | |
|  | 4 | This locus is monomorphic | | | |
|  | 5 | 18 | 1.000 | 0.514 | 0.000 |
|  | 6 |  |  |  |  |
|  | 7 | 18 | 1.000 | 0.514 | 0.000 |
|  | 8 | 18 | 1.000 | 0.514 | 0.000 |
|  | 9 | 18 | 1.000 | 0.514 | 0.000 |
|  | 10 | This locus is monomorphic | | | |
|  | 11 | This locus is monomorphic | | | |
|  | 12 | 18 | 1.000 | 0.514 | 0.000 |
| **Mean** |  |  | **1.000** | **0.514** |  |
| **Romania_Muntele Mic** | **Locus** | **n** | **Ho** | **He** | ***P*-value** |
|  | 1 | 16 | 0.438 | 0.744 | 0.016 |
|  | 2 | 16 | 0.250 | 0.234 | 1.000 |
|  | 3 | 16 | 0.063 | 0.123 | 0.032 |
|  | 4 | 16 | 0.500 | 0.581 | 0.087 |
|  | 5 | 15 | 1.000 | 0.841 | 0.542 |
|  | 6 | 16 | 0.000 | 0.226 | 0.003 |
|  | 7 | 16 | 0.250 | 0.286 | 0.045 |
|  | 8 | 16 | 0.938 | 0.823 | 0.353 |
|  | 9 | 15 | 1.000 | 0.871 | 0.831 |
|  | 10 | This locus is monomorphic | | | |
|  | 11 | 16 | 0.813 | 0.837 | 0.131 |
|  | 12 | 14 | 0.857 | 0.839 | 0.629 |
| **Mean** |  |  | **0.555** | **0.582** |  |
| **Zakopane_Lejowa glade** | **Locus** | **n** | **Ho** | **He** | ***P*-value** |
|  | 1 | 15 | 0.267 | 0.356 | 0.296 |
|  | 2 | 15 | 0.200 | 0.697 | 0.000 |
|  | 3 | 15 | 0.067 | 0.067 | 1.000 |
|  | 4 | 15 | 0.267 | 0.680 | 0.001 |
|  | 5 | 15 | 0.800 | 0.763 | 0.014 |
|  | 6 | 15 | 0.067 | 0.067 | 1.000 |
|  | 7 | 15 | 0.200 | 0.453 | 0.021 |
|  | 8 | 15 | 0.867 | 0.770 | 0.002 |
|  | 9 | 15 | 0.933 | 0.786 | 0.018 |
|  | 10 | 15 | 0.067 | 0.067 | 1.000 |
|  | 11 | 14 | 0.857 | 0.767 | 0.000 |
|  | 12 | 15 | 0.933 | 0.786 | 0.017 |
| **Mean** |  |  | **0.460** | **0.522** |  |
| **Kom_Stara Planina** | **Locus** | **n** | **Ho** | **He** | ***P*-value** |
|  | 1 | 13 | 0.385 | 0.603 | 0.198 |
|  | 2 | 14 | 0.429 | 0.423 | 1.000 |
|  | 3 | This locus is monomorphic | | | |
|  | 4 | 14 | 0.786 | 0.802 | 0.123 |
|  | 5 | 13 | 0.462 | 0.566 | 0.131 |
|  | 6 | This locus is monomorphic | | | |
|  | 7 | 14 | 0.571 | 0.653 | 0.399 |
|  | 8 | 14 | 0.500 | 0.579 | 0.284 |
|  | 9 | 14 | 1.000 | 0.820 | 0.180 |
|  | 10 | This locus is monomorphic | | | |
|  | 11 | 13 | 1.000 | 0.895 | 0.265 |
|  | 12 | 13 | 1.000 | 0.760 | 0.361 |
| **Mean** |  |  | **0.681** | **0,678** |  |
| **Vitosha_Sofia** | **Locus** | **n** | **Ho** | **He** | ***P*-value** |
|  | 1 | 10 | 0.700 | 0.653 | 0.371 |
|  | 2 | This locus is monomorphic | | | |
|  | 3 | This locus is monomorphic | | | |
|  | 4 | 11 | 0.545 | 0.788 | 0.099 |
|  | 5 | 10 | 0.600 | 0.584 | 0.447 |
|  | 6 | This locus is monomorphic | | | |
|  | 7 | 10 | 0.700 | 0.626 | 1.000 |
|  | 8 | 11 | 0.727 | 0.623 | 0.825 |
|  | 9 | 10 | 0.800 | 0.868 | 0.433 |
|  | 10 | This locus is monomorphic | | | |
|  | 11 | 11 | 1.000 | 0.797 | 0.508 |
|  | 12 | 11 | 0.818 | 0.879 | 0.202 |
| **Mean** |  |  | **0.736** | **0.727** |  |
| **Rila_Borovets_Yastrebets** | **Locus** | **n** | **Ho** | **He** | ***P*-value** |
|  | 1 | 13 | 0.231 | 0.594 | 0.004 |
|  | 2 | 14 | 0.000 | 0.138 | 0.037 |
|  | 3 | This locus is monomorphic | | | |
|  | 4 | 13 | 0.538 | 0.640 | 0.109 |
|  | 5 | 14 | 0.714 | 0.865 | 0.017 |
|  | 6 | This locus is monomorphic | | | |
|  | 7 | 13 | 0.077 | 0.742 | 0.000 |
|  | 8 | 13 | 0.308 | 0.631 | 0.001 |
|  | 9 | 12 | 0.833 | 0.877 | 0.044 |
|  | 10 | This locus is monomorphic | | | |
|  | 11 | 14 | 1.000 | 0.868 | 0.000 |
|  | 12 | 14 | 0.857 | 0.862 | 0.025 |
| **Mean** |  |  | **0.507** | **0.691** |  |
| **Pirin_Bansko SKI** | **Locus** | **n** | **Ho** | **He** | ***P*-value** |
|  | 1 | 13 | 0.538 | 0.508 | 1.000 |
|  | 2 | 13 | 0.077 | 0.077 | 1.000 |
|  | 3 | This locus is monomorphic | | | |
|  | 4 | 13 | 0.615 | 0.551 | 1.000 |
|  | 5 | 12 | 0.250 | 0.236 | 1.000 |
|  | 6 | This locus is monomorphic | | | |
|  | 7 | 12 | 0.417 | 0.572 | 0.364 |
|  | 8 | 13 | 0.231 | 0.218 | 1.000 |
|  | 9 | 11 | 0.818 | 0.840 | 0.606 |
|  | 10 | This locus is monomorphic | | | |
|  | 11 | 13 | 0.846 | 0.735 | 0.001 |
|  | 12 | 13 | 0.846 | 0.895 | 0.048 |
| **Mean** |  |  | **0.515** | **0.515** |  |
| **Prats d´Aiguadassi** | **Locus** | **n** | **Ho** | **He** | ***P*-value** |
|  | 1 | 11 | 0.091 | 0.255 | 0.048 |
|  | 2 | This locus is monomorphic | | | |
|  | 3 | 12 | 0.083 | 0.083 | 1.000 |
|  | 4 | 12 | 0.167 | 0.159 | 1.000 |
|  | 5 | 9 | 0.778 | 0.837 | 0.080 |
|  | 6 | 12 | 0.000 | 0.290 | 0.006 |
|  | 7 | 8 | 0.750 | 0.750 | 0.730 |
|  | 8 | 7 | 1.000 | 0.791 | 0.302 |
|  | 9 | 10 | 0.800 | 0.768 | 0.641 |
|  | 10 | 12 | 0.083 | 0.083 | 1.000 |
|  | 11 | 12 | 0.250 | 0.475 | 0.017 |
|  | 12 | 10 | 0.900 | 0.789 | 0.206 |
| **Mean** |  |  | **0.446** | **0.480** |  |
| **Aigüestortes** | **Locus** | **n** | **Ho** | **He** | ***P*-value** |
|  | 1 | This locus is monomorphic | | | |
|  | 2 | 14 | 0.000 | 0.265 | 0.001 |
|  | 3 | 13 | 0.077 | 0.077 | 1.000 |
|  | 4 | 14 | 0.143 | 0.138 | 1.000 |
|  | 5 | 14 | 1.000 | 0.807 | 0.000 |
|  | 6 | This locus is monomorphic | | | |
|  | 7 | 14 | 1.000 | 0.606 | 0.001 |
|  | 8 | 14 | 1.000 | 0.725 | 0.004 |
|  | 9 | 14 | 0.571 | 0.722 | 0.199 |
|  | 10 | This locus is monomorphic | | | |
|  | 11 | 14 | 0.429 | 0.423 | 1.000 |
|  | 12 | 14 | 0.571 | 0.558 | 0.470 |
| **Mean** |  |  | **0.532** | **0.480** |  |

| **Table S3** The raw allelic data**PopKod** | **population** | **Mountains** | **kod** | **Ram32** | **Ram32** | **Ram49** | **Ram49** | **Ram116** | **Ram116** | **Ram146** | **Ram146** | **Ram92** | **Ram92** | **Ram165** | **Ram165** | **Ram199** | **Ram199** | **Ram200** | **Ram200** | **Ram94** | **Ram94** | **Ram168** | **Ram168** | **Ram193** | **Ram193** | **Ram198** | **Ram198** |
| --- | --- | --- | --- | --- | --- | --- | --- | --- | --- | --- | --- | --- | --- | --- | --- | --- | --- | --- | --- | --- | --- | --- | --- | --- | --- | --- | --- |
| Pop1 | Bv_1 | Alps | Bavaria | 125 | 130 | 108 | 111 | 198 | 198 | 304 | 304 | 124 | 128 | 153 | 153 | 163 | 165 | 242 | 246 | 130 | 132 | 109 | 109 | 174 | 174 | 159 | 161 |
| Pop1 | Bv_1 | Alps | Bavaria | 125 | 130 | 108 | 111 | 198 | 198 | 304 | 304 | 122 | 124 | 153 | 153 | 163 | 165 | 240 | 242 | 120 | 120 | 109 | 109 | 174 | 174 | 149 | 149 |
| Pop1 | Bv_1 | Alps | Bavaria | 125 | 130 | 108 | 111 | 198 | 198 | 304 | 304 | 122 | 124 | 153 | 153 | 163 | 165 | 240 | 242 | 120 | 120 | 109 | 109 | 174 | 174 | 149 | 149 |
| Pop1 | Bv_1 | Alps | Bavaria | 125 | 130 | 108 | 111 | 198 | 198 | 304 | 304 | 124 | 126 | 153 | 153 | 163 | 165 | 242 | 244 | 120 | 120 | 109 | 109 | 174 | 174 | 149 | 149 |
| Pop1 | Bv_1 | Alps | Bavaria | 125 | 130 | 108 | 111 | 198 | 198 | 304 | 304 | 124 | 128 | 153 | 153 | 163 | 165 | 242 | 246 | 120 | 120 | 109 | 109 | 174 | 174 | 149 | 171 |
| Pop1 | Bv_1 | Alps | Bavaria | 125 | 130 | 108 | 111 | 198 | 198 | 304 | 304 | 124 | 128 | 153 | 153 | 163 | 165 | 242 | 246 | 120 | 120 | 109 | 109 | 174 | 174 | 149 | 171 |
| Pop1 | Bv_1 | Alps | Bavaria | 125 | 130 | 108 | 111 | 198 | 198 | 304 | 304 | 124 | 128 | 153 | 153 | 163 | 165 | 242 | 246 | 120 | 120 | 109 | 109 | 174 | 174 | 149 | 149 |
| Pop1 | Bv_1 | Alps | Bavaria | 125 | 130 | 105 | 108 | 198 | 198 | 304 | 304 | 122 | 124 | 153 | 153 | 163 | 165 | 240 | 242 | 140 | 142 | 109 | 109 | 174 | 174 | 171 | 171 |
| Pop1 | Bv_1 | Alps | Bavaria | 130 | 130 | 111 | 111 | 198 | 198 | 304 | 304 | 124 | 126 | 153 | 153 | 163 | 165 | 244 | 246 | 120 | 120 | 109 | 109 | 174 | 174 | 149 | 149 |
| Pop1 | Bv_1 | Alps | Bavaria | 130 | 130 | 111 | 111 | 198 | 198 | 304 | 304 | 122 | 124 | 153 | 153 | 163 | 165 | 240 | 242 | 120 | 120 | 109 | 109 | 174 | 174 | 149 | 149 |
| Pop1 | Bv_1 | Alps | Bavaria | 130 | 130 | 111 | 111 | 198 | 198 | 304 | 304 | 122 | 124 | 153 | 153 | 163 | 165 | 240 | 242 | 120 | 120 | 109 | 109 | 174 | 174 | 149 | 149 |
| Pop1 | Bv_1 | Alps | Bavaria | 115 | 130 | 108 | 111 | 196 | 198 | 301 | 301 | 120 | 122 | 153 | 153 | 163 | 165 | 240 | 242 | 118 | 118 | 109 | 109 | 174 | 174 | 149 | 149 |
| Pop1 | Bv_1 | Alps | Bavaria | 115 | 130 | 105 | 111 | 196 | 198 | 304 | 304 | 120 | 122 | 153 | 153 | 163 | 165 | 240 | 242 | 118 | 140 | 109 | 109 | 182 | 184 | 149 | 171 |
| Pop1 | Bv_1 | Alps | Bavaria | 130 | 130 | 105 | 111 | 196 | 198 | 304 | 304 | 120 | 122 | 153 | 153 | 163 | 165 | 240 | 242 | 118 | 140 | 109 | 109 | 174 | 174 | 149 | 171 |
| Pop1 | Bv_1 | Alps | Bavaria | 130 | 130 | 108 | 111 | 196 | 198 | 304 | 304 | 120 | 122 | 153 | 153 | 163 | 165 | 240 | 242 | 118 | 118 | 109 | 109 | 174 | 174 | 147 | 149 |
| Pop1 | Bv_1 | Alps | Bavaria | 130 | 130 | 108 | 111 | 196 | 198 | 304 | 304 | 120 | 122 | 153 | 153 | 163 | 165 | 240 | 242 | 118 | 118 | 109 | 109 | 174 | 174 | 147 | 149 |
| Pop1 | Bv_1 | Alps | Bavaria | 115 | 130 | 108 | 111 | 196 | 198 | 304 | 304 | 120 | 122 | 153 | 153 | 163 | 165 | 240 | 242 | 118 | 118 | 109 | 109 | 174 | 184 | 147 | 149 |
| Pop2 | TI_1 | Alps | Tyrol | 110 | 130 | 111 | 111 | 194 | 194 | 304 | 304 | 122 | 122 | 153 | 153 | 163 | 165 | 240 | 240 | 128 | 130 | 109 | 109 | 174 | 188 | 157 | 159 |
| Pop2 | TI_1 | Alps | Tyrol | 130 | 130 | 111 | 111 | 194 | 196 | 304 | 304 | 122 | 124 | 153 | 153 | 161 | 163 | 240 | 242 | 130 | 138 | 109 | 111 | 174 | 188 | 159 | 167 |
| Pop2 | TI_1 | Alps | Tyrol | 115 | 115 | 108 | 111 | 196 | 196 | 304 | 304 | 122 | 122 | 153 | 153 | 165 | 165 | 240 | 240 | 136 | 138 | 109 | 111 | 180 | 180 | 165 | 167 |
| Pop2 | TI_1 | Alps | Tyrol | 130 | 130 | 111 | 111 | 198 | 198 | 304 | 304 | 122 | 122 | 153 | 153 | 165 | 165 | 240 | 240 | 128 | 128 | 109 | 109 | 186 | 188 | 159 | 159 |
| Pop2 | TI_1 | Alps | Tyrol | 115 | 120 | 108 | 111 | 198 | 198 | 304 | 304 | 120 | 122 | 153 | 153 | 163 | 165 | 240 | 242 | 126 | 138 | 109 | 111 | 188 | 190 | 157 | 169 |
| Pop2 | TI_1 | Alps | Tyrol | 115 | 115 | 111 | 111 | 196 | 198 | 304 | 304 | 122 | 122 | 153 | 153 | 163 | 165 | 240 | 242 | 128 | 130 | 109 | 111 | 188 | 190 | 157 | 159 |
| Pop2 | TI_1 | Alps | Tyrol | 130 | 130 | 111 | 111 | 198 | 198 | 304 | 304 | 122 | 122 | 153 | 153 | 161 | 163 | 240 | 240 | 128 | 128 | 109 | 109 | 186 | 188 | 159 | 159 |
| Pop2 | TI_1 | Alps | Tyrol | 115 | 120 | 108 | 111 | 198 | 198 | 304 | 304 | 120 | 122 | 153 | 153 | *50* | *50* | 240 | 242 | 126 | 138 | 109 | 111 | 188 | 190 | 157 | 169 |
| Pop2 | TI_1 | Alps | Tyrol | 130 | 130 | 111 | 111 | 194 | 198 | 304 | 304 | 122 | 122 | 153 | 153 | 163 | 165 | 240 | 240 | 130 | 138 | 109 | 109 | 186 | 188 | 159 | 167 |
| Pop2 | TI_1 | Alps | Tyrol | 115 | 115 | 111 | 111 | 194 | 198 | 304 | 304 | 122 | 124 | 153 | 153 | 165 | 165 | 240 | 242 | 130 | 138 | 109 | 109 | 184 | 184 | 159 | 167 |
| Pop2 | TI_1 | Alps | Tyrol | 115 | 130 | 111 | 111 | 198 | 198 | 304 | 304 | 122 | 122 | 153 | 153 | 163 | 165 | 240 | 240 | 136 | 138 | 109 | 111 | 180 | 188 | 165 | 167 |
| Pop2 | TI_1 | Alps | Tyrol | 130 | 130 | 108 | 111 | 198 | 198 | 304 | 304 | 122 | 122 | 153 | 153 | 163 | 165 | 240 | 240 | 128 | 130 | 109 | 109 | 188 | 190 | 157 | 159 |
| Pop3 | TI_2 | Alps | Tyrol | 115 | 115 | 111 | 111 | 194 | 194 | 304 | 304 | 122 | 122 | 153 | 153 | 163 | 165 | 240 | 242 | ***50*** | ***50*** | 109 | 109 | 174 | 184 | 167 | 167 |
| Pop3 | TI_2 | Alps | Tyrol | 115 | 130 | 108 | 108 | 194 | 194 | 304 | 304 | 120 | 122 | 153 | 153 | 163 | 165 | 240 | 242 | 134 | 136 | 109 | 109 | 174 | 184 | 165 | 167 |
| Pop3 | TI_2 | Alps | Tyrol | 130 | 130 | 111 | 111 | 194 | 198 | 304 | 304 | 122 | 124 | 153 | 153 | 163 | 165 | 240 | 242 | 128 | 142 | 109 | 109 | 174 | 174 | 157 | 171 |
| Pop3 | TI_2 | Alps | Tyrol | 115 | 115 | 108 | 111 | 194 | 198 | 304 | 304 | 122 | 122 | 153 | 153 | 163 | 165 | 242 | 242 | 126 | 126 | 109 | 109 | 184 | 184 | 157 | 157 |
| Pop3 | TI_2 | Alps | Tyrol | 130 | 130 | 111 | 111 | 194 | 198 | 304 | 304 | 122 | 122 | 153 | 153 | 161 | 163 | 242 | 244 | 126 | 128 | 109 | 109 | 184 | 188 | 155 | 157 |
| Pop3 | TI_2 | Alps | Tyrol | 130 | 130 | 111 | 111 | 194 | 198 | 304 | 316 | 122 | 124 | 153 | 153 | 161 | 163 | 240 | 242 | 128 | 132 | 109 | 111 | 184 | 184 | 157 | 157 |
| Pop3 | TI_2 | Alps | Tyrol | 115 | 115 | 108 | 111 | 194 | 198 | 304 | 304 | 122 | 122 | 153 | 153 | *50* | *50* | 242 | 242 | 126 | 126 | 109 | 109 | 184 | 184 | 157 | 157 |
| Pop3 | TI_2 | Alps | Tyrol | 115 | 130 | 108 | 108 | 194 | 194 | 304 | 304 | 120 | 122 | 153 | 153 | 163 | 165 | 240 | 242 | 134 | 136 | 109 | 109 | 174 | 184 | 165 | 167 |
| Pop3 | TI_2 | Alps | Tyrol | 130 | 130 | 111 | 111 | 194 | 194 | 304 | 316 | 124 | 126 | 153 | 153 | 161 | 163 | 242 | 244 | 128 | 138 | 109 | 109 | 188 | 190 | ***50*** | ***50*** |
| Pop4 | TI_3 | Alps | Tyrol | 115 | 115 | 108 | 111 | 198 | 198 | 304 | 304 | 122 | 124 | 153 | 153 | 163 | 165 | 240 | 242 | 130 | 138 | 109 | 109 | 188 | 190 | 159 | 167 |
| Pop4 | TI_3 | Alps | Tyrol | 115 | 115 | 105 | 111 | 196 | 198 | 316 | 316 | 122 | 124 | 150 | 153 | 163 | 165 | 240 | 242 | 128 | 138 | 109 | 109 | 188 | 194 | 157 | 167 |
| Pop4 | TI_3 | Alps | Tyrol | 130 | 130 | 108 | 111 | 196 | 198 | 304 | 304 | 120 | 122 | 153 | 153 | 163 | 163 | 240 | 240 | 136 | 138 | 109 | 109 | 174 | 174 | 165 | 167 |
| Pop4 | TI_3 | Alps | Tyrol | 115 | 130 | 111 | 111 | 194 | 198 | 304 | 304 | 122 | 124 | 153 | 153 | 163 | 165 | 240 | 242 | 130 | 138 | 109 | 109 | 174 | 188 | 159 | 167 |
| Pop4 | TI_3 | Alps | Tyrol | 115 | 130 | 111 | 111 | 194 | 198 | 304 | 304 | 122 | 122 | 153 | 153 | 161 | 163 | 240 | 240 | 138 | 142 | 109 | 109 | 174 | 188 | 167 | 167 |
| Pop4 | TI_3 | Alps | Tyrol | 115 | 130 | 111 | 111 | 194 | 198 | 304 | 304 | 122 | 124 | 153 | 153 | 163 | 165 | 240 | 242 | 136 | 138 | 109 | 109 | 174 | 174 | 165 | 167 |
| Pop4 | TI_3 | Alps | Tyrol | 130 | 130 | 111 | 111 | 196 | 198 | 304 | 304 | 122 | 122 | 153 | 153 | 163 | 165 | 240 | 240 | 128 | 138 | 109 | 109 | 184 | 188 | 157 | 167 |
| Pop4 | TI_3 | Alps | Tyrol | 115 | 115 | 111 | 111 | 198 | 198 | 304 | 304 | 122 | 124 | 153 | 153 | 163 | 165 | 240 | 242 | 126 | 128 | 109 | 109 | 184 | 184 | 155 | 157 |
| Pop4 | TI_3 | Alps | Tyrol | 115 | 115 | 111 | 111 | 194 | 196 | 304 | 304 | ***50*** | ***50*** | 153 | 153 | 163 | 165 | ***50*** | ***50*** | 128 | 138 | 109 | 109 | 186 | 188 | 157 | 167 |
| Pop5 | TI_4 | Alps | Tyrol | 120 | 120 | 111 | 111 | 198 | 198 | 304 | 304 | 120 | 122 | 153 | 153 | 163 | 163 | 240 | 240 | 136 | 138 | 109 | 109 | 180 | 180 | 165 | 167 |
| Pop5 | TI_4 | Alps | Tyrol | 115 | 130 | 108 | 111 | 194 | 198 | 304 | 304 | 122 | 124 | 153 | 153 | 163 | 165 | 240 | 242 | 128 | 138 | 109 | 111 | 188 | 190 | 157 | 167 |
| Pop5 | TI_4 | Alps | Tyrol | 130 | 130 | 111 | 111 | 198 | 198 | 304 | 304 | ***50*** | ***50*** | 153 | 153 | 163 | 165 | 240 | 240 | 136 | 138 | 109 | 109 | 188 | 190 | 165 | 167 |
| Pop5 | TI_4 | Alps | Tyrol | 115 | 130 | 111 | 111 | 194 | 198 | 304 | 304 | ***50*** | ***50*** | 153 | 153 | 161 | 163 | 240 | 242 | ***50*** | ***50*** | 109 | 109 | 174 | 184 | ***50*** | ***50*** |
| Pop5 | TI_4 | Alps | Tyrol | ***115*** | ***115*** | 111 | 111 | 198 | 198 | 304 | 304 | 122 | 122 | 153 | 153 | 161 | 163 | 240 | 240 | 136 | 138 | 109 | 109 | 188 | 190 | 165 | 167 |
| Pop5 | TI_4 | Alps | Tyrol | 120 | 130 | 108 | 111 | 196 | 198 | 304 | 304 | 122 | 124 | 153 | 153 | 161 | 163 | 240 | 242 | 136 | 138 | 109 | 109 | 190 | 190 | 167 | 167 |
| Pop5 | TI_4 | Alps | Tyrol | 115 | 115 | 108 | 111 | 196 | 198 | 304 | 304 | 120 | 122 | 153 | 153 | 163 | 165 | 240 | 240 | 126 | 128 | 109 | 109 | 174 | 174 | 155 | 157 |
| Pop5 | TI_4 | Alps | Tyrol | 115 | 120 | 108 | 111 | 198 | 198 | 304 | 304 | ***50*** | ***50*** | 153 | 153 | 163 | 165 | 240 | 240 | 138 | 144 | 109 | 109 | 188 | 190 | 167 | 173 |
| Pop5 | TI_4 | Alps | Tyrol | 115 | 120 | 108 | 111 | 196 | 198 | 304 | 304 | 120 | 122 | 153 | 153 | 161 | 163 | 240 | 240 | 138 | 144 | 109 | 109 | 188 | 190 | 167 | 173 |
| Pop6 | TI_5 | Alps | Tyrol | 115 | 130 | 111 | 111 | 194 | 196 | 304 | 304 | 122 | 122 | 153 | 153 | 165 | 167 | 240 | 242 | 130 | 138 | 109 | 111 | 188 | 190 | 159 | 167 |
| Pop6 | TI_5 | Alps | Tyrol | 115 | 130 | 111 | 111 | 196 | 196 | 304 | 304 | 122 | 124 | 153 | 153 | 159 | 161 | 240 | 242 | 136 | 138 | 109 | 111 | 188 | 190 | 165 | 167 |
| Pop6 | TI_5 | Alps | Tyrol | 130 | 130 | 111 | 111 | 194 | 198 | 304 | 304 | 122 | 124 | 153 | 153 | 165 | 165 | 242 | 242 | 136 | 138 | 109 | 111 | 188 | 190 | 165 | 167 |
| Pop6 | TI_5 | Alps | Tyrol | 130 | 130 | 108 | 111 | 196 | 196 | 304 | 316 | 122 | 122 | 153 | 153 | 165 | 167 | 242 | 242 | 136 | 138 | 109 | 109 | *50* | *50* | 165 | 167 |
| Pop6 | TI_5 | Alps | Tyrol | 115 | 130 | 111 | 111 | 194 | 194 | 304 | 304 | 122 | 124 | 153 | 153 | 165 | 167 | 240 | 242 | 130 | 138 | 109 | 111 | 184 | 190 | 159 | 167 |
| Pop6 | TI_5 | Alps | Tyrol | 135 | 135 | 111 | 111 | 194 | 196 | 304 | 304 | ***50*** | ***50*** | 153 | 153 | 159 | 161 | 240 | 242 | 136 | 138 | 109 | 111 | 180 | 180 | 165 | 167 |
| Pop6 | TI_5 | Alps | Tyrol | 115 | 120 | 111 | 111 | 194 | 196 | 304 | 304 | 122 | 124 | 153 | 153 | ***50*** | ***50*** | 242 | 242 | 130 | 138 | 109 | 111 | 180 | 180 | 159 | 167 |
| Pop6 | TI_5 | Alps | Tyrol | 130 | 135 | 111 | 111 | 194 | 196 | 304 | 304 | ***50*** | ***50*** | 153 | 153 | ***50*** | ***50*** | 240 | 240 | 136 | 138 | 109 | 111 | 180 | 190 | ***50*** | ***50*** |
| Pop6 | TI_5 | Alps | Tyrol | 115 | 130 | 111 | 111 | 194 | 196 | 304 | 304 | 120 | 122 | 153 | 153 | 163 | 165 | 238 | 240 | 128 | 138 | 109 | 111 | 174 | 190 | 157 | 167 |
| Pop6 | TI_5 | Alps | Tyrol | 115 | 115 | 111 | 111 | 196 | 198 | 304 | 304 | ***50*** | ***50*** | 153 | 153 | 165 | 167 | 240 | 242 | ***50*** | ***50*** | 111 | 111 | 180 | 180 | ***50*** | ***50*** |
| Pop6 | TI_5 | Alps | Tyrol | 115 | 130 | 111 | 111 | 196 | 196 | 304 | 304 | 122 | 124 | 153 | 153 | ***50*** | ***50*** | 240 | 242 | 126 | 138 | 109 | 111 | 188 | 190 | 155 | 167 |
| Pop7 | TI_6 | Alps | Tyrol | 115 | 130 | 111 | 111 | 198 | 198 | 304 | 304 | 120 | 122 | 153 | 153 | 163 | 165 | 240 | 240 | 136 | 138 | 109 | 109 | 188 | 190 | 165 | 167 |
| Pop7 | TI_6 | Alps | Tyrol | 115 | 130 | 111 | 111 | 198 | 198 | 304 | 304 | 120 | 122 | 153 | 153 | 163 | 167 | 240 | 240 | 138 | 142 | 109 | 111 | 180 | 180 | 167 | 171 |
| Pop7 | TI_6 | Alps | Tyrol | 115 | 115 | 108 | 111 | 196 | 198 | 304 | 304 | 120 | 122 | 153 | 153 | 163 | 165 | 240 | 240 | 136 | 138 | 109 | 109 | 180 | 180 | 165 | 167 |
| Pop7 | TI_6 | Alps | Tyrol | 115 | 130 | 111 | 111 | 196 | 196 | 304 | 304 | 122 | 124 | 153 | 153 | 163 | 165 | 240 | 242 | ***50*** | ***50*** | 109 | 109 | 180 | 188 | 167 | 171 |
| Pop7 | TI_6 | Alps | Tyrol | 115 | 115 | 108 | 111 | 194 | 198 | 304 | 316 | 120 | 120 | 153 | 153 | 161 | 163 | 240 | 240 | 128 | 128 | 109 | 109 | 184 | 184 | 157 | 159 |
| Pop7 | TI_6 | Alps | Tyrol | 115 | 115 | 108 | 111 | 194 | 194 | 304 | 304 | 120 | 120 | 153 | 153 | 165 | 167 | 242 | 242 | 134 | 136 | 109 | 109 | 180 | 184 | 157 | 159 |
| Pop7 | TI_6 | Alps | Tyrol | 115 | 115 | 111 | 111 | 198 | 198 | 304 | 304 | 120 | 120 | 153 | 153 | 165 | 167 | 242 | 242 | 130 | 138 | 109 | 109 | 184 | 184 | 159 | 167 |
| Pop7 | TI_6 | Alps | Tyrol | 130 | 130 | 111 | 111 | 196 | 196 | 304 | 304 | ***50*** | ***50*** | 153 | 153 | 165 | 167 | 240 | 240 | 130 | 138 | 109 | 109 | 186 | 188 | 159 | 167 |
| Pop7 | TI_6 | Alps | Tyrol | 115 | 130 | 111 | 111 | 196 | 198 | 304 | 304 | ***50*** | ***50*** | 153 | 153 | 163 | 165 | 240 | 242 | 130 | 138 | 109 | 109 | 184 | 188 | 159 | 167 |
| Pop7 | TI_6 | Alps | Tyrol | ***50*** | ***50*** | 111 | 111 | ***50*** | ***50*** | 304 | 304 | 122 | 124 | 153 | 153 | ***50*** | ***50*** | 240 | 242 | 128 | 130 | 109 | 109 | 188 | 190 | 157 | 159 |
| Pop7 | TI_6 | Alps | Tyrol | 130 | 130 | 111 | 111 | 196 | 198 | 304 | 304 | 122 | 124 | 153 | 153 | 163 | 163 | 240 | 242 | 130 | 138 | 109 | 109 | 180 | 180 | 159 | 167 |
| Pop7 | TI_6 | Alps | Tyrol | 115 | 115 | 111 | 111 | 196 | 196 | 304 | 304 | 124 | 128 | 153 | 153 | 163 | 167 | 242 | 246 | 128 | 130 | 109 | 109 | 180 | 180 | 157 | 159 |
| Pop7 | TI_6 | Alps | Tyrol | 115 | 115 | 111 | 111 | 196 | 196 | 304 | 304 | ***50*** | ***50*** | 153 | 153 | 161 | 163 | 244 | 246 | 128 | 130 | 109 | 109 | 180 | 180 | 157 | 159 |
| Pop8 | TI_7 | Alps | Tyrol | 115 | 115 | 108 | 111 | 198 | 198 | 304 | 304 | 122 | 124 | 150 | 150 | 163 | 165 | 240 | 242 | 128 | 130 | 109 | 111 | 180 | 180 | 157 | 159 |
| Pop8 | TI_7 | Alps | Tyrol | 115 | 130 | 108 | 111 | 196 | 196 | 304 | 304 | ***50*** | ***50*** | 153 | 153 | 161 | 163 | 240 | 240 | 130 | 138 | 109 | 109 | 188 | 190 | 159 | 167 |
| Pop8 | TI_7 | Alps | Tyrol | 115 | 115 | 108 | 108 | 196 | 196 | 304 | 304 | 122 | 122 | 150 | 153 | 163 | 165 | 240 | 240 | 128 | 130 | 109 | 109 | 188 | 190 | 167 | 167 |
| Pop8 | TI_7 | Alps | Tyrol | 120 | 130 | 111 | 111 | 196 | 198 | 304 | 304 | 120 | 120 | 153 | 153 | 161 | 163 | 240 | 242 | 130 | 138 | 109 | 109 | 188 | 190 | 159 | 167 |
| Pop8 | TI_7 | Alps | Tyrol | 115 | 115 | 111 | 111 | 198 | 198 | 304 | 304 | 120 | 120 | 150 | 153 | *50* | *50* | 240 | 240 | 128 | 128 | 109 | 109 | 180 | 184 | 159 | 159 |
| Pop8 | TI_7 | Alps | Tyrol | 115 | 115 | 111 | 111 | 198 | 198 | 304 | 304 | 120 | 120 | 150 | 153 | *50* | *50* | 240 | 240 | 128 | 128 | 109 | 109 | 180 | 184 | 159 | 159 |
| Pop8 | TI_7 | Alps | Tyrol | 115 | 115 | 111 | 111 | 196 | 196 | 304 | 304 | 122 | 122 | 153 | 153 | 163 | 165 | 240 | 240 | 128 | 130 | 109 | 109 | 180 | 188 | 159 | 167 |
| Pop8 | TI_7 | Alps | Tyrol | 115 | 115 | 111 | 111 | 196 | 196 | 304 | 304 | 120 | 122 | 153 | 153 | 163 | 165 | 240 | 240 | 128 | 130 | 109 | 109 | 180 | 180 | 157 | 159 |
| Pop8 | TI_7 | Alps | Tyrol | 115 | 130 | 105 | 111 | 196 | 196 | 304 | 304 | 122 | 124 | 153 | 153 | 163 | 167 | 240 | 242 | 128 | 130 | 109 | 109 | 184 | 190 | 157 | 159 |
| Pop8 | TI_7 | Alps | Tyrol | 115 | 115 | 111 | 111 | 196 | 198 | 304 | 304 | 122 | 122 | 153 | 153 | 163 | 167 | 240 | 240 | 128 | 130 | 109 | 109 | 180 | 190 | 157 | 159 |
| Pop8 | TI_7 | Alps | Tyrol | 120 | 130 | 111 | 111 | 196 | 198 | 304 | 304 | 122 | 124 | 153 | 153 | 161 | 163 | 240 | 240 | 128 | 130 | 109 | 109 | 180 | 190 | 157 | 159 |
| Pop8 | TI_7 | Alps | Tyrol | 120 | 130 | 111 | 111 | 196 | 198 | 304 | 304 | 122 | 124 | 153 | 153 | 161 | 163 | 240 | 242 | 130 | 138 | 109 | 109 | 180 | 190 | 159 | 167 |
| Pop8 | TI_7 | Alps | Tyrol | 120 | 130 | 111 | 111 | 196 | 198 | 304 | 304 | 122 | 124 | 153 | 153 | 161 | 163 | 240 | 240 | 140 | 142 | 109 | 109 | 180 | 184 | 157 | 157 |
| Pop9 | AH_1 | Alps | Ahrntal | 115 | 115 | 111 | 111 | 196 | 196 | 304 | 304 | 122 | 122 | 153 | 153 | 161 | 163 | 240 | 240 | 128 | 138 | 109 | 109 | 188 | 190 | 157 | 167 |
| Pop9 | AH_1 | Alps | Ahrntal | 115 | 115 | 111 | 111 | 196 | 198 | 304 | 304 | 124 | 124 | 153 | 153 | 161 | 163 | 240 | 240 | 136 | 138 | 109 | 109 | 186 | 188 | 157 | 171 |
| Pop9 | AH_1 | Alps | Ahrntal | 115 | 115 | 111 | 111 | 196 | 196 | 304 | 304 | 124 | 124 | 153 | 153 | 161 | 163 | 240 | 240 | 128 | 142 | 109 | 109 | 186 | 188 | 157 | 171 |
| Pop9 | AH_1 | Alps | Ahrntal | 120 | 130 | 111 | 111 | 196 | 196 | 304 | 304 | 122 | 124 | 153 | 153 | 161 | 163 | 240 | 242 | 128 | 142 | 109 | 109 | 174 | 190 | 157 | 171 |
| Pop9 | AH_1 | Alps | Ahrntal | 120 | 120 | 111 | 111 | 196 | 196 | 304 | 304 | 122 | 122 | 153 | 153 | 161 | 163 | 240 | 240 | 126 | 128 | 109 | 109 | 188 | 190 | 155 | 157 |
| Pop9 | AH_1 | Alps | Ahrntal | 115 | 115 | 108 | 111 | 196 | 198 | 304 | 304 | 122 | 122 | 153 | 153 | 163 | 165 | 240 | 240 | 128 | 130 | 109 | 109 | 174 | 174 | 157 | 159 |
| Pop9 | AH_1 | Alps | Ahrntal | 115 | 130 | 111 | 111 | 196 | 196 | 304 | 304 | 122 | 122 | 153 | 153 | 163 | 165 | 240 | 240 | 126 | 128 | 109 | 111 | 188 | 190 | 159 | 171 |
| Pop9 | AH_1 | Alps | Ahrntal | 115 | 120 | 111 | 111 | 196 | 198 | 304 | 304 | 122 | 122 | 153 | 153 | 163 | 165 | 240 | 240 | 126 | 128 | 109 | 109 | 180 | 190 | 155 | 157 |
| Pop9 | AH_1 | Alps | Ahrntal | 115 | 120 | 111 | 111 | 196 | 196 | 304 | 304 | 122 | 122 | 153 | 153 | 163 | 165 | 240 | 240 | 136 | 138 | 109 | 109 | 188 | 190 | 165 | 167 |
| Pop9 | AH_1 | Alps | Ahrntal | 115 | 115 | 111 | 111 | 196 | 196 | 304 | 319 | 122 | 122 | 153 | 153 | 163 | 165 | 240 | 240 | 126 | 128 | 109 | 109 | 174 | 174 | 155 | 157 |
| Pop9 | AH_1 | Alps | Ahrntal | 115 | 115 | 108 | 111 | 196 | 196 | 304 | 304 | 122 | 122 | 153 | 153 | 161 | 163 | 240 | 240 | 128 | 142 | 109 | 111 | 174 | 174 | 157 | 171 |
| Pop9 | AH_1 | Alps | Ahrntal | 115 | 115 | 111 | 111 | 196 | 196 | 304 | 304 | 122 | 122 | 153 | 153 | 161 | 163 | 240 | 240 | 126 | 128 | 109 | 109 | 174 | 174 | 155 | 157 |
| Pop9 | AH_1 | Alps | Ahrntal | 115 | 115 | 111 | 111 | 196 | 198 | 304 | 304 | 122 | 122 | 153 | 153 | 161 | 163 | 240 | 240 | ***50*** | ***50*** | 109 | 109 | 174 | 174 | 169 | 171 |
| Pop9 | AH_1 | Alps | Ahrntal | 115 | 115 | 111 | 111 | 196 | 196 | 304 | 304 | 124 | 124 | 153 | 153 | 161 | 163 | 240 | 240 | 140 | 142 | 109 | 109 | 186 | 188 | 169 | 171 |
| Pop9 | AH_1 | Alps | Ahrntal | 115 | 115 | 111 | 111 | 196 | 196 | 304 | 304 | 122 | 122 | 153 | 153 | 163 | 165 | 240 | 240 | 140 | 142 | 109 | 109 | 188 | 188 | 169 | 171 |
| Pop9 | AH_1 | Alps | Ahrntal | 115 | 115 | 111 | 111 | 196 | 196 | 304 | 304 | 122 | 122 | 153 | 153 | 163 | 165 | 240 | 240 | 140 | 142 | 109 | 109 | 186 | 188 | 169 | 171 |
| Pop10 | UM_1 | Alps | Umbaltal | 115 | 115 | 111 | 111 | 196 | 196 | 304 | 304 | 120 | 122 | 153 | 153 | 161 | 163 | 240 | 240 | 140 | 142 | 109 | 109 | 180 | 180 | 167 | 167 |
| Pop10 | UM_1 | Alps | Umbaltal | 115 | 115 | 111 | 111 | 196 | 196 | 304 | 304 | 122 | 122 | 153 | 153 | 161 | 163 | 240 | 240 | 126 | 128 | 109 | 109 | 184 | 184 | 155 | 157 |
| Pop10 | UM_1 | Alps | Umbaltal | 115 | 120 | 108 | 108 | 196 | 196 | 304 | 304 | 122 | 122 | 153 | 153 | 165 | 167 | 240 | 240 | 126 | 128 | 109 | 109 | 180 | 190 | 155 | 157 |
| Pop10 | UM_1 | Alps | Umbaltal | 115 | 120 | 111 | 111 | 196 | 196 | 304 | 304 | 122 | 122 | 153 | 153 | 165 | 165 | 240 | 240 | 128 | 138 | 109 | 109 | 186 | 186 | 157 | 167 |
| Pop10 | UM_1 | Alps | Umbaltal | 115 | 115 | 108 | 111 | 196 | 196 | 304 | 304 | 122 | 124 | 153 | 153 | 163 | 163 | 240 | 242 | 136 | 138 | 109 | 109 | 180 | 180 | 165 | 167 |
| Pop10 | UM_1 | Alps | Umbaltal | 115 | 120 | 108 | 111 | 196 | 198 | 304 | 304 | 122 | 122 | 153 | 153 | 161 | 163 | 240 | 240 | 126 | 128 | 109 | 109 | 184 | 190 | 157 | 167 |
| Pop10 | UM_1 | Alps | Umbaltal | 130 | 130 | 108 | 111 | 196 | 196 | 322 | 325 | 122 | 124 | 150 | 153 | ***161*** | ***163*** | 242 | 242 | 124 | 128 | 109 | 109 | 194 | 194 | 155 | 157 |
| Pop10 | UM_1 | Alps | Umbaltal | 120 | 120 | 111 | 111 | 196 | 196 | 304 | 304 | 122 | 124 | 153 | 153 | 163 | 165 | 240 | 242 | 128 | 132 | 109 | 109 | 188 | 190 | 155 | 157 |
| Pop10 | UM_1 | Alps | Umbaltal | 115 | 115 | 111 | 111 | 196 | 198 | 304 | 304 | 122 | 124 | 153 | 153 | 161 | 163 | 240 | 242 | 128 | 130 | 109 | 109 | 180 | 190 | 157 | 159 |
| Pop10 | UM_1 | Alps | Umbaltal | 120 | 120 | 111 | 111 | 196 | 196 | 304 | 304 | 122 | 124 | 153 | 153 | 161 | 163 | 240 | 242 | 126 | 128 | 109 | 109 | 180 | 190 | 155 | 157 |
| Pop10 | UM_1 | Alps | Umbaltal | 115 | 115 | 111 | 111 | 196 | 196 | 304 | 304 | 122 | 124 | 153 | 153 | 161 | 163 | 240 | 242 | 128 | 132 | 109 | 109 | 188 | 190 | 157 | 161 |
| Pop10 | UM_1 | Alps | Umbaltal | 115 | 115 | 111 | 111 | 196 | 196 | 304 | 304 | 122 | 122 | 153 | 153 | 161 | 163 | 240 | 240 | 130 | 132 | 109 | 109 | 180 | 180 | 159 | 161 |
| Pop11 | ST_1 | Alps | Styria | 130 | 135 | 111 | 111 | 194 | 194 | 304 | 304 | ***50*** | ***50*** | 153 | 153 | 161 | 163 | 240 | 242 | 136 | 138 | 109 | 109 | 188 | 190 | 165 | 167 |
| Pop11 | ST_1 | Alps | Styria | 115 | 115 | 111 | 111 | 194 | 194 | 304 | 304 | 122 | 124 | 153 | 153 | ***161*** | ***163*** | 240 | 242 | 136 | 138 | 109 | 109 | 188 | 190 | 165 | 167 |
| Pop11 | ST_1 | Alps | Styria | 130 | 130 | 111 | 111 | 194 | 198 | 304 | 316 | 122 | 124 | 153 | 153 | ***50*** | ***50*** | 240 | 242 | 136 | 138 | 109 | 109 | 188 | 190 | 165 | 167 |
| Pop11 | ST_1 | Alps | Styria | 130 | 135 | 108 | 111 | 196 | 196 | 304 | 304 | ***50*** | ***50*** | 153 | 153 | 161 | 165 | 240 | 242 | 136 | 138 | 109 | 111 | 188 | 190 | 165 | 167 |
| Pop11 | ST_1 | Alps | Styria | 130 | 130 | 111 | 111 | 196 | 196 | 304 | 304 | ***50*** | ***50*** | 153 | 153 | 159 | 165 | 240 | 242 | 136 | 138 | 109 | 111 | 188 | 190 | 165 | 167 |
| Pop11 | ST_1 | Alps | Styria | 130 | 130 | 111 | 111 | 196 | 196 | 304 | 304 | ***50*** | ***50*** | 153 | 153 | 159 | 165 | 240 | 242 | 136 | 138 | 109 | 111 | 188 | 190 | 165 | 167 |
| Pop11 | ST_1 | Alps | Styria | 130 | 130 | 111 | 111 | 198 | 198 | 304 | 304 | 122 | 124 | 153 | 153 | 163 | 165 | 240 | 242 | 136 | 138 | 109 | 109 | 174 | 190 | 165 | 167 |
| Pop11 | ST_1 | Alps | Styria | 120 | 130 | 111 | 111 | 194 | 198 | 304 | 304 | 122 | 124 | 153 | 153 | 163 | 165 | 240 | 242 | 136 | 138 | 109 | 111 | 188 | 190 | 165 | 167 |
| Pop11 | ST_1 | Alps | Styria | 130 | 130 | 111 | 111 | 196 | 198 | 304 | 304 | 120 | 124 | 153 | 153 | ***159*** | ***165*** | 240 | 242 | 136 | 138 | **50** | **50** | **188** | **190** | 165 | 167 |
| Pop11 | ST_1 | Alps | Styria | ***50*** | ***50*** | 111 | 111 | 198 | 198 | 304 | 304 | 122 | 124 | 153 | 153 | ***50*** | ***50*** | 240 | 242 | 136 | 138 | 109 | 109 | 174 | 190 | 165 | 167 |
| Pop11 | ST_1 | Alps | Styria | 130 | 130 | 111 | 111 | 196 | 196 | 304 | 304 | 122 | 124 | 153 | 153 | 165 | 167 | 240 | 242 | 136 | 138 | 109 | 109 | 188 | 190 | 165 | 167 |
| Pop11 | ST_1 | Alps | Styria | 110 | 115 | 111 | 111 | 196 | 198 | 304 | 304 | 122 | 126 | 153 | 153 | 163 | 165 | 240 | 244 | 138 | 142 | 109 | 109 | 188 | 190 | 167 | 171 |
| Pop12 | ST_2 | Alps | Styria | 130 | 160 | 108 | 111 | 194 | 194 | 304 | 304 | 120 | 122 | 153 | 153 | 161 | 165 | 240 | 242 | 134 | 136 | 103 | 103 | *50* | *50* | 165 | 167 |
| Pop12 | ST_2 | Alps | Styria | 130 | 130 | 111 | 111 | 194 | 196 | 304 | 304 | 120 | 122 | 153 | 153 | 163 | 165 | 240 | 242 | 134 | 136 | 109 | 111 | 188 | 190 | 165 | 167 |
| Pop12 | ST_2 | Alps | Styria | 120 | 130 | 111 | 111 | 194 | 196 | 304 | 304 | 122 | 126 | 153 | 153 | 163 | 165 | 242 | 246 | 134 | 136 | 109 | 109 | 188 | 190 | 165 | 167 |
| Pop12 | ST_2 | Alps | Styria | 130 | 160 | 111 | 111 | 194 | 196 | *304* | *304* | 122 | 122 | 153 | 153 | *163* | *165* | 240 | 242 | *134* | *136* | 109 | 109 | 188 | 190 | 165 | 167 |
| Pop12 | ST_2 | Alps | Styria | 130 | 160 | 108 | 111 | 194 | 194 | 304 | 304 | *120* | *122* | 153 | 153 | 161 | 165 | 240 | 242 | 134 | 136 | 103 | 103 | 180 | 180 | 165 | 167 |
| Pop12 | ST_2 | Alps | Styria | 130 | 130 | 108 | 111 | 194 | 194 | 304 | 316 | 120 | 124 | 153 | 153 | 161 | 163 | 240 | 244 | 134 | 136 | 109 | 109 | 188 | 190 | 165 | 167 |
| Pop12 | ST_2 | Alps | Styria | 115 | 130 | 108 | 111 | 196 | 196 | 304 | 316 | 120 | 122 | 153 | 153 | 161 | 165 | 240 | 242 | 134 | 136 | 109 | 111 | 188 | 190 | 165 | 167 |
| Pop12 | ST_2 | Alps | Styria | 130 | 130 | 111 | 111 | 194 | 194 | 304 | 304 | 122 | 124 | 153 | 153 | 163 | 165 | 242 | 244 | 134 | 136 | 109 | 111 | 188 | 190 | 165 | 167 |
| Pop12 | ST_2 | Alps | Styria | 130 | 130 | 111 | 111 | 194 | 194 | 304 | 316 | 122 | 126 | 153 | 153 | *163* | *165* | 242 | 246 | 134 | 136 | 109 | 109 | 188 | 190 | 165 | 167 |
| Pop12 | ST_2 | Alps | Styria | 130 | 130 | 111 | 111 | 194 | 194 | 304 | 304 | 120 | 122 | 153 | 153 | 153 | 153 | 240 | 242 | 134 | 136 | 109 | 111 | 188 | 190 | 165 | 167 |
| Pop12 | ST_2 | Alps | Styria | 130 | 160 | 111 | 111 | 194 | 194 | 304 | 304 | 120 | 122 | 153 | 153 | *163* | *165* | 240 | 242 | 134 | 136 | 109 | 109 | 188 | 190 | 165 | 167 |
| Pop12 | ST_2 | Alps | Styria | 130 | 130 | 111 | 111 | 194 | 194 | 304 | 304 | 120 | 122 | 153 | 153 | *163* | *165* | 240 | 242 | 134 | 136 | 109 | 109 | 180 | 190 | 165 | 167 |
| Pop12 | ST_2 | Alps | Styria | 130 | 130 | 108 | 111 | 194 | 196 | 304 | 304 | 120 | 122 | 153 | 153 | 163 | 165 | 240 | 242 | 132 | 134 | 109 | 109 | 186 | 188 | 165 | 167 |
| Pop13 | LB_1 | Alps | Lombardy | ***50*** | ***50*** | 108 | 108 | 196 | 196 | 304 | 304 | 124 | 128 | 153 | 153 | 163 | 165 | 242 | 246 | 134 | 140 | 109 | 109 | 174 | 180 | ***50*** | ***50*** |
| Pop13 | LB_1 | Alps | Lombardy | 115 | 120 | 108 | 111 | 196 | 198 | 304 | 304 | 120 | 124 | 153 | 153 | 163 | 165 | 238 | 242 | 132 | 144 | 109 | 109 | 174 | 190 | 161 | 173 |
| Pop13 | LB_1 | Alps | Lombardy | 115 | 120 | 108 | 108 | 196 | 198 | 304 | 304 | 124 | 128 | 153 | 153 | 163 | 165 | 242 | 246 | 142 | 144 | 109 | 109 | 180 | 190 | 171 | 173 |
| Pop13 | LB_1 | Alps | Lombardy | 120 | 120 | 111 | 111 | 196 | 196 | 304 | 304 | 124 | 128 | 153 | 153 | 163 | 165 | ***50*** | ***50*** | 140 | 142 | 109 | 109 | 174 | 190 | 169 | 171 |
| Pop13 | LB_1 | Alps | Lombardy | 115 | 120 | 111 | 111 | 196 | 198 | 304 | 304 | 124 | 128 | 153 | 153 | 163 | 165 | 242 | 246 | 132 | 140 | 109 | 109 | 174 | 190 | 161 | 161 |
| Pop13 | LB_1 | Alps | Lombardy | 115 | 145 | 108 | 108 | 196 | 196 | 304 | 319 | 122 | 124 | 153 | 153 | 163 | 165 | 240 | 242 | 140 | 142 | 109 | 109 | 174 | 180 | 169 | 171 |
| Pop13 | LB_1 | Alps | Lombardy | 115 | 120 | 108 | 108 | 196 | 196 | 304 | 304 | 124 | 128 | 153 | 153 | 161 | 163 | 242 | 246 | 140 | 142 | 109 | 109 | 174 | 190 | 169 | 171 |
| Pop13 | LB_1 | Alps | Lombardy | 115 | 120 | 108 | 111 | 196 | 198 | 304 | 304 | 122 | 124 | 153 | 153 | 163 | 165 | 240 | 242 | 130 | 132 | 109 | 109 | 174 | 174 | 161 | 161 |
| Pop13 | LB_1 | Alps | Lombardy | 115 | 120 | 108 | 111 | 196 | 196 | 304 | 304 | 122 | 124 | 153 | 153 | 159 | 163 | 240 | 242 | 132 | 142 | 109 | 109 | 174 | 174 | 161 | 161 |
| Pop13 | LB_1 | Alps | Lombardy | 115 | 120 | 108 | 108 | 196 | 196 | 304 | 304 | 122 | 124 | 153 | 153 | 161 | 163 | 240 | 242 | 132 | 142 | 109 | 109 | 180 | 190 | 161 | 161 |
| Pop13 | LB_1 | Alps | Lombardy | 130 | 130 | 105 | 108 | 196 | 196 | 304 | 304 | 124 | 128 | 153 | 153 | 159 | 163 | 242 | 246 | 142 | 144 | 109 | 109 | ***50*** | ***50*** | 161 | 161 |
| Pop13 | LB_1 | Alps | Lombardy | 115 | 145 | 108 | 108 | 196 | 196 | 304 | 319 | 122 | 124 | 153 | 153 | 163 | 165 | 240 | 244 | 140 | 142 | 109 | 109 | 174 | 180 | 169 | 171 |
| Pop13 | LB_1 | Alps | Lombardy | 115 | 120 | 108 | 108 | 196 | 196 | 304 | 304 | 124 | 128 | 153 | 153 | 161 | 163 | 242 | 246 | 140 | 142 | 109 | 109 | 174 | 190 | 169 | 171 |
| Pop14 | LB_2 | Alps | Lombardy | 115 | 120 | 108 | 111 | 196 | 198 | 304 | 304 | 120 | 128 | 153 | 153 | 163 | 165 | 238 | 246 | 128 | 142 | 109 | 109 | 174 | 174 | ***50*** | ***50*** |
| Pop14 | LB_2 | Alps | Lombardy | 120 | 130 | 108 | 108 | 196 | 198 | 304 | 304 | 124 | 128 | 153 | 153 | 161 | 163 | 242 | 246 | 132 | 142 | 109 | 109 | 174 | 180 | 161 | 171 |
| Pop14 | LB_2 | Alps | Lombardy | 120 | 120 | 111 | 111 | 196 | 198 | 304 | 304 | 124 | 126 | 153 | 153 | 159 | 163 | 242 | 244 | 140 | 142 | 109 | 109 | 190 | 190 | ***50*** | ***50*** |
| Pop14 | LB_2 | Alps | Lombardy | 115 | 130 | 111 | 111 | 196 | 196 | 304 | 304 | 124 | 128 | 153 | 153 | 161 | 163 | 242 | 246 | 132 | 142 | 109 | 109 | 174 | 180 | 161 | 171 |
| Pop14 | LB_2 | Alps | Lombardy | 115 | 120 | 111 | 111 | 196 | 196 | 304 | 304 | 126 | 128 | 153 | 153 | 163 | 163 | 244 | 246 | 142 | 144 | 109 | 109 | 174 | 190 | 171 | 173 |
| Pop14 | LB_2 | Alps | Lombardy | 115 | 120 | 108 | 108 | 196 | 196 | 304 | 304 | 122 | 128 | 153 | 153 | 161 | 163 | 240 | 246 | 138 | 140 | 109 | 109 | 174 | 174 | 167 | 169 |
| Pop14 | LB_2 | Alps | Lombardy | 115 | 115 | 108 | 111 | 198 | 198 | 304 | 304 | 120 | 128 | 153 | 153 | 161 | 163 | 242 | 246 | 134 | 142 | 109 | 109 | 174 | 174 | 163 | 171 |
| Pop14 | LB_2 | Alps | Lombardy | 115 | 115 | 108 | 111 | 196 | 196 | 319 | 319 | ***50*** | ***50*** | 153 | 153 | 161 | 163 | 238 | 242 | 138 | 140 | 109 | 109 | 174 | 190 | 167 | 169 |
| Pop14 | LB_2 | Alps | Lombardy | 115 | 115 | 108 | 111 | 198 | 198 | 304 | 304 | 120 | 128 | 153 | 153 | 161 | 163 | ***50*** | ***50*** | 134 | 142 | 109 | 109 | 174 | 174 | 165 | 167 |
| Pop14 | LB_2 | Alps | Lombardy | 120 | 120 | 111 | 111 | 194 | 196 | 304 | 319 | 120 | 128 | 153 | 153 | 163 | 165 | 238 | 246 | ***50*** | ***50*** | 109 | 109 | 174 | 174 | ***50*** | ***50*** |
| Pop14 | LB_2 | Alps | Lombardy | 115 | 130 | 108 | 108 | 196 | 198 | 304 | 304 | 126 | 128 | 153 | 153 | 163 | 165 | 244 | 246 | 140 | 144 | 109 | 109 | 174 | 174 | 50 | 50 |
| Pop14 | LB_2 | Alps | Lombardy | 115 | 115 | 108 | 111 | 198 | 198 | 304 | 304 | 120 | 128 | 153 | 153 | 161 | 163 | ***50*** | ***50*** | 134 | 142 | 109 | 109 | 174 | 174 | 163 | 171 |
| Pop15 | LB_3 | Alps | Lombardy | 120 | 120 | 108 | 111 | 196 | 196 | 304 | 319 | 122 | 124 | 153 | 153 | 159 | 159 | 240 | 242 | 128 | 140 | 109 | 109 | 174 | 174 | 157 | 169 |
| Pop15 | LB_3 | Alps | Lombardy | 120 | 120 | 108 | 111 | 198 | 198 | 304 | 319 | 122 | 124 | 153 | 153 | 161 | 163 | 240 | 242 | 140 | 142 | 109 | 109 | 174 | 174 | 169 | 171 |
| Pop15 | LB_3 | Alps | Lombardy | 115 | 130 | 111 | 111 | 196 | 198 | 304 | 304 | 122 | 124 | 153 | 153 | 159 | 163 | 240 | 242 | 132 | 142 | 109 | 109 | 174 | 174 | 161 | 171 |
| Pop15 | LB_3 | Alps | Lombardy | 115 | 120 | 108 | 111 | 196 | 198 | 304 | 304 | 126 | 128 | 153 | 153 | 163 | 163 | 244 | 246 | 132 | 142 | 109 | 109 | 174 | 174 | 161 | 171 |
| Pop15 | LB_3 | Alps | Lombardy | 115 | 120 | 108 | 108 | 196 | 196 | 319 | 319 | 124 | 126 | 153 | 153 | 163 | 165 | 242 | 244 | 140 | 144 | 109 | 109 | 174 | 174 | 171 | 173 |
| Pop15 | LB_3 | Alps | Lombardy | 115 | 115 | 108 | 108 | 198 | 198 | 304 | 304 | 126 | 128 | 153 | 153 | 163 | 165 | 244 | 246 | 130 | 132 | 109 | 109 | 180 | 180 | 161 | 161 |
| Pop15 | LB_3 | Alps | Lombardy | 115 | 115 | 111 | 111 | 194 | 198 | 304 | 304 | 126 | 128 | 153 | 153 | 163 | 165 | 244 | 246 | 134 | 142 | 109 | 109 | 174 | 180 | 163 | 171 |
| Pop15 | LB_3 | Alps | Lombardy | 145 | 145 | 108 | 108 | 196 | 196 | 304 | 304 | 122 | 124 | 153 | 153 | 163 | 165 | 242 | 242 | 138 | 140 | 109 | 109 | 190 | 190 | 167 | 169 |
| Pop15 | LB_3 | Alps | Lombardy | 115 | 115 | 111 | 111 | 196 | 198 | 304 | 304 | 122 | 124 | 150 | 153 | 163 | 165 | 240 | 242 | 140 | 142 | 109 | 109 | 174 | 174 | 169 | 171 |
| Pop15 | LB_3 | Alps | Lombardy | 115 | 120 | 111 | 111 | 196 | 196 | 319 | 319 | 124 | 126 | 153 | 153 | 165 | 165 | 242 | 244 | 140 | 144 | 109 | 109 | 174 | 174 | ***50*** | ***50*** |
| Pop15 | LB_3 | Alps | Lombardy | 115 | 115 | 108 | 108 | 196 | 196 | 304 | 304 | 124 | 128 | 153 | 153 | 161 | 163 | 242 | 246 | 126 | 128 | 109 | 109 | 174 | 180 | 167 | 169 |
| Pop15 | LB_3 | Alps | Lombardy | 120 | 120 | 108 | 108 | 196 | 196 | 304 | 319 | 124 | 128 | 150 | 153 | 163 | 165 | 242 | 246 | 140 | 142 | 109 | 109 | 174 | 174 | 169 | 171 |
| Pop15 | LB_3 | Alps | Lombardy | 115 | 120 | 111 | 111 | 196 | 198 | 304 | 304 | 122 | 124 | 153 | 153 | 163 | 165 | 242 | 242 | 128 | 142 | 109 | 109 | 174 | 190 | 161 | 171 |
| Pop15 | LB_3 | Alps | Lombardy | 115 | 120 | 111 | 111 | 198 | 198 | 304 | 304 | 120 | 124 | 153 | 153 | 161 | 163 | 238 | 242 | 132 | 140 | 109 | 109 | 174 | 180 | 157 | 157 |
| Pop15 | LB_3 | Alps | Lombardy | 120 | 120 | 108 | 111 | 196 | 196 | 304 | 319 | 124 | 124 | 153 | 153 | 159 | 165 | 240 | 242 | 128 | 140 | 109 | 109 | 174 | 174 | 157 | 169 |
| Pop16 | Sw_1 | Alps | Switzerland | 145 | 145 | 105 | 105 | 50 | 50 | 304 | 304 | 122 | 122 | 150 | 150 | 163 | 165 | 240 | 242 | 138 | 140 | 109 | 109 | 174 | 174 | 169 | 171 |
| Pop16 | Sw_1 | Alps | Switzerland | 115 | 130 | 108 | 111 | 50 | 50 | 304 | 304 | 122 | 126 | 153 | 153 | 163 | 165 | 242 | 242 | 132 | 140 | 109 | 109 | 174 | 190 | 163 | 171 |
| Pop16 | Sw_1 | Alps | Switzerland | 115 | 130 | 111 | 111 | 50 | 50 | 304 | 304 | 118 | 120 | 153 | 153 | 163 | 165 | 238 | 240 | 130 | 140 | 109 | 109 | 174 | 190 | 161 | 171 |
| Pop16 | Sw_1 | Alps | Switzerland | 120 | 130 | 111 | 111 | 196 | 196 | 304 | 304 | 124 | 124 | 153 | 153 | 159 | 165 | 240 | 242 | ***50*** | ***50*** | 109 | 109 | 174 | 190 | 171 | 171 |
| Pop16 | Sw_1 | Alps | Switzerland | 115 | 130 | 108 | 111 | 196 | 198 | 304 | 304 | 124 | 126 | 153 | 153 | 163 | 165 | 244 | 246 | 128 | 140 | 109 | 109 | 174 | 180 | 159 | 161 |
| Pop16 | Sw_1 | Alps | Switzerland | 115 | 130 | 108 | 111 | 196 | 198 | 304 | 304 | 122 | 124 | 150 | 153 | 159 | 165 | 240 | 242 | 134 | 142 | 109 | 109 | 174 | 190 | 163 | 171 |
| Pop16 | Sw_1 | Alps | Switzerland | 115 | 115 | 108 | 108 | 196 | 196 | 304 | 304 | 122 | 124 | 150 | 153 | 159 | 165 | 240 | 242 | 132 | 134 | 109 | 109 | 174 | 174 | 161 | 163 |
| Pop16 | Sw_1 | Alps | Switzerland | 115 | 130 | 108 | 108 | 196 | 198 | 304 | 304 | 122 | 124 | 153 | 153 | 163 | 165 | 240 | 242 | 132 | 134 | 109 | 109 | 174 | 184 | 161 | 163 |
| Pop16 | Sw_1 | Alps | Switzerland | 120 | 130 | 108 | 111 | 196 | 196 | 316 | 316 | 126 | 128 | 153 | 153 | 159 | 163 | 244 | 246 | 130 | 132 | 109 | 109 | 174 | 174 | 159 | 161 |
| Pop16 | Sw_1 | Alps | Switzerland | 115 | 130 | 108 | 111 | 196 | 198 | 304 | 304 | 122 | 124 | 153 | 153 | 163 | 165 | 240 | 242 | 130 | 132 | 109 | 109 | 180 | 188 | 159 | 161 |
| Pop16 | Sw_1 | Alps | Switzerland | 115 | 130 | 108 | 111 | 196 | 198 | 304 | 304 | 122 | 124 | 153 | 153 | 163 | 165 | 240 | 242 | 132 | 134 | 109 | 109 | 174 | 184 | 161 | 163 |
| Pop17 | Sw_2 | Alps | Switzerland | 115 | 115 | 108 | 108 | 194 | 194 | 304 | 304 | 122 | 124 | 153 | 153 | 163 | 165 | 240 | 242 | 138 | 140 | 109 | 109 | 174 | 174 | 167 | 169 |
| Pop17 | Sw_2 | Alps | Switzerland | 120 | 130 | 108 | 108 | 196 | 196 | 304 | 304 | 126 | 128 | 153 | 153 | 163 | 165 | 244 | 246 | 132 | 140 | 109 | 109 | 174 | 190 | 161 | 169 |
| Pop17 | Sw_2 | Alps | Switzerland | 115 | 130 | 108 | 108 | 196 | 196 | 316 | 316 | 126 | 128 | 153 | 153 | 163 | 165 | 244 | 246 | 130 | 132 | 109 | 109 | 174 | 190 | 159 | 161 |
| Pop17 | Sw_2 | Alps | Switzerland | 115 | 130 | 108 | 108 | 196 | 196 | 304 | 304 | 124 | 126 | 153 | 153 | 163 | 165 | 242 | 244 | 138 | 140 | 109 | 109 | 174 | 174 | 167 | 169 |
| Pop17 | Sw_2 | Alps | Switzerland | 120 | 120 | 108 | 108 | 196 | 196 | 304 | 304 | 126 | 128 | 153 | 153 | 163 | 165 | 244 | 246 | 130 | 132 | 109 | 109 | 174 | 174 | 159 | 161 |
| Pop17 | Sw_2 | Alps | Switzerland | 115 | 120 | 108 | 108 | 196 | 198 | 316 | 316 | 126 | 128 | 153 | 153 | 163 | 165 | 244 | 246 | 132 | 140 | 109 | 109 | 174 | 174 | 161 | 169 |
| Pop17 | Sw_2 | Alps | Switzerland | 115 | 115 | 108 | 108 | 196 | 196 | 304 | 304 | 124 | 126 | 153 | 153 | 163 | 165 | 242 | 244 | 132 | 142 | 109 | 109 | 174 | 174 | 161 | 171 |
| Pop17 | Sw_2 | Alps | Switzerland | 115 | 130 | 108 | 108 | 196 | 198 | 304 | 304 | 124 | 128 | 153 | 153 | 163 | 165 | 242 | 246 | 130 | 132 | 109 | 109 | 184 | 184 | 159 | 161 |
| Pop17 | Sw_2 | Alps | Switzerland | 115 | 115 | 108 | 108 | 50 | 50 | 304 | 304 | 126 | 128 | 153 | 153 | 50 | 50 | 246 | 246 | 130 | 132 | 109 | 109 | 184 | 184 | 159 | 161 |
| Pop17 | Sw_2 | Alps | Switzerland | 115 | 130 | 108 | 108 | 50 | 50 | 304 | 304 | 124 | 124 | 153 | 153 | 50 | 50 | 242 | 242 | 138 | 140 | 109 | 109 | 174 | 174 | 167 | 169 |
| Pop17 | Sw_2 | Alps | Switzerland | 115 | 115 | 108 | 108 | 196 | 196 | 304 | 304 | 126 | 128 | 153 | 153 | 50 | 50 | 246 | 246 | 138 | 140 | 109 | 109 | 184 | 184 | 167 | 169 |
| Pop17 | Sw_2 | Alps | Switzerland | 115 | 130 | 108 | 111 | 196 | 198 | 304 | 304 | 124 | 128 | 150 | 153 | *163* | *163* | 242 | 246 | 132 | 142 | 109 | 109 | 174 | 174 | 161 | 171 |
| Pop17 | Sw_2 | Alps | Switzerland | 115 | 130 | 108 | 108 | 50 | 50 | 304 | 304 | 124 | 124 | 153 | 153 | *165* | *165* | 240 | 242 | 132 | 140 | 109 | 109 | 174 | 188 | 161 | 169 |
| Pop18 | Sw_3 | Alps | Switzerland | 130 | 130 | 108 | 111 | 194 | 198 | 304 | 304 | 120 | 122 | 153 | 153 | 165 | 167 | 240 | 242 | 50 | 50 | 109 | 109 | 184 | 188 | 155 | 157 |
| Pop18 | Sw_3 | Alps | Switzerland | 115 | 115 | 108 | 108 | 198 | 198 | 304 | 304 | ***50*** | ***50*** | 153 | 153 | 163 | 165 | 240 | 242 | 132 | 132 | 109 | 109 | 174 | 174 | 169 | 171 |
| Pop18 | Sw_3 | Alps | Switzerland | 120 | 130 | 108 | 108 | 196 | 198 | 304 | 304 | 122 | 124 | 153 | 153 | 163 | 165 | 240 | 242 | 132 | 142 | 109 | 109 | 174 | 174 | 161 | 171 |
| Pop18 | Sw_3 | Alps | Switzerland | 120 | 130 | 105 | 108 | 196 | 198 | 304 | 304 | 122 | 124 | 153 | 153 | 163 | 165 | 240 | 242 | 132 | 142 | 109 | 109 | 174 | 174 | 161 | 171 |
| Pop18 | Sw_3 | Alps | Switzerland | 120 | 120 | 108 | 108 | 198 | 198 | 304 | 304 | 124 | 126 | 153 | 153 | 163 | 165 | 242 | 244 | 130 | 132 | 109 | 109 | 180 | 180 | 161 | 161 |
| Pop18 | Sw_3 | Alps | Switzerland | 115 | 120 | 108 | 114 | 198 | 198 | 304 | 304 | 122 | 124 | 153 | 153 | 163 | 165 | 242 | 244 | 128 | 130 | 109 | 109 | 180 | 180 | 159 | 161 |
| Pop18 | Sw_3 | Alps | Switzerland | 115 | 130 | 50 | 50 | 198 | 198 | 304 | 304 | 122 | 126 | 153 | 153 | 163 | 165 | 242 | 246 | 126 | 140 | 109 | 109 | 174 | 190 | 157 | 171 |
| Pop18 | Sw_3 | Alps | Switzerland | 115 | 130 | 108 | 108 | 198 | 198 | 304 | 304 | 120 | 122 | 150 | 153 | 163 | 165 | 240 | 242 | 126 | 128 | 109 | 109 | 174 | 190 | 157 | 169 |
| Pop18 | Sw_3 | Alps | Switzerland | 130 | 130 | 108 | 111 | 198 | 198 | 304 | 304 | 120 | 122 | 153 | 153 | 163 | 165 | 240 | 242 | 50 | 50 | 109 | 109 | 174 | 180 | 159 | 161 |
| Pop18 | Sw_3 | Alps | Switzerland | 115 | 120 | 108 | 108 | 198 | 198 | 304 | 304 | 122 | 124 | 153 | 153 | 161 | 163 | 240 | 242 | 130 | 132 | 109 | 109 | 174 | 174 | 159 | 161 |
| Pop19 | KR_1 | Czech | Krkonoše | 125 | 125 | 111 | 111 | 196 | 196 | 316 | 316 | 124 | 126 | 153 | 153 | 163 | 165 | 242 | 244 | 138 | 140 | 111 | 111 | 180 | 180 | 167 | 169 |
| Pop19 | KR_1 | Czech | Krkonoše | 125 | 125 | 111 | 111 | 196 | 196 | 304 | 304 | 122 | 122 | 153 | 153 | 165 | 165 | 240 | 240 | 138 | 140 | 109 | 111 | 180 | 180 | 167 | 169 |
| Pop19 | KR_1 | Czech | Krkonoše | 130 | 130 | 111 | 111 | 196 | 196 | 304 | 316 | 122 | 122 | 153 | 153 | 163 | 165 | 240 | 240 | 138 | 140 | 109 | 109 | 188 | 190 | 167 | 169 |
| Pop19 | KR_1 | Czech | Krkonoše | 125 | 125 | 111 | 111 | 196 | 196 | 316 | 316 | 124 | 126 | 153 | 153 | 163 | 165 | 242 | 244 | 138 | 140 | 109 | 111 | 180 | 180 | 167 | 169 |
| Pop19 | KR_1 | Czech | Krkonoše | 125 | 125 | 111 | 111 | 196 | 196 | 304 | 316 | 122 | 122 | 153 | 153 | 165 | 165 | 240 | 240 | 138 | 140 | 109 | 111 | 180 | 190 | 167 | 169 |
| Pop19 | KR_1 | Czech | Krkonoše | 130 | 130 | 111 | 111 | 196 | 196 | 304 | 304 | 122 | 122 | 153 | 153 | 163 | 165 | 240 | 240 | 138 | 140 | 109 | 109 | 188 | 190 | 167 | 169 |
| Pop19 | KR_1 | Czech | Krkonoše | 125 | 125 | 111 | 111 | 196 | 196 | 316 | 316 | 124 | 126 | 153 | 153 | 163 | 165 | 242 | 244 | 138 | 140 | 109 | 111 | 180 | 180 | 167 | 169 |
| Pop19 | KR_1 | Czech | Krkonoše | 125 | 125 | 111 | 111 | 196 | 196 | 316 | 316 | 124 | 126 | 153 | 153 | 165 | 165 | 242 | 244 | 138 | 140 | 109 | 111 | 180 | 180 | 167 | 169 |
| Pop19 | KR_1 | Czech | Krkonoše | 130 | 130 | 111 | 111 | 196 | 196 | 304 | 304 | 122 | 122 | 153 | 153 | 163 | 165 | 240 | 240 | 138 | 140 | 109 | 109 | 188 | 190 | 167 | 169 |
| Pop19 | KR_1 | Czech | Krkonoše | 130 | 130 | 111 | 111 | 196 | 196 | 304 | 304 | 122 | 126 | 153 | 153 | 165 | 165 | 240 | 244 | 138 | 140 | 109 | 109 | 188 | 190 | 167 | 169 |
| Pop19 | KR_1 | Czech | Krkonoše | 125 | 125 | 111 | 111 | 196 | 196 | 316 | 316 | 124 | 126 | 153 | 153 | 163 | 165 | 242 | 244 | 138 | 140 | 109 | 111 | 180 | 180 | 167 | 169 |
| Pop19 | KR_1 | Czech | Krkonoše | 125 | 130 | 111 | 111 | 196 | 196 | 316 | 316 | 122 | 126 | 153 | 153 | 163 | 165 | 240 | 244 | 138 | 140 | 109 | 111 | 180 | 190 | 167 | 169 |
| Pop19 | KR_1 | Czech | Krkonoše | 125 | 125 | 111 | 111 | 196 | 196 | 316 | 316 | 122 | 126 | 153 | 153 | 163 | 165 | 240 | 244 | 138 | 140 | 109 | 111 | 180 | 180 | 169 | 169 |
| Pop19 | KR_1 | Czech | Krkonoše | 125 | 125 | 111 | 111 | 196 | 196 | 316 | 316 | 124 | 126 | 153 | 153 | 163 | 165 | 242 | 244 | 138 | 140 | 109 | 111 | 180 | 180 | 167 | 169 |
| Pop19 | KR_1 | Czech | Krkonoše | 125 | 125 | 111 | 111 | 196 | 196 | 316 | 316 | 126 | 126 | 153 | 153 | 163 | 165 | 242 | 244 | 138 | 140 | 109 | 111 | 180 | 180 | 167 | 169 |
| Pop19 | KR_1 | Czech | Krkonoše | 125 | 125 | 111 | 111 | 196 | 196 | 316 | 316 | 124 | 126 | 153 | 153 | 163 | 165 | 242 | 244 | 138 | 140 | 109 | 111 | 180 | 180 | 167 | 169 |
| Pop19 | KR_1 | Czech | Krkonoše | 130 | 130 | 111 | 111 | 196 | 196 | 304 | 316 | 122 | 122 | 153 | 153 | 163 | 165 | 240 | 240 | 138 | 140 | 109 | 111 | 188 | 190 | 167 | 169 |
| Pop19 | KR_1 | Czech | Krkonoše | 125 | 125 | 111 | 111 | 196 | 196 | 316 | 316 | 124 | 126 | 153 | 153 | 163 | 165 | 242 | 244 | 138 | 140 | 109 | 111 | 180 | 180 | 167 | 169 |
| Pop19 | KR_1 | Czech | Krkonoše | 130 | 130 | 111 | 111 | 196 | 196 | 316 | 316 | 124 | 126 | 153 | 153 | 163 | 165 | 242 | 244 | 138 | 140 | 109 | 111 | 180 | 180 | 167 | 169 |
| Pop19 | KR_1 | Czech | Krkonoše | 125 | 125 | 111 | 111 | 196 | 196 | 316 | 316 | 124 | 126 | 153 | 153 | 163 | 165 | 242 | 244 | 138 | 140 | 109 | 111 | 180 | 180 | 167 | 169 |
| Pop20 | KR_2 | Czech | Krkonoše | 125 | 125 | 111 | 111 | 196 | 196 | 316 | 316 | 124 | 126 | 153 | 153 | 165 | 165 | 242 | 244 | 138 | 140 | 109 | 111 | 180 | 180 | 167 | 169 |
| Pop20 | KR_2 | Czech | Krkonoše | 125 | 125 | 111 | 111 | 196 | 196 | 316 | 316 | 124 | 126 | 153 | 153 | 163 | 165 | 242 | 244 | 138 | 140 | 109 | 111 | 180 | 180 | 167 | 169 |
| Pop20 | KR_2 | Czech | Krkonoše | 125 | 125 | 111 | 111 | 196 | 196 | 316 | 316 | 122 | 126 | 153 | 153 | 163 | 165 | 240 | 244 | 138 | 140 | 109 | 111 | 180 | 180 | 167 | 169 |
| Pop20 | KR_2 | Czech | Krkonoše | 125 | 125 | 111 | 111 | 196 | 196 | 316 | 316 | 124 | 126 | 153 | 153 | 163 | 165 | 242 | 244 | 138 | 140 | 109 | 111 | 180 | 180 | 169 | 169 |
| Pop20 | KR_2 | Czech | Krkonoše | 125 | 130 | 111 | 111 | 196 | 196 | 316 | 316 | 122 | 126 | 153 | 153 | 163 | 165 | 240 | 244 | 138 | 140 | 109 | 111 | 180 | 190 | 167 | 169 |
| Pop20 | KR_2 | Czech | Krkonoše | 125 | 125 | 111 | 111 | 196 | 196 | 316 | 316 | 124 | 126 | 153 | 153 | 163 | 165 | ***50*** | ***50*** | 138 | 140 | 109 | 111 | 180 | 180 | 167 | 169 |
| Pop20 | KR_2 | Czech | Krkonoše | 125 | 125 | 111 | 111 | 196 | 196 | 316 | 316 | 124 | 126 | 153 | 153 | 163 | 165 | ***50*** | ***50*** | 138 | 140 | 109 | 111 | 180 | 180 | 167 | 169 |
| Pop20 | KR_2 | Czech | Krkonoše | 125 | 125 | 111 | 111 | 196 | 196 | 316 | 316 | 124 | 126 | 153 | 153 | 163 | 165 | 240 | 244 | 138 | 140 | 109 | 111 | 180 | 180 | 169 | 169 |
| Pop20 | KR_2 | Czech | Krkonoše | 125 | 125 | 111 | 111 | 196 | 196 | 316 | 316 | 124 | 126 | 153 | 153 | 163 | 165 | 242 | 244 | 138 | 140 | 109 | 111 | 180 | 180 | 169 | 169 |
| Pop20 | KR_2 | Czech | Krkonoše | 125 | 125 | 111 | 111 | 196 | 196 | 316 | 316 | 124 | 126 | 153 | 153 | 163 | 165 | 242 | 244 | 138 | 140 | 109 | 111 | 180 | 180 | 167 | 169 |
| Pop20 | KR_2 | Czech | Krkonoše | 130 | 130 | 111 | 111 | 196 | 196 | 316 | 316 | 124 | 126 | 153 | 153 | 163 | 165 | 240 | 244 | 138 | 140 | 109 | 111 | 188 | 190 | 167 | 169 |
| Pop20 | KR_2 | Czech | Krkonoše | 125 | 130 | 111 | 111 | 196 | 196 | 316 | 316 | 122 | 126 | 153 | 153 | 163 | 165 | 240 | 244 | 138 | 140 | 109 | 111 | 180 | 190 | 167 | 169 |
| Pop20 | KR_2 | Czech | Krkonoše | 125 | 125 | 111 | 111 | 196 | 196 | 316 | 316 | 124 | 126 | 153 | 153 | 163 | 165 | 242 | 244 | 138 | 140 | 109 | 111 | 180 | 180 | 167 | 169 |
| Pop20 | KR_2 | Czech | Krkonoše | 125 | 125 | 111 | 111 | 196 | 196 | 316 | 316 | 124 | 126 | 153 | 153 | 163 | 165 | 242 | 244 | 138 | 140 | 109 | 111 | 180 | 180 | 167 | 169 |
| Pop20 | KR_2 | Czech | Krkonoše | 125 | 125 | 111 | 111 | 196 | 196 | 304 | 316 | 124 | 126 | 153 | 153 | 163 | 165 | 242 | 244 | 138 | 140 | 109 | 111 | 180 | 180 | 167 | 169 |
| Pop20 | KR_2 | Czech | Krkonoše | 125 | 125 | 111 | 111 | 196 | 196 | 316 | 316 | 124 | 126 | 153 | 153 | 163 | 165 | 242 | 244 | 138 | 140 | 109 | 111 | 180 | 180 | 167 | 169 |
| Pop20 | KR_2 | Czech | Krkonoše | 125 | 125 | 111 | 111 | 196 | 196 | 316 | 316 | 124 | 126 | 153 | 153 | 163 | 165 | 242 | 244 | 138 | 140 | 109 | 111 | 180 | 180 | 167 | 169 |
| Pop21 | KR_3 | Czech | Krkonoše | 125 | 125 | 111 | 111 | 196 | 196 | 316 | 316 | 124 | 126 | 153 | 153 | 163 | 165 | 242 | 244 | 138 | 140 | 109 | 111 | 180 | 180 | 167 | 169 |
| Pop21 | KR_3 | Czech | Krkonoše | 130 | 130 | 111 | 111 | 196 | 196 | 304 | 304 | 120 | 122 | 153 | 153 | 165 | 165 | 240 | 240 | 138 | 140 | 109 | 111 | 188 | 190 | 167 | 169 |
| Pop21 | KR_3 | Czech | Krkonoše | 125 | 125 | 111 | 111 | 196 | 196 | 316 | 316 | 124 | 126 | 153 | 153 | 163 | 165 | 242 | 244 | 138 | 140 | 109 | 111 | 180 | 180 | 167 | 169 |
| Pop21 | KR_3 | Czech | Krkonoše | 125 | 125 | 111 | 111 | 196 | 196 | 316 | 316 | 124 | 126 | 153 | 153 | 163 | 165 | 242 | 244 | 138 | 140 | 109 | 111 | 180 | 180 | 167 | 169 |
| Pop21 | KR_3 | Czech | Krkonoše | 125 | 125 | 111 | 111 | 196 | 196 | 316 | 316 | 124 | 126 | 153 | 153 | 163 | 165 | 242 | 244 | 138 | 140 | 109 | 111 | 180 | 180 | 167 | 169 |
| Pop21 | KR_3 | Czech | Krkonoše | 125 | 125 | 111 | 111 | 196 | 196 | 316 | 316 | 124 | 126 | 153 | 153 | 163 | 165 | 242 | 244 | 138 | 140 | 109 | 111 | 180 | 180 | 167 | 169 |
| Pop21 | KR_3 | Czech | Krkonoše | 125 | 125 | 111 | 111 | 196 | 196 | 316 | 316 | 122 | 124 | 153 | 153 | 163 | 165 | 240 | 244 | 138 | 140 | 109 | 111 | 180 | 180 | 167 | 169 |
| Pop21 | KR_3 | Czech | Krkonoše | 125 | 130 | 111 | 111 | 196 | 196 | 316 | 316 | 122 | 126 | 153 | 153 | 163 | 165 | 240 | 244 | 138 | 140 | 109 | 111 | 180 | 190 | 167 | 169 |
| Pop21 | KR_3 | Czech | Krkonoše | 125 | 125 | 111 | 111 | 196 | 196 | 316 | 316 | 124 | 126 | 153 | 153 | 163 | 165 | 242 | 244 | 138 | 140 | 109 | 111 | 180 | 180 | 167 | 169 |
| Pop21 | KR_3 | Czech | Krkonoše | 125 | 125 | 111 | 111 | 196 | 196 | 316 | 316 | 124 | 126 | 153 | 153 | 163 | 165 | 242 | 244 | 138 | 140 | 109 | 111 | 180 | 180 | 167 | 169 |
| Pop21 | KR_3 | Czech | Krkonoše | 125 | 125 | 111 | 111 | 196 | 196 | 316 | 316 | 122 | 126 | 153 | 153 | 163 | 165 | 240 | 244 | 138 | 140 | 109 | 111 | 180 | 180 | 167 | 169 |
| Pop21 | KR_3 | Czech | Krkonoše | 125 | 125 | 111 | 111 | 196 | 196 | 316 | 316 | 124 | 126 | 153 | 153 | 163 | 165 | 242 | 244 | 138 | 140 | 109 | 111 | 180 | 180 | 167 | 169 |
| Pop21 | KR_3 | Czech | Krkonoše | 130 | 130 | 111 | 111 | 196 | 196 | 304 | 304 | 122 | 122 | 153 | 153 | 163 | 165 | 240 | 240 | 138 | 140 | 109 | 111 | 188 | 190 | 167 | 169 |
| Pop21 | KR_3 | Czech | Krkonoše | 125 | 125 | 111 | 111 | 196 | 196 | 316 | 316 | 124 | 126 | 153 | 153 | 163 | 165 | ***50*** | ***50*** | 138 | 140 | 109 | 111 | 180 | 180 | 167 | 169 |
| Pop21 | KR_3 | Czech | Krkonoše | 125 | 130 | 111 | 111 | 196 | 196 | 316 | 316 | 124 | 126 | 153 | 153 | 165 | 165 | 242 | 244 | 138 | 140 | 109 | 111 | 188 | 190 | 167 | 169 |
| Pop21 | KR_3 | Czech | Krkonoše | 130 | 130 | 111 | 111 | 196 | 196 | 304 | 304 | 122 | 122 | 153 | 153 | 163 | 165 | 240 | 240 | 138 | 140 | 109 | 111 | 188 | 190 | 167 | 169 |
| Pop21 | KR_3 | Czech | Krkonoše | ***50*** | ***50*** | 111 | 111 | 196 | 196 | ***50*** | ***50*** | 122 | 122 | 153 | 153 | 163 | 165 | 240 | 240 | 138 | 140 | 109 | 111 | 188 | 190 | 167 | 169 |
| Pop21 | KR_3 | Czech | Krkonoše | ***50*** | ***50*** | 111 | 111 | 196 | 196 | ***50*** | ***50*** | 124 | 126 | 153 | 153 | 163 | 165 | 242 | 244 | 138 | 140 | 109 | 111 | 180 | 190 | 167 | 169 |
| Pop21 | KR_3 | Czech | Krkonoše | 130 | 130 | 111 | 111 | 196 | 196 | 304 | 304 | 122 | 122 | 153 | 153 | 165 | 165 | 240 | 240 | 138 | 140 | 109 | 111 | 188 | 190 | 167 | 169 |
| Pop22 | JH_1 | Czech | Jizerky | 125 | 125 | 111 | 111 | 196 | 196 | 316 | 316 | 124 | 126 | 153 | 153 | 163 | 165 | 242 | 244 | 138 | 140 | 109 | 111 | 180 | 180 | 169 | 169 |
| Pop22 | JH_1 | Czech | Jizerky | 125 | 125 | 111 | 111 | 196 | 196 | 316 | 316 | 124 | 126 | 153 | 153 | 163 | 165 | 242 | 244 | 138 | 140 | 109 | 111 | 180 | 180 | 167 | 169 |
| Pop22 | JH_1 | Czech | Jizerky | 125 | 125 | 111 | 111 | 196 | 196 | 316 | 316 | 124 | 126 | 153 | 153 | 163 | 165 | 242 | 244 | 136 | 138 | 109 | 111 | 180 | 180 | 165 | 167 |
| Pop22 | JH_1 | Czech | Jizerky | 125 | 125 | 111 | 111 | 196 | 196 | 316 | 316 | 124 | 126 | 153 | 153 | 163 | 165 | 242 | 244 | 138 | 140 | 109 | 111 | 180 | 180 | 167 | 169 |
| Pop22 | JH_1 | Czech | Jizerky | 125 | 125 | 111 | 111 | 196 | 196 | 316 | 316 | 124 | 126 | 153 | 153 | 163 | 165 | 242 | 244 | 136 | 138 | 109 | 111 | 180 | 180 | 165 | 167 |
| Pop22 | JH_1 | Czech | Jizerky | 125 | 125 | 111 | 111 | 196 | 196 | 316 | 316 | 124 | 126 | 153 | 153 | 163 | 165 | 242 | 244 | 138 | 140 | 109 | 111 | 180 | 180 | 167 | 169 |
| Pop22 | JH_1 | Czech | Jizerky | 125 | 125 | 111 | 111 | 196 | 196 | 316 | 316 | 124 | 126 | 153 | 153 | 163 | 165 | 242 | 244 | 138 | 140 | 109 | 111 | 180 | 180 | 167 | 169 |
| Pop22 | JH_1 | Czech | Jizerky | 125 | 125 | 111 | 111 | 196 | 196 | 316 | 316 | 124 | 126 | 153 | 153 | 163 | 165 | 242 | 244 | 136 | 138 | 109 | 111 | 180 | 180 | 165 | 167 |
| Pop22 | JH_1 | Czech | Jizerky | 125 | 125 | 111 | 111 | 196 | 196 | 316 | 316 | 122 | 122 | 153 | 153 | 163 | 165 | 240 | 240 | 138 | 140 | 109 | 111 | 180 | 180 | 167 | 169 |
| Pop22 | JH_1 | Czech | Jizerky | 125 | 125 | 111 | 111 | 196 | 196 | 316 | 316 | 124 | 126 | 153 | 153 | 163 | 165 | 242 | 244 | 138 | 140 | 109 | 111 | 180 | 180 | 167 | 169 |
| Pop22 | JH_1 | Czech | Jizerky | 125 | 125 | 111 | 111 | 196 | 196 | 316 | 316 | 124 | 126 | 153 | 153 | 163 | 165 | 242 | 244 | 138 | 140 | 109 | 111 | 180 | 180 | 167 | 169 |
| Pop22 | JH_1 | Czech | Jizerky | 125 | 125 | 111 | 111 | 196 | 196 | 316 | 316 | 124 | 126 | 153 | 153 | 163 | 165 | 242 | 244 | 138 | 140 | 109 | 111 | 180 | 180 | 167 | 169 |
| Pop22 | JH_1 | Czech | Jizerky | 125 | 125 | 111 | 111 | 196 | 196 | 316 | 316 | 124 | 126 | 153 | 153 | 163 | 165 | 242 | 244 | 138 | 140 | 109 | 111 | 180 | 180 | 167 | 169 |
| Pop22 | JH_1 | Czech | Jizerky | 120 | 125 | 111 | 111 | 196 | 196 | 316 | 316 | 124 | 126 | 153 | 153 | 163 | 165 | 242 | 244 | 138 | 140 | 109 | 111 | 180 | 180 | 167 | 169 |
| Pop23 | OH_1 | Czech | Orlicke | 115 | 115 | 111 | 111 | 196 | 196 | *50* | *50* | 126 | 128 | 153 | 153 | 163 | 165 | 244 | 246 | 138 | 140 | 109 | 109 | 174 | 174 | 167 | 169 |
| Pop23 | OH_1 | Czech | Orlicke | 115 | 115 | 111 | 111 | 196 | 196 | *50* | *50* | 126 | 128 | 153 | 153 | 163 | 165 | 244 | 246 | 138 | 140 | 109 | 109 | 174 | 174 | 167 | 169 |
| Pop23 | OH_2 | Czech | Orlicke | 115 | 115 | 111 | 111 | 196 | 196 | *50* | *50* | 126 | 128 | 153 | 153 | 163 | 165 | 244 | 246 | 138 | 140 | 109 | 109 | 174 | 174 | 167 | 169 |
| Pop23 | OH_1 | Czech | Orlicke | 115 | 115 | 111 | 111 | 196 | 196 | *50* | *50* | 126 | 128 | 153 | 153 | 163 | 165 | 244 | 246 | 138 | 140 | 109 | 109 | 174 | 174 | 167 | 169 |
| Pop23 | OH_1 | Czech | Orlicke | 115 | 115 | 111 | 111 | 196 | 196 | *50* | *50* | 126 | 128 | 153 | 153 | 163 | 165 | 244 | 246 | 138 | 140 | 109 | 109 | 174 | 174 | 167 | 169 |
| Pop23 | OH_1 | Czech | Orlicke | 115 | 115 | 111 | 111 | 196 | 196 | *50* | *50* | 126 | 128 | 153 | 153 | 163 | 165 | 244 | 246 | 138 | 140 | 109 | 109 | 174 | 174 | 167 | 169 |
| Pop23 | OH_1 | Czech | Orlicke | 115 | 115 | 111 | 111 | 196 | 196 | *50* | *50* | 126 | 128 | 153 | 153 | 163 | 165 | 244 | 246 | 138 | 140 | 109 | 109 | 174 | 174 | 167 | 169 |
| Pop23 | OH_1 | Czech | Orlicke | 115 | 115 | 111 | 111 | 196 | 196 | *50* | *50* | 126 | 128 | 153 | 153 | 163 | 165 | 244 | 246 | 138 | 140 | 109 | 109 | 174 | 174 | 167 | 169 |
| Pop23 | OH_1 | Czech | Orlicke | 115 | 115 | 111 | 111 | 196 | 196 | *50* | *50* | 126 | 128 | 153 | 153 | 163 | 165 | 244 | 246 | 138 | 140 | 109 | 109 | 174 | 174 | 167 | 169 |
| Pop23 | OH_1 | Czech | Orlicke | 115 | 115 | 111 | 111 | 196 | 196 | *50* | *50* | 126 | 128 | 153 | 153 | 163 | 165 | 244 | 246 | 138 | 140 | 109 | 109 | 174 | 174 | 167 | 169 |
| Pop23 | OH_1 | Czech | Orlicke | 115 | 115 | 111 | 111 | 196 | 196 | *50* | *50* | 126 | 128 | 153 | 153 | 163 | 165 | 244 | 246 | 138 | 140 | 109 | 109 | 174 | 174 | 167 | 169 |
| Pop23 | OH_1 | Czech | Orlicke | 115 | 115 | 111 | 111 | 196 | 196 | *50* | *50* | 126 | 128 | 153 | 153 | 163 | 165 | 244 | 246 | 138 | 140 | 109 | 109 | 174 | 174 | 167 | 169 |
| Pop23 | OH_1 | Czech | Orlicke | 115 | 115 | 111 | 111 | 196 | 196 | *50* | *50* | 126 | 128 | 153 | 153 | 163 | 165 | 244 | 246 | 138 | 140 | 109 | 109 | 174 | 174 | 167 | 169 |
| Pop23 | OH_1 | Czech | Orlicke | 115 | 115 | 111 | 111 | 196 | 196 | *50* | *50* | 126 | 128 | 153 | 153 | 163 | 165 | 244 | 246 | 138 | 140 | 109 | 109 | 174 | 174 | 167 | 169 |
| Pop23 | OH_1 | Czech | Orlicke | 115 | 115 | 111 | 111 | 196 | 196 | *50* | *50* | 126 | 128 | 153 | 153 | 163 | 165 | 244 | 246 | 138 | 140 | 109 | 109 | 174 | 174 | 167 | 169 |
| Pop23 | OH_1 | Czech | Orlicke | 115 | 115 | 111 | 111 | 196 | 196 | *50* | *50* | 126 | 128 | 153 | 153 | 163 | 165 | 244 | 246 | 138 | 140 | 109 | 109 | 174 | 174 | 167 | 169 |
| Pop23 | OH_1 | Czech | Orlicke | 115 | 115 | 111 | 111 | 196 | 196 | *50* | *50* | 126 | 128 | 153 | 153 | 163 | 165 | 244 | 246 | 138 | 140 | 109 | 109 | 174 | 174 | 167 | 169 |
| Pop23 | OH_1 | Czech | Orlicke | 115 | 115 | 111 | 111 | 196 | 196 | *50* | *50* | 126 | 128 | 153 | 153 | 163 | 165 | 244 | 246 | 138 | 140 | 109 | 109 | 174 | 174 | 167 | 169 |
| Pop24 | CRE_1 | Carpathian | East | 125 | 130 | 111 | 111 | 194 | 194 | 310 | 310 | 122 | 124 | 150 | 150 | 149 | 149 | 240 | 242 | 128 | 144 | 109 | 109 | 182 | 186 | 163 | 169 |
| Pop24 | CRE_1 | Carpathian | East | 120 | 120 | 111 | 111 | 194 | 194 | 310 | 310 | 124 | 126 | 150 | 150 | 149 | 149 | 242 | 244 | 136 | 144 | 109 | 109 | 186 | 198 | 165 | 165 |
| Pop24 | CRE_1 | Carpathian | East | 120 | 120 | 111 | 111 | 194 | 194 | 316 | 316 | 126 | 128 | 150 | 150 | 149 | 149 | 244 | 246 | 134 | 138 | 109 | 109 | 200 | 202 | 163 | 167 |
| Pop24 | CRE_1 | Carpathian | East | 130 | 130 | 111 | 111 | 194 | 194 | 310 | 316 | 124 | 126 | 150 | 150 | 149 | 149 | 242 | 244 | 136 | 138 | 109 | 109 | 184 | 186 | 165 | 167 |
| Pop24 | CRE_1 | Carpathian | East | 110 | 120 | 111 | 111 | 194 | 194 | 310 | 316 | 120 | 122 | 150 | 150 | 149 | 149 | 240 | 240 | 134 | 142 | 109 | 109 | 186 | 188 | 167 | 169 |
| Pop24 | CRE_1 | Carpathian | East | 120 | 130 | 111 | 111 | 194 | 194 | 310 | 322 | 122 | 132 | 150 | 150 | 149 | 149 | 240 | 250 | 142 | 144 | 109 | 109 | 186 | 190 | ***50*** | ***50*** |
| Pop24 | CRE_1 | Carpathian | East | 115 | 115 | 111 | 111 | 196 | 198 | 304 | 304 | 122 | 124 | 153 | 153 | 163 | 165 | 240 | 242 | 128 | 138 | 109 | 109 | 188 | 190 | 157 | 167 |
| Pop24 | CRE_1 | Carpathian | East | 115 | 120 | 111 | 114 | 194 | 194 | 310 | 316 | 128 | 130 | 150 | 150 | 149 | 149 | 246 | 248 | 136 | 142 | 109 | 109 | 186 | 188 | 167 | 171 |
| Pop24 | CRE_1 | Carpathian | East | 100 | 100 | 111 | 111 | 194 | 194 | 310 | 316 | 126 | 130 | 150 | 150 | 149 | 149 | 244 | 248 | 134 | 144 | 109 | 109 | 174 | 174 | 163 | 167 |
| Pop24 | CRE_1 | Carpathian | East | 120 | 130 | 108 | 111 | 194 | 194 | 310 | 316 | 128 | 130 | 150 | 150 | 149 | 159 | 246 | 248 | 136 | 138 | 109 | 109 | 174 | 188 | 165 | 167 |
| Pop24 | CRE_1 | Carpathian | East | 120 | 130 | 111 | 111 | 194 | 194 | 310 | 310 | 126 | 130 | 150 | 150 | 149 | 159 | 244 | 248 | 126 | 136 | 109 | 109 | 186 | 188 | 163 | 171 |
| Pop24 | CRE_1 | Carpathian | East | 125 | 125 | 111 | 111 | 194 | 194 | 310 | 310 | ***50*** | ***50*** | 153 | 153 | 149 | 149 | 242 | 244 | ***50*** | ***50*** | 109 | 109 | 184 | 184 | ***50*** | ***50*** |
| Pop24 | CRE_1 | Carpathian | East | 120 | 120 | 108 | 111 | 194 | 194 | 316 | 319 | 122 | 126 | 150 | 150 | 149 | 149 | 240 | 244 | 136 | 138 | 109 | 109 | 186 | 188 | 165 | 167 |
| Pop24 | CRE_1 | Carpathian | East | 110 | 120 | 111 | 111 | 194 | 194 | 310 | 310 | 126 | 132 | 150 | 150 | 149 | 149 | 244 | 250 | 118 | 120 | 109 | 109 | 186 | 190 | 149 | 149 |
| Pop24 | CRE_1 | Carpathian | East | 120 | 120 | 111 | 111 | 194 | 194 | 310 | 310 | 124 | 126 | 150 | 150 | 149 | 159 | 242 | 244 | 136 | 142 | 109 | 109 | 174 | 186 | 165 | 171 |
| Pop24 | CRE_1 | Carpathian | East | 130 | 130 | 111 | 114 | 194 | 194 | 310 | 316 | 126 | 128 | 150 | 150 | 149 | 149 | 244 | 246 | 138 | 142 | 109 | 109 | 174 | 174 | 157 | 165 |
| Pop25 | CRW_2 | Carpathian | West | 100 | 100 | 111 | 111 | 194 | 194 | 310 | 316 | 124 | 126 | 150 | 150 | 149 | 149 | 242 | 244 | 134 | 136 | 109 | 109 | ***50*** | ***50*** | 163 | 165 |
| Pop25 | CRW_2 | Carpathian | West | 120 | 120 | 111 | 111 | 194 | 196 | 310 | 325 | 126 | 130 | 150 | 150 | 149 | 149 | 244 | 248 | 136 | 138 | 109 | 109 | 186 | 188 | 165 | 167 |
| Pop25 | CRW_2 | Carpathian | West | 120 | 120 | 111 | 111 | 194 | 194 | 310 | 310 | 124 | 126 | 150 | 153 | 149 | 149 | 242 | 244 | 134 | 136 | 109 | 111 | 186 | 188 | 163 | 165 |
| Pop25 | CRW_2 | Carpathian | West | 120 | 120 | 108 | 114 | 194 | 194 | 325 | 325 | 118 | 122 | 150 | 150 | 153 | 153 | 236 | 240 | 124 | 138 | 109 | 109 | 182 | 184 | 153 | 167 |
| Pop25 | CRW_2 | Carpathian | West | 120 | 120 | 102 | 102 | 194 | 194 | 310 | 310 | 124 | 126 | 150 | 150 | 149 | 149 | 242 | 244 | 134 | 136 | 109 | 109 | 188 | 188 | 163 | 165 |
| Pop25 | CRW_2 | Carpathian | West | 115 | 120 | 108 | 108 | 194 | 194 | 301 | 301 | 122 | 124 | 150 | 150 | 153 | 153 | 240 | 242 | 128 | 134 | 109 | 109 | 182 | 184 | 157 | 163 |
| Pop25 | CRW_2 | Carpathian | West | 120 | 120 | 108 | 108 | 194 | 194 | 310 | 310 | 122 | 124 | 150 | 150 | 149 | 153 | 240 | 242 | 124 | 124 | 109 | 109 | 182 | 184 | 153 | 153 |
| Pop25 | CRW_2 | Carpathian | West | 115 | 120 | 108 | 108 | 194 | 194 | 301 | 322 | 122 | 122 | 150 | 150 | 149 | 153 | 240 | 240 | 126 | 128 | 109 | 109 | 182 | 184 | 155 | 157 |
| Pop25 | CRW_2 | Carpathian | West | 120 | 120 | 108 | 108 | 194 | 194 | 322 | 322 | 122 | 122 | 150 | 150 | 153 | 153 | 240 | 240 | 134 | 138 | 109 | 109 | 200 | 202 | 163 | 167 |
| Pop25 | CRW_2 | Carpathian | West | 120 | 120 | 111 | 111 | 194 | 194 | 310 | 310 | 126 | 130 | 150 | 150 | 149 | 165 | 244 | 248 | 128 | 136 | 109 | 109 | 186 | 188 | 157 | 165 |
| Pop25 | CRW_2 | Carpathian | West | 120 | 120 | 111 | 111 | 194 | 194 | 325 | 325 | 124 | 126 | 150 | 150 | 149 | 149 | 242 | 244 | 134 | 136 | 109 | 109 | 186 | 188 | 163 | 165 |
| Pop25 | CRW_2 | Carpathian | West | 120 | 120 | 102 | 102 | 194 | 194 | 325 | 325 | 124 | 126 | 150 | 150 | 149 | 149 | 242 | 244 | 134 | 136 | 109 | 109 | 186 | 188 | 163 | 165 |
| Pop25 | CRW_2 | Carpathian | West | 120 | 120 | 102 | 102 | 194 | 194 | 310 | 310 | 126 | 126 | 150 | 150 | 149 | 149 | 242 | 244 | 134 | 136 | 109 | 109 | 188 | 188 | 163 | 165 |
| Pop25 | CRW_2 | Carpathian | West | 120 | 125 | 108 | 111 | 194 | 194 | 310 | 310 | 126 | 130 | 150 | 150 | 149 | 149 | 244 | 248 | 136 | 138 | 109 | 109 | 186 | 188 | 165 | 167 |
| Pop25 | CRW_2 | Carpathian | West | 100 | 120 | 102 | 111 | 194 | 194 | 310 | 325 | 126 | 130 | 150 | 150 | 149 | 149 | 244 | 248 | 134 | 136 | 109 | 109 | 186 | 188 | 163 | 165 |
| Pop26 | BL_1 | Balkan | St.Planina | 110 | 125 | 108 | 108 | 194 | 194 | 301 | 319 | 122 | 122 | 150 | 150 | 153 | 153 | 240 | 240 | 134 | 138 | 109 | 109 | 188 | 196 | 163 | 167 |
| Pop26 | BL_1 | Balkan | St.Planina | 120 | 120 | 108 | 111 | 194 | 194 | 316 | 316 | 122 | 124 | 150 | 150 | 153 | 153 | 240 | 242 | 130 | 132 | 109 | 109 | 182 | 184 | 159 | 161 |
| Pop26 | BL_1 | Balkan | St.Planina | 125 | 125 | 108 | 108 | 194 | 194 | 316 | 319 | 122 | 122 | 150 | 150 | 153 | 153 | 240 | 240 | 130 | 138 | 109 | 109 | 174 | 198 | 159 | 163 |
| Pop26 | BL_1 | Balkan | St.Planina | 120 | 125 | 108 | 111 | 194 | 194 | 310 | 310 | 124 | 134 | 150 | 150 | 149 | 149 | 242 | 252 | 130 | 138 | 109 | 109 | 194 | 196 | 161 | 167 |
| Pop26 | BL_1 | Balkan | St.Planina | 125 | 125 | 108 | 108 | 194 | 194 | 301 | 319 | 122 | 122 | 150 | 150 | 149 | 153 | 240 | 240 | 130 | 134 | 109 | 109 | 188 | 194 | 159 | 163 |
| Pop26 | BL_1 | Balkan | St.Planina | 120 | 125 | 108 | 108 | 194 | 194 | 316 | 322 | 122 | 126 | 150 | 150 | 153 | 153 | 240 | 244 | 124 | 138 | 109 | 109 | 184 | 196 | 153 | 167 |
| Pop26 | BL_1 | Balkan | St.Planina | 110 | 110 | 108 | 111 | 194 | 194 | 316 | 316 | 122 | 126 | 150 | 150 | 153 | 155 | 240 | 244 | 132 | 134 | 109 | 109 | 174 | 184 | ***50*** | ***50*** |
| Pop26 | BL_1 | Balkan | St.Planina | 115 | 125 | 108 | 108 | 194 | 194 | 310 | 319 | 118 | 118 | 150 | 150 | 153 | 153 | 236 | 236 | 134 | 138 | 109 | 109 | 196 | 202 | 163 | 167 |
| Pop26 | BL_1 | Balkan | St.Planina | 120 | 120 | 108 | 111 | 194 | 194 | 304 | 319 | 122 | 124 | 150 | 150 | 149 | 155 | 240 | 242 | 134 | 138 | 109 | 109 | 182 | 184 | 163 | 167 |
| Pop26 | BL_1 | Balkan | St.Planina | ***50*** | ***50*** | 108 | 108 | 194 | 194 | 316 | 319 | 112 | 122 | 150 | 150 | 153 | 155 | 230 | 240 | 132 | 134 | 109 | 109 | 186 | 188 | 161 | 163 |
| Pop26 | BL_1 | Balkan | St.Planina | 120 | 125 | 108 | 111 | 194 | 194 | 301 | 304 | 122 | 122 | 150 | 150 | 151 | 153 | 240 | 240 | 132 | 134 | 109 | 109 | 196 | 202 | 161 | 163 |
| Pop26 | BL_1 | Balkan | St.Planina | 125 | 125 | 108 | 111 | 194 | 194 | 301 | 319 | 122 | 122 | 150 | 150 | 151 | 155 | 240 | 240 | 132 | 136 | 109 | 109 | 186 | 188 | 161 | 163 |
| Pop26 | BL_1 | Balkan | St.Planina | 125 | 125 | 108 | 108 | 194 | 194 | 316 | 319 | ***50*** | ***50*** | 150 | 150 | 149 | 155 | 240 | 252 | 132 | 136 | 109 | 109 | 194 | 196 | 161 | 163 |
| Pop26 | BL_1 | Balkan | St.Planina | 125 | 125 | 111 | 111 | 194 | 194 | 316 | 322 | 122 | 122 | 150 | 150 | 153 | 155 | 240 | 240 | 130 | 138 | 109 | 109 | ***50*** | ***50*** | 163 | 167 |
| Pop27 | BL_2 | Balkan | Vitosha | 110 | 120 | 108 | 108 | 194 | 194 | 322 | 325 | 122 | 124 | 150 | 150 | 151 | 153 | 240 | 242 | 120 | 120 | 109 | 109 | 186 | 198 | 149 | 149 |
| Pop27 | BL_2 | Balkan | Vitosha | 110 | 115 | 108 | 108 | 194 | 194 | 319 | 322 | 124 | 126 | 150 | 150 | 155 | 155 | 242 | 244 | 138 | 138 | 109 | 109 | 196 | 198 | 165 | 167 |
| Pop27 | BL_2 | Balkan | Vitosha | 115 | 120 | 108 | 108 | 194 | 194 | 325 | 325 | 122 | 122 | 150 | 150 | 151 | 153 | 240 | 240 | 130 | 132 | 109 | 109 | 190 | 198 | 159 | 161 |
| Pop27 | BL_2 | Balkan | Vitosha | ***50*** | ***50*** | 108 | 108 | 194 | 194 | 319 | 319 | 122 | 126 | 150 | 150 | 155 | 155 | 240 | 244 | ***50*** | ***50*** | 109 | 109 | 196 | 198 | 149 | 161 |
| Pop27 | BL_2 | Balkan | Vitosha | 115 | 115 | 108 | 108 | 194 | 194 | 319 | 325 | 122 | 126 | 150 | 150 | 151 | 155 | 240 | 244 | 134 | 138 | 109 | 109 | 198 | 202 | 163 | 167 |
| Pop27 | BL_2 | Balkan | Vitosha | 110 | 120 | 108 | 108 | 194 | 194 | 301 | 301 | 122 | 122 | 150 | 150 | 155 | 155 | 240 | 240 | 126 | 130 | 109 | 109 | 188 | 190 | 157 | 161 |
| Pop27 | BL_2 | Balkan | Vitosha | 110 | 120 | 108 | 108 | 194 | 194 | 301 | 316 | ***50*** | ***50*** | 150 | 150 | 151 | 155 | 240 | 242 | 128 | 130 | 109 | 109 | 188 | 190 | 159 | 161 |
| Pop27 | BL_2 | Balkan | Vitosha | 115 | 115 | 108 | 108 | 194 | 194 | 301 | 301 | 124 | 126 | 150 | 150 | 153 | 155 | 242 | 244 | 130 | 140 | 109 | 109 | 190 | 198 | 159 | 169 |
| Pop27 | BL_2 | Balkan | Vitosha | 115 | 115 | 108 | 108 | 194 | 194 | 319 | 325 | 122 | 126 | 150 | 150 | 153 | 155 | 240 | 244 | 130 | 134 | 109 | 109 | 186 | 190 | 159 | 159 |
| Pop27 | BL_2 | Balkan | Vitosha | 115 | 120 | 108 | 108 | 194 | 194 | 310 | 325 | 122 | 123 | 150 | 150 | ***50*** | ***50*** | 240 | 244 | 138 | 140 | 109 | 109 | 198 | 202 | 163 | 167 |
| Pop27 | BL_2 | Balkan | Vitosha | 115 | 120 | 108 | 108 | 194 | 194 | 325 | 325 | 122 | 122 | 150 | 150 | 151 | 155 | 240 | 240 | 126 | 130 | 109 | 109 | 190 | 198 | 155 | 159 |
| Pop28 | BL_3 | Balkan | Rila | 115 | 125 | 108 | 108 | 194 | 194 | 50 | 50 | 124 | 134 | 150 | 150 | 153 | 153 | 230 | 230 | ***50*** | ***50*** | 109 | 109 | 196 | 198 | 163 | 167 |
| Pop28 | BL_3 | Balkan | Rila | 115 | 125 | 108 | 108 | 194 | 194 | 316 | 316 | 112 | 112 | 150 | 150 | 153 | 153 | 230 | 230 | 124 | 124 | 109 | 109 | 200 | 202 | 153 | 153 |
| Pop28 | BL_3 | Balkan | Rila | 110 | 110 | 108 | 108 | 194 | 194 | 301 | 301 | 136 | 138 | 150 | 150 | 155 | 155 | 240 | 240 | 134 | 136 | 109 | 109 | 196 | 198 | 163 | 165 |
| Pop28 | BL_3 | Balkan | Rila | ***50*** | ***50*** | 111 | 111 | 194 | 194 | 316 | 319 | 124 | 126 | 150 | 150 | ***50*** | ***50*** | 242 | 244 | 124 | 132 | 109 | 109 | 186 | 188 | 153 | 161 |
| Pop28 | BL_3 | Balkan | Rila | 115 | 125 | 108 | 108 | 194 | 194 | 301 | 301 | 112 | 112 | 150 | 150 | 149 | 149 | 230 | 230 | ***50*** | ***50*** | 109 | 109 | 196 | 198 | 161 | 163 |
| Pop28 | BL_3 | Balkan | Rila | 110 | 110 | 108 | 108 | 194 | 194 | 301 | 319 | 128 | 124 | 150 | 150 | 151 | 155 | 230 | 240 | 132 | 134 | 109 | 109 | 182 | 184 | 161 | 163 |
| Pop28 | BL_3 | Balkan | Rila | 115 | 115 | 108 | 108 | 194 | 194 | 301 | 319 | 122 | 124 | 150 | 150 | 149 | 149 | 240 | 240 | 124 | 124 | 109 | 109 | 184 | 186 | 153 | 153 |
| Pop28 | BL_3 | Balkan | Rila | 115 | 115 | 108 | 108 | 194 | 194 | 301 | 325 | 132 | 134 | 150 | 150 | 151 | 151 | 230 | 230 | 140 | 142 | 109 | 109 | 184 | 186 | 169 | 171 |
| Pop28 | BL_3 | Balkan | Rila | 115 | 115 | 108 | 108 | 194 | 194 | 301 | 325 | 132 | 134 | 150 | 150 | 151 | 151 | 230 | 230 | 140 | 142 | 109 | 109 | 184 | 186 | 169 | 171 |
| Pop28 | BL_3 | Balkan | Rila | 125 | 125 | 108 | 108 | 194 | 194 | 310 | 316 | 126 | 132 | 150 | 150 | 149 | 149 | 244 | 250 | 132 | 134 | 109 | 109 | 186 | 188 | 161 | 163 |
| Pop28 | BL_3 | Balkan | Rila | 115 | 115 | 108 | 108 | 194 | 194 | 301 | 301 | 122 | 122 | 150 | 150 | 153 | 153 | 240 | 240 | 128 | 134 | 109 | 109 | 186 | 188 | 157 | 163 |
| Pop28 | BL_3 | Balkan | Rila | 125 | 125 | 108 | 108 | 194 | 194 | 301 | 325 | 112 | 122 | 150 | 150 | 153 | 153 | ***50*** | ***50*** | 138 | 140 | 109 | 109 | 196 | 198 | 167 | 169 |
| Pop28 | BL_3 | Balkan | Rila | 115 | 115 | 108 | 108 | 194 | 194 | 301 | 301 | 112 | 112 | 150 | 150 | 151 | 151 | 230 | 230 | 134 | 136 | 109 | 109 | 198 | 200 | 163 | 165 |
| Pop28 | BL_3 | Balkan | Rila | 115 | 115 | 108 | 108 | 194 | 194 | 301 | 301 | 112 | 122 | 150 | 150 | 153 | 153 | 230 | 240 | 134 | 138 | 109 | 109 | 196 | 198 | 163 | 167 |
| Pop29 | BL_4 | Balkan | Pirin | 115 | 115 | 108 | 108 | 194 | 194 | 301 | 322 | ***50*** | ***50*** | 150 | 150 | 149 | 153 | 240 | 240 | ***50*** | ***50*** | 109 | 109 | 182 | 184 | 157 | 169 |
| Pop29 | BL_4 | Balkan | Pirin | 115 | 115 | 108 | 108 | 194 | 194 | 301 | 322 | 122 | 122 | 150 | 150 | 153 | 153 | 240 | 240 | 128 | 140 | 109 | 109 | 182 | 184 | 157 | 169 |
| Pop29 | BL_4 | Balkan | Pirin | 120 | 120 | 108 | 108 | 194 | 194 | 301 | 322 | 122 | 122 | 150 | 150 | 149 | 149 | 240 | 240 | 138 | 140 | 109 | 109 | 182 | 182 | 167 | 169 |
| Pop29 | BL_4 | Balkan | Pirin | 120 | 120 | 108 | 114 | 194 | 194 | 301 | 301 | 118 | 122 | 150 | 150 | ***50*** | ***50*** | 236 | 240 | 124 | 138 | 109 | 109 | 182 | 184 | 153 | 165 |
| Pop29 | BL_4 | Balkan | Pirin | 115 | 120 | 108 | 108 | 194 | 194 | 301 | 301 | 122 | 124 | 150 | 150 | 153 | 153 | 240 | 242 | 128 | 134 | ***50*** | ***50*** | 182 | 184 | 159 | 163 |
| Pop29 | BL_4 | Balkan | Pirin | 115 | 120 | 108 | 108 | 194 | 194 | 301 | 322 | 122 | 122 | 150 | 150 | 155 | 155 | 240 | 240 | 128 | 140 | 109 | 109 | 182 | 194 | 157 | 169 |
| Pop29 | BL_4 | Balkan | Pirin | 120 | 120 | 108 | 108 | 194 | 194 | 301 | 301 | 122 | 124 | 150 | 150 | 149 | 153 | 240 | 242 | 124 | 124 | 109 | 109 | 182 | 184 | 153 | 153 |
| Pop29 | BL_4 | Balkan | Pirin | 115 | 120 | 108 | 108 | 194 | 194 | 301 | 322 | 122 | 122 | 150 | 150 | 153 | 153 | 240 | 240 | 128 | 128 | 109 | 109 | 182 | 184 | 159 | 159 |
| Pop29 | BL_4 | Balkan | Pirin | 120 | 120 | 108 | 108 | 194 | 194 | 322 | 322 | 122 | 122 | 150 | 150 | 153 | 153 | 240 | 240 | 134 | 138 | ***50*** | ***50*** | 200 | 202 | 163 | 167 |
| Pop29 | BL_4 | Balkan | Pirin | 115 | 120 | 108 | 108 | 194 | 194 | 301 | 322 | 122 | 122 | 150 | 150 | 149 | 153 | 240 | 240 | 126 | 128 | 109 | 109 | 182 | 184 | 155 | 157 |
| Pop29 | BL_4 | Balkan | Pirin | 115 | 120 | 108 | 108 | 194 | 194 | 301 | 322 | 122 | 122 | 150 | 150 | 153 | 153 | 240 | 240 | 126 | 128 | 109 | 109 | 182 | 184 | 155 | 157 |
| Pop29 | BL_4 | Balkan | Pirin | 115 | 120 | 108 | 108 | 194 | 194 | 301 | 325 | 122 | 122 | 150 | 150 | 151 | 153 | 240 | 240 | 124 | 134 | 109 | 109 | 196 | 198 | 153 | 163 |
| Pop29 | BL_4 | Balkan | Pirin | 115 | 120 | 108 | 108 | 194 | 194 | 322 | 322 | 122 | 122 | 150 | 150 | 153 | 155 | 240 | 240 | ***50*** | ***50*** | 109 | 109 | 194 | 194 | 161 | 169 |
| Pop30 | IB_1 | Pyrenees | Prats | *50* | *50* | 111 | 111 | 196 | 196 | 325 | 325 | *50* | *50* | 150 | 150 | *155* | *155* | ***50*** | ***50*** | ***50*** | ***50*** | 109 | 109 | 166 | 166 | 155 | 157 |
| Pop30 | IB_1 | Pyrenees | Prats | 115 | 115 | 111 | 111 | 196 | 196 | 325 | 325 | 118 | 118 | 153 | 153 | 155 | 161 | 236 | 254 | 126 | 128 | 109 | 109 | 174 | 174 | 155 | 157 |
| Pop30 | IB_1 | Pyrenees | Prats | 125 | 125 | 111 | 111 | 196 | 196 | 325 | 325 | *50* | *50* | 150 | 150 | *155* | *155* | ***50*** | ***50*** | 126 | 128 | 109 | 111 | 166 | 166 | *50* | *50* |
| Pop30 | IB_1 | Pyrenees | Prats | 115 | 115 | 111 | 111 | 196 | 196 | 304 | 325 | 128 | 138 | 153 | 153 | 161 | 163 | 254 | 256 | 122 | 128 | 109 | 109 | 174 | 174 | 151 | 157 |
| Pop30 | IB_1 | Pyrenees | Prats | 115 | 115 | 111 | 111 | 196 | 196 | 325 | 325 | 136 | 138 | 153 | 153 | 161 | 163 | 254 | 256 | 138 | 140 | 109 | 109 | 174 | 180 | 167 | 169 |
| Pop30 | IB_1 | Pyrenees | Prats | 115 | 115 | 111 | 111 | 196 | 196 | 325 | 325 | 136 | 138 | 153 | 153 | ***50*** | ***50*** | ***50*** | ***50*** | 128 | 138 | 109 | 109 | 174 | 180 | 157 | 167 |
| Pop30 | IB_1 | Pyrenees | Prats | 115 | 115 | 111 | 111 | 196 | 196 | 325 | 325 | 118 | 118 | 153 | 153 | 153 | 155 | 236 | 256 | 128 | 128 | 109 | 109 | 174 | 174 | 50 | 50 |
| Pop30 | IB_1 | Pyrenees | Prats | 115 | 130 | 111 | 111 | 196 | 196 | 304 | 325 | 124 | 138 | 153 | 153 | 163 | 165 | 236 | 256 | *50* | *50* | 109 | 109 | 174 | 174 | 155 | 157 |
| Pop30 | IB_1 | Pyrenees | Prats | 115 | 115 | 111 | 111 | 196 | 196 | 325 | 325 | 134 | 138 | 153 | 153 | ***50*** | ***50*** | ***50*** | ***50*** | 122 | 138 | 109 | 109 | 174 | 174 | 151 | 167 |
| Pop30 | IB_1 | Pyrenees | Prats | 115 | 115 | 111 | 111 | 196 | 196 | 325 | 325 | 134 | 138 | 153 | 153 | ***50*** | ***50*** | ***50*** | ***50*** | 122 | 122 | 109 | 109 | 174 | 174 | 151 | 151 |
| Pop30 | IB_1 | Pyrenees | Prats | 115 | 115 | 111 | 111 | 196 | 196 | 325 | 325 | ***50*** | ***50*** | 153 | 153 | ***50*** | ***50*** | 254 | 256 | 122 | 128 | 109 | 109 | 174 | 174 | 151 | 157 |
| Pop30 | IB_1 | Pyrenees | Prats | 115 | 115 | 111 | 111 | 194 | 196 | 325 | 325 | 132 | 134 | 153 | 153 | 155 | 163 | 250 | 252 | 126 | 128 | 109 | 109 | 174 | 180 | 155 | 157 |
| Pop31 | IB_2 | Pyrenees | Aigues | 115 | 115 | 111 | 111 | 196 | 196 | 325 | 325 | 130 | 132 | 153 | 153 | 161 | 163 | 252 | 256 | 126 | 126 | 109 | 109 | 174 | 174 | 157 | 157 |
| Pop31 | IB_2 | Pyrenees | Aigues | 115 | 115 | 111 | 111 | 196 | 196 | 325 | 325 | 130 | 132 | 153 | 153 | 161 | 163 | 236 | 242 | 126 | 126 | 109 | 109 | 174 | 180 | 157 | 157 |
| Pop31 | IB_2 | Pyrenees | Aigues | 115 | 115 | 111 | 111 | 196 | 196 | 325 | 325 | 130 | 132 | 153 | 153 | 161 | 163 | 250 | 252 | 126 | 126 | 109 | 109 | 180 | 180 | 157 | 157 |
| Pop31 | IB_2 | Pyrenees | Aigues | 115 | 115 | 102 | 102 | 194 | 196 | 325 | 325 | 130 | 132 | 153 | 153 | 161 | 163 | 236 | 252 | 122 | 128 | 109 | 109 | 174 | 174 | 157 | 157 |
| Pop31 | IB_2 | Pyrenees | Aigues | 115 | 115 | 111 | 111 | ***50*** | ***50*** | 325 | 325 | 132 | 136 | 153 | 153 | 155 | 163 | 252 | 256 | 126 | 136 | 109 | 109 | 174 | 180 | 157 | 167 |
| Pop31 | IB_2 | Pyrenees | Aigues | 115 | 115 | 108 | 108 | 196 | 196 | 325 | 325 | 136 | 138 | 153 | 153 | 155 | 163 | 254 | 256 | 126 | 128 | 109 | 109 | 174 | 180 | 157 | 157 |
| Pop31 | IB_2 | Pyrenees | Aigues | 115 | 115 | 111 | 111 | 196 | 196 | 322 | 325 | 136 | 138 | 153 | 153 | 161 | 163 | 254 | 256 | 126 | 126 | 109 | 109 | 174 | 174 | 157 | 157 |
| Pop31 | IB_2 | Pyrenees | Aigues | 115 | 115 | 111 | 111 | 196 | 196 | 325 | 325 | 134 | 138 | 153 | 153 | 155 | 163 | 252 | 256 | 122 | 128 | 109 | 109 | 174 | 180 | 151 | 157 |
| Pop31 | IB_2 | Pyrenees | Aigues | 115 | 115 | 111 | 111 | 196 | 196 | 325 | 325 | 134 | 138 | 153 | 153 | 161 | 163 | 252 | 256 | 126 | 128 | 109 | 109 | 174 | 174 | 155 | 157 |
| Pop31 | IB_2 | Pyrenees | Aigues | 115 | 115 | 111 | 111 | 196 | 196 | 322 | 325 | 136 | 138 | 153 | 153 | 161 | 163 | 254 | 256 | 126 | 128 | 109 | 109 | 174 | 174 | 155 | 157 |
| Pop31 | IB_2 | Pyrenees | Aigues | 115 | 115 | 111 | 111 | 196 | 196 | 325 | 325 | 134 | 138 | 153 | 153 | 161 | 163 | 252 | 256 | 122 | 122 | 109 | 109 | 174 | 174 | 151 | 167 |
| Pop31 | IB_2 | Pyrenees | Aigues | 115 | 115 | 111 | 111 | 196 | 196 | 325 | 325 | 134 | 138 | 153 | 153 | 161 | 163 | 252 | 256 | 128 | 138 | 109 | 109 | 174 | 180 | 157 | 167 |
| Pop31 | IB_2 | Pyrenees | Aigues | 115 | 115 | 111 | 111 | 196 | 196 | 325 | 325 | 134 | 138 | 153 | 153 | 161 | 163 | 252 | 256 | 122 | 138 | 109 | 109 | 174 | 174 | 151 | 167 |
| Pop31 | IB_2 | Pyrenees | Aigues | 115 | 115 | 111 | 111 | 196 | 196 | 325 | 325 | 134 | 138 | 153 | 153 | 161 | 163 | 252 | 256 | 128 | 128 | 109 | 109 | 174 | 180 | 157 | 167 |
